# A cerebellar-thalamocortical pathway drives behavioral context-dependent movement initiation

**DOI:** 10.1016/j.neuron.2021.05.016

**Published:** 2021-07-21

**Authors:** Joshua Dacre, Matt Colligan, Thomas Clarke, Julian J. Ammer, Julia Schiemann, Victor Chamosa-Pino, Federico Claudi, J. Alex Harston, Constantinos Eleftheriou, Janelle M.P. Pakan, Cheng-Chiu Huang, Adam W. Hantman, Nathalie L. Rochefort, Ian Duguid

**Affiliations:** 1 Centre for Discovery Brain Sciences, Edinburgh Medical School: Biomedical Sciences, University of Edinburgh, Edinburgh, UK; 2 Simons Initiative for the Developing Brain, Centre for Discovery Brain Sciences, University of Edinburgh, Edinburgh, UK; 3 Janelia Research Campus, HHMI, Ashburn, VA, USA

**Keywords:** motor cortex, movement initiation, motor thalamus, cerebellar thalamocortical pathway, behavioural context, 2-photon imaging, *in vivo* patch-clamp, photoactivation, cerebellar nuclei, motor timing

## Abstract

Executing learned motor behaviors often requires the transformation of sensory cues into patterns of motor commands that generate appropriately timed actions. The cerebellum and thalamus are two key areas involved in shaping cortical output and movement, but the contribution of a cerebellar-thalamocortical pathway to voluntary movement initiation remains poorly understood. Here, we investigated how an auditory “go cue” transforms thalamocortical activity patterns and how these changes relate to movement initiation. Population responses in dentate/interpositus-recipient regions of motor thalamus reflect a time-locked increase in activity immediately prior to movement initiation that is temporally uncoupled from the go cue, indicative of a fixed-latency feedforward motor timing signal. Blocking cerebellar or motor thalamic output suppresses movement initiation, while stimulation triggers movements in a behavioral context-dependent manner. Our findings show how cerebellar output, via the thalamus, shapes cortical activity patterns necessary for learned context-dependent movement initiation.

## Highlights

• DN/IPN thalamocortical activity conveys a reliable feedforward motor timing signal
• Silencing DN/IPN or recipient regions of thalamus blocks movement initiation
• Photostimulation of the DN/IPN thalamocortical pathway triggers movement
• Thalamocortical activation drives behavioral context-dependent movement initiation

## Graphical abstract

**Figure:**
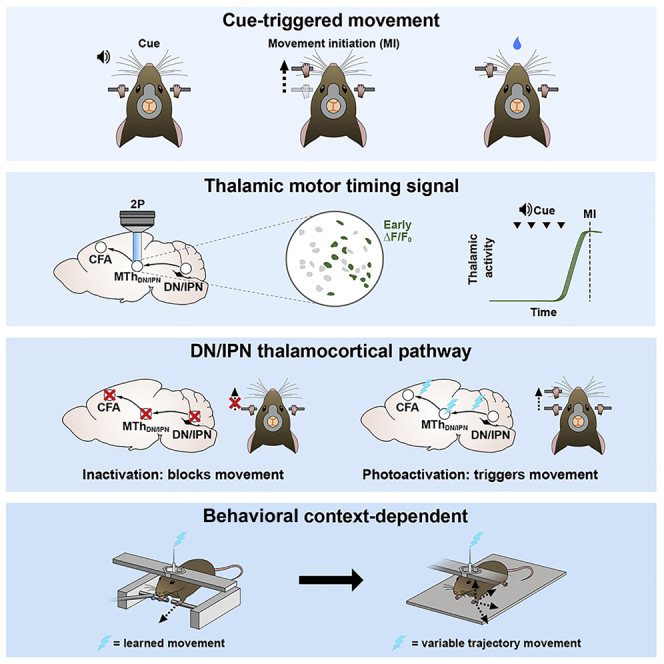

## Teaser

Dacre et al. show the contribution of a cerebellar-thalamocortical pathway to movement initiation. Using gain- and loss-of-function manipulations they demonstrate that output from dentate/interpositus nuclei, via the thalamus, shapes cortical activity dynamics necessary for learned behavioral context-dependent movement initiation.

## Introduction

The ability to generate appropriately timed motor actions in response to sensory cues is a hallmark of mammalian motor control. Movement timing is controlled in part by the cerebellum, as dysfunction leads to the execution of poorly timed actions (Bastian and Thach, 1995; Holmes, 1939; Milak et al., 1997; Thach, 1975). However, the pathway and circuit dynamics involved in initiating movements remain unclear. Two distinct pathways could contribute to movement initiation, the cerebellar-rubrospinal tract (Asanuma et al., 1983; Gibson et al., 1985; Teune et al., 2000) or the cerebellar-thalamocortical pathway (Bostan et al., 2013; Gornati et al., 2018; Horne and Butler, 1995; Kuramoto et al., 2009; Nashef et al., 2019). The latter is supported by neuronal activity in dentate/interpositus nuclei (DN/IPN) and recipient motor thalamic regions preceding cortical activity (Nashef et al., 2018; Strick, 1976; Thach, 1975, 2014) and movement initiation (Anderson and Turner, 1991; Butler et al., 1992, 1996; Fortier et al., 1989; Harvey et al., 1979; Horne and Porter, 1980; Kurata, 2005; Macpherson et al., 1980; Mushiake and Strick, 1993; Schmied et al., 1979; Strick, 1976; van Donkelaar et al., 1999), while disrupting activity in either region alters the timing of sensory-triggered actions (Meyer-Lohmann et al., 1977; Nashef et al., 2019; Spidalieri et al., 1983; Thach, 1975; van Donkelaar et al., 2000). Beyond a proposed role in movement initiation, DN/IPN and recipient regions of motor thalamus coordinate the timing and accuracy of ongoing movements given that focal inactivation alters endpoint accuracy (dysmetria/hypermetria), reach path curvature and grasping (Becker and Person, 2019; Bracha et al., 1999; Butler et al., 1992; Cooper et al., 2000; Horne and Butler, 1995; Ishikawa et al., 2014; Martin et al., 2000; Mason et al., 1998; Thanawalla et al., 2020), and loss of anticipatory limb adjustments to unexpected obstacles during complex locomotion (Martin et al., 2000; Milak et al., 1997). In contrast, disrupting output from fastigial nucleus results in deficits in posture, locomotion, and motor planning, with minimal effects on reaching (Li et al., 2015; Martin et al., 2000; Thach and Bastian, 2004). Thus, individual cerebellar nuclei provide differing contributions to movement control, where DN/IPN likely convey motor timing signals via thalamus to cortex in order to initiate and modify ongoing movements (Kurata, 2005; Nashef et al., 2018; Thach, 2014).

In rodents, cerebellar nuclei project to different regions of ventral thalamus. The fastigial nucleus primarily targets ventromedial (VM), while DN/IPN target the anteromedial (AM), and ventral anterolateral (VAL) subdivisions (Angaut et al., 1985; Gornati et al., 2018; Haroian et al., 1981; Kuramoto et al., 2009; Teune et al., 2000). DN/IPN axon terminal fields overlap substantially displaying morphological and functional characteristics consistent with strong feedforward driver inputs, such as large synaptic boutons (Aumann and Horne, 1996a, b; Aumann et al., 1994; Gornati et al., 2018) and large unitary responses (Gornati et al., 2018; Sawyer et al., 1994; Schäfer et al., 2021). Cerebellar input drives short-latency spiking in thalamic neurons that project to superficial and deep layers of motor cortex (Hooks et al., 2013; Kuramoto et al., 2009; Schäfer et al., 2021), transforming output via top-down excitation through layer 2/3 (Weiler et al., 2008) or direct excitation of layer 5 (Hooks et al., 2013; Sauerbrei et al., 2020). Key remaining questions are whether ventral motor thalamus plays a role in movement initiation and whether this is dependent on cerebellar input.

To address these questions, we developed a cued lever push task for mice requiring execution of a basic stimulus-response behavior for reward. This habitual behavior depends on antecedent stimuli rather than goal value, likely recruiting feedback reinforcement circuits, including VAL thalamus (Balleine, 2019; Graybiel, 2008). Using imaging, electrophysiology, and gain- and loss-of-function experiments, we investigated how an auditory go cue transforms thalamic and motor cortical activity patterns during movement initiation. Population responses in DN/IPN-recipient regions of motor thalamus were dominated by a time-locked increase in activity immediately prior to movement initiation, providing a fixed-latency feedforward timing signal to motor cortex. Consistent with this view, membrane potential dynamics of layer 5B projection neurons matched pre-movement timing of thalamic activation, while suppressing cerebellar or thalamic output blocked movement initiation. Conversely, photostimulation of DN/IPN or recipient thalamic regions triggered movement initiation, but in a context-dependent manner. Our results demonstrate an important and causal contribution of a cerebellar-thalamocortical pathway to voluntary movement initiation.

## Results

### Motor thalamic population activity increases prior to movement initiation

**Figure 1:**
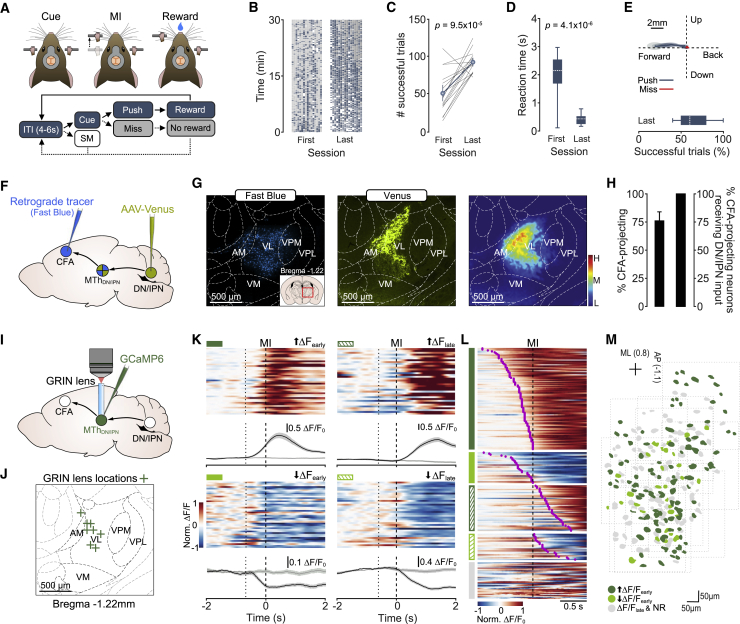
Increased activity dominates trial-to-trial MTh_DN/IPN_ population responses prior to movement initiation (A) Top: cued forelimb push task. MI, movement initiation. Bottom: behavioral task structure. ITI, inter-trial interval; SM, spontaneous movement. (B) Trial outcome rasters with each column representing individual mice (N = 16 mice). Blue, push trials; gray, miss trials; white, spontaneous movements. (C) Number of successful trials per 30-min behavioral session (N = 16 mice, two-sample t test). Mean ± 96% CIs. (D) Box-and-whisker plots showing mouse reaction times across learning (N = 16 mice, two-sample t test). (E) Top: forepaw trajectories for push (blue) and miss (red) trials from the mouse in Video S1. Thick line, average paw trajectory overlaid with 95% CI of paw position variance. Bottom: box-and-whisker plot of the percentage of successful trials in “expert” mice. (F) Mapping the dentate/interpositus thalamocortical pathway. CFA, caudal forelimb area; MTh_DN/IPN_, dentate/interpositus nuclei-recipient region of motor thalamus; DN, dentate nucleus; IPN, interpositus nucleus. (G) Left: retrograde labeling of CFA-projecting motor thalamic neurons. Middle: anterograde labeling of DN/IPN axons in motor thalamus. Right: density plot of regions of overlap of DN/IPN axons and CFA-projecting neurons across thalamic nuclei (N = 6 hemispheres from four mice). Inset: location of motor thalamic nuclei. AM, anteromedial; VL, ventrolateral; VPM, ventral posteromedial; VPL, ventral posterolateral; VM, ventromedial nuclei. (H) Left: percentage of CFA-projecting MTh_DN/IPN_ neurons. Right: percentage of CFA-projecting MTh_DN/IPN_ neurons receiving glutamatergic synaptic input from DN/IPN. Mean ± 95% CIs. (I) Two-photon population calcium imaging in MTh_DN/IPN_. (J) Locations of GRIN lenses in MTh_DN/IPN_ (N = 8 mice). (K) Activity of four example MTh_DN/IPN_ neurons. Clockwise from top left: early-onset increase (dark green), late-onset increase (dark green hatching), late-onset decrease (light green hatching), and early-onset decrease (light green). Top: normalized ΔF/F_0_ across successive trials. Bottom: ΔF/F_0_ mean ± SEM. Black lines, push trials; gray lines, miss trials; dotted lines, median cue onset; dashed lines, movement initiation (MI). (L) Average ΔF/F_0_ across trials for individual neurons. Groupings: early-onset increase (dark green, n = 104/248 neurons); early-onset decrease (light green, n = 32/248 neurons); late-onset increase (dark green hatching, n = 47/248 neurons); late-onset decrease (light green hatching, n = 27/248 neurons), and nonresponsive (gray, n = 38/248 neurons), ordered by ΔF/F_0_ onset, purple circles (n = 11 fields of view, N = 8 mice). (M) Spatial distribution of early-onset increase (dark green), early-onset decrease (light green) and late-onset/nonresponsive neurons (gray) in MTh_DN/IPN_. Dotted boxes, individual fields of view. ML, medial-lateral; AP anterior-posterior.

To investigate voluntary movement initiation, we developed a cued forelimb push task for mice. The design of the task required mice to execute horizontal push movements (4 mm) after a randomized inter-trial interval (4–6 s) and in response to a 6-kHz auditory go cue. Miss trials, partial pushes, or spontaneous lever movements resulted in no reward and a lever reset (Figure 1A; Video S1). Mice rapidly learned the task (mean = 7.5 days [6.3, 8.6] 95% confidence interval [CI], N = 16 mice; all data unless otherwise stated are presented as mean [bootstrapped 95% CI]; percentage of successful trials [last session], mean = 63.7% [56.0, 71.7]), displaying moderate reaction times (RTs; last session median = 0.32 s [0.30, 0.34]) and reproducible push trajectories (Figures 1B–1E; Video S1). Even in expert mice, we observed miss trials, likely reflecting changing levels of attention or satiation within sessions (Figure 1E; Video S1).

Video S1. Cued forelimb push task, related to Figure 1

Since both DN and IPN are implicated in motor timing and send glutamatergic projections to ventral motor thalamus (Aumann and Horne, 1996b; Bosch-Bouju et al., 2013; Gornati et al., 2018; Kuramoto et al., 2009), we sought to define the region of thalamus that receives input from DN/IPN and projects to the caudal forelimb area (CFA) of motor cortex using a dual labeling strategy (Figure 1F). A region of dense overlap centered on VAL and AM nuclei, with sparse colocalization in the ventral posteromedial nucleus (VPM). We found no overlap in the ventromedial nucleus (VM), which primarily receives input from the fastigial nucleus (Gao et al., 2018; Gornati et al., 2018) (Figures 1G and S1A–S1E). Although injections were targeted to DN/IPN, low-level expression was detected in some adjacent vestibular nuclei, which do not send direct projections to VAL (Figures S1A and S1C). Within the dense region of overlap, ∼76% of neurons project to CFA, and all CFA-projecting neurons received glutamatergic input (vesicular glutamate transporter 2 [VGluT2] positive) from DN/IPN (Bosch-Bouju et al., 2013; Kuramoto et al., 2009; Rovó et al., 2012; Schäfer et al., 2020) (Figures 1H and S2A–S2D). This degree of connectivity is consistent with DN/IPN-recipient regions of motor thalamus (MTh_DN/IPN_) being an important functional node connecting the cerebellum and CFA.

To explore whether MTh_DN/IPN_ population responses were consistent with a role in movement initiation, we employed gradient-index (GRIN) lens-mediated two-photon population calcium imaging (Figures 1I and 1J). Lens implantation above MTh_DN/IPN_ did not affect overall behavior when compared to control (control versus GRIN lens-implanted mice: two-sample Kolmogorov-Smirnov test, RT p = 0.56, push duration p = 0.22, number of successful pushes p = 0.35, N = 23 control versus 9 GRIN lens-implanted mice, data not shown). Most MTh_DN/IPN_ neurons displayed push-related activity (210/248 neurons) either prior to movement initiation (early-onset increase in ΔF/F_0_, 104/210 neurons; early-onset decrease in ΔF/F_0_, 32/210 neurons) or during the execution/reward period (late-onset increase in ΔF/F_0_, 47/210 neurons; late-onset decrease in ΔF/F_0_, 27/210 neurons), while during miss trials, MTh_DN/IPN_ population responses were absent (11 fields of view [FOVs], N = 8 mice) (Figures 1K–1L). Increased activity appeared as the dominant population response prior to movement (early-onset neurons: increased activity, 76.4%; decreased activity, 23.5%) (Figure 1L) and was found across the extent of MTh_DN/IPN_ (Figure 1M).

### MTh_DN/IPN_ output provides a reliable fixed-latency motor timing signal

**Figure 2:**
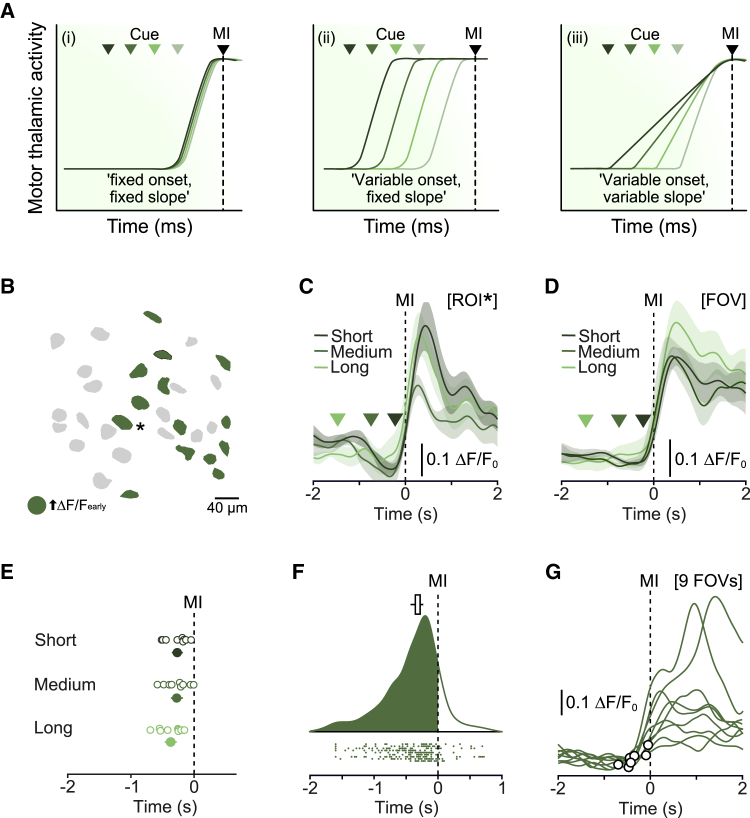
MTh_DN/IPN_ neurons provide a reliable time-locked signal prior to movement initiation (A) Trial-to-trial MTh_DN/IPN_ population response models. Green triangles, cue onset; MI, movement initiation. (B) Example field of view (FOV). Green, early-onset increase in activity. (C) Average ΔF/F_0_ from an early-onset increase neuron (asterisk in B), aligned to movement initiation (MI) and split by short, medium, and long reaction times. Colored triangles, median cue presentation; [ROI], region of interest. Error bars represent 95% CIs. (D) Average ΔF/F_0_ from all early-onset increase activity neurons in (B) FOV, aligned to movement initiation (MI) and split by reaction time. Triangles, median cue presentation. Error bars represent 95% CIs. (E) Mean onset times of trials split by reaction time. Open circles, individual FOVs; filled circles, means ± 95% CI (n = 9 fields of view, N = 6 mice). (F) Distribution of bootstrapped trial-to-trial response onsets for all early-onset increased activity neurons across nine FOVs. Top: median onset bootstrapped estimate. Middle: kernel density estimation of trial-to-trial motor thalamic response onsets. Bottom: raster of trial-to-trial population onsets (n = 297 trials from nine fields of view, N = 6 mice). (G) Single-trial ΔF/F_0_ population responses from nine different FOVs (one response per FOV). Black circles, population response onsets.

If MTh_DN/IPN_ conveys a motor timing signal, then population responses could be described by three hypothetical models. First, thalamic activity is uncoupled from the go cue rising immediately before movement onset. In this regard, rapidly increasing thalamic activity dictates the time of movement initiation (model i). Second, thalamic activity rises at the go cue and is maintained until additional inputs trigger movement. Thus, thalamic activity contributes to, but does not dictate, the time of initiation (model ii). Third, thalamic activity reflects a continuous sensorimotor transformation from cue to movement. The slope dictates the time of movement initiation (model iii) (Figure 2A). To distinguish between these models, we grouped trials by short, medium, and long RTs and aligned trial-averaged ΔF/F_0_ responses to movement initiation, focusing on early increased activity as this was the dominant population response (Figure 2B). Changes in ΔF/F_0_ occurred immediately prior to movement initiation, irrespective of RT (median onsets: short RT, −267 ms [−361, −178] 95% CI; medium RT, −276 ms [−374, −177] 95% CI; long RT, −367 ms [−464, −271] 95% CI, n = 104 neurons/9 FOVs, N = 6 mice, p = 0.46, one-way ANOVA). During medium and long RTs, baseline ΔF/F_0_ was maintained upon cue presentation, rising immediately before movement (Figures 2C and 2D). Response profiles were consistent trial to trial and across mice, indicative of a reliable motor timing signal that is temporally uncoupled from the auditory go cue (i.e., model i) (Figures 2E–2G).

### Early-onset activity in CFA correlates with MTh_DN/IPN_ response timing

**Figure 3:**
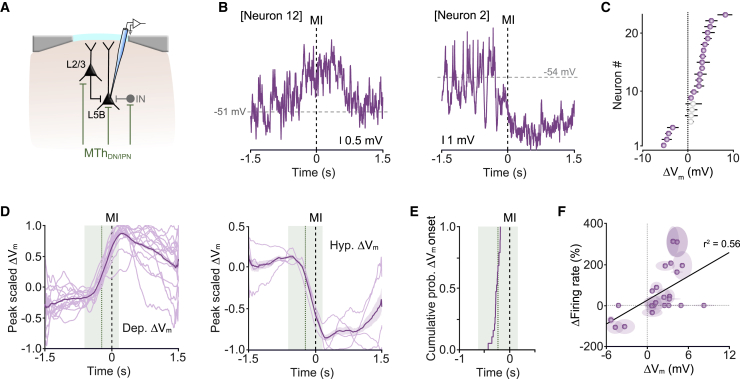
Early-onset changes in CFA layer 5B membrane potential dynamics (A) Patch-clamp recording in layer5B CFA. IN, interneuron; MTh_DN/IPN_, dentate/interpositus nucleus-recipient region of motor thalamus. (B) Single-trial subthreshold membrane potential (V_m_) trajectories from two layer 5B projection neurons (spikes clipped). MI, movement initiation. (C) Peri-movement ΔV_m_ ± 95% CI. Purple and white indicate significant and non-significant changes in ΔV_m_, respectively (n = 23 neurons from N = 23 mice). (D) Overlaid peak scaled subthreshold V_m_ split by direction of change (left: depolarizing, n = 15/23 neurons; right: hyperpolarizing, n = 4/23 neurons). Thick line, population mean ± 95% CI; green dotted line, mean MTh_DN/IPN_ activity onset ± 95% CI (green shading) shown in Figure 2E; black dashed line, movement initiation (MI). (E) Cumulative probability of ΔV_m_ onsets (n = 19/23 neurons). (F) Movement-related subthreshold ΔV_m_ and firing rate change correlation. Symbols, mean ± 95% CI from individual neurons; black line, linear fit to the data (Pearson’s *r*).

In rodents, projections from VAL thalamus target deep layers of motor cortex (Hooks et al., 2013; Kuramoto et al., 2009). This feedforward glutamatergic input provides monosynaptic excitation and disynaptic inhibition to CFA principal neurons (Apicella et al., 2012; Hooks et al., 2013), shaping cortical output and behavior (Hooks et al., 2013; Kuramoto et al., 2009; Sauerbrei et al., 2020; Schiemann et al., 2015; Tanaka et al., 2018) (Figure 3A). We reasoned that if the MTh_DN/IPN_ thalamocortical pathway conveys a pre-movement motor timing signal, then this should be reflected in the subthreshold membrane potential (V_m_) dynamics of CFA layer 5 pyramidal neurons. We confirmed that layer 5 neurons receive direct input from MTh_DN/IPN_ using monosynaptic retrograde rabies tracing in Rbp4-Cre transgenic mice (Gerfen et al., 2013; Kuramoto et al., 2009) (Figure S3A) before performing patch-clamp recordings (Figures 3A and S3B). When aligned to push onset, neurons displayed a rapid change in subthreshold activity, either depolarizing or hyperpolarizing, prior to movement initiation (depolarizing n = 15/23 neurons; hyperpolarizing, n = 4/23 neurons; nonresponsive, n = 4/23 neurons, N = 23 mice), with the direction of change being consistent trial to trial (Figures 3B and 3C). The timing of membrane potential changes (ΔV_m_) in layer 5B neurons closely matched MTh_DN/IPN_ population onsets (Figures 3D and 3E), consistent with direct feedforward modulation. Subthreshold V_m_ changes linearly correlated with firing rate in both intratelencephalic (IT-type) and pyramidal tract (PT-type) neurons that send projections to subcortical, brainstem, and spinal cord areas necessary for movement execution (Esposito et al., 2014; Kita and Kita, 2012; Park et al., 2021; Shepherd, 2013) (Figures 3F and S3C–S3J). During miss trials, ΔV_m_ was reduced, but not abolished, likely reflecting a lack of input from MTh_DN/IPN_ (see Figure 1K), but maintained behavior-related inputs from other brain areas (Hooks et al., 2013) (Figure S3K).

### Inactivating DN/IPN or MTh_DN/IPN_ blocks movement initiation

**Figure 4:**
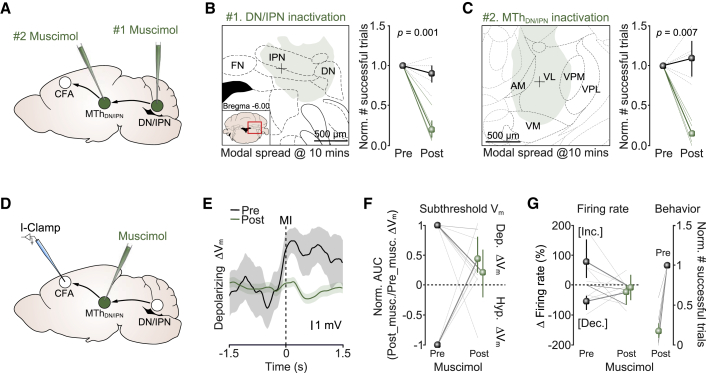
Activity in DN/IPN and MTh_DN/IPN_ is required for cue-triggered movement initiation (A) Muscimol inactivation of MTh_DN/IPN_ or DN/IPN. (B) Left: modal spread of fluorescent muscimol 10 min after injection into DN/IPN (inset, location of DN/IPN). Black cross, median point of IPN injection (N = 4 mice). Right: number of successful push trials 10 min after injection of saline (black, N = 6 mice) or muscimol (green, N = 6 mice ), two-sample t test. Symbols, population means ± 95% CI. FN, fastigial nucleus; IPN, interpositus nucleus; DN, dentate nucleus. (C) Left: modal spread of fluorescent muscimol 10 min after injection into MTh_DN/IPN_ (inset, location of thalamic nuclei). Black cross, median point of injection (N = 4 mice). Right: number of successful push trials 10 min after injection of saline (black, N = 5 mice) or muscimol (green, N = 5 mice), two-sample t test. Symbols, population means ± 95% CI. AM, anteromedial; VL, ventrolateral; VPM, ventral posteromedial; VPL, ventral posterolateral; VM, ventromedial nuclei. (D) Patch-clamp recording in layer 5B CFA during muscimol inactivation of MTh_DN/IPN_. I-Clamp, current clamp. (E) Subthreshold ΔV_m_ ± 95% CI from a layer 5B projection neuron before (Pre; black) and after muscimol injection (Post; green) targeted to MTh_DN/IPN_. (F) Ratio of normalized area under the curve for V_m_ trajectories before (Pre) and after (Post) muscimol injection into MTh_DN/IPN_. Data grouped by V_m_ change prior to muscimol injection. Green symbols, population means ± 95% CI (n = 10 cells from N = 10 mice). (G) Left: change in firing rate before (Pre) and after (Post) muscimol injection into MTh_DN/IPN_. Colored symbols, population means ± 95% CI; dotted lines, individual neurons (n = 8 cells from N = 8 mice); black lines, neurons with no change in firing rate prior to muscimol injection (n = 2 cells from N = 2 mice). Right: number of successful push trials 10 mins after injection of muscimol (green, N = 10 mice).

To test whether the DN/IPN thalamocortical pathway is necessary for movement initiation, we performed loss-of-function experiments by focally injecting a small bolus of the GABA_A_ receptor antagonist muscimol centered on DN/IPN, MTh_DN/IPN_, or CFA (Figures 4A and S4A–S4C). Injecting muscimol during task execution allowed the immediate effects to be recorded 10 min after injection, restricting diffusion beyond targeted regions. Mapping the spread of fluorescent muscimol (see STAR Methods) indicated limited spread (∼600 μm radius from the point of injection after 10 min) and localized inactivation of targeted nuclei (Figures 4B, 4C, and S4A–S4C). Our cortical injection strategy targeted all layers of CFA (spread diameter: anterior-posterior [AP], 1,240 ± 28.3 [SD] μm; mediolateral [ML], 1,133.2 ± 35.7 [SD] μm, N = 3 mice), without spreading to other cortical and subcortical areas (Figure S4A); similar results were found with DN/IPN injections (spread diameter: AP, 820 ± 89.4 [SD] μm; ML, 1,221.2 ± 265.4 [SD] μm, N = 4 mice) (Figure S4B). In ventral thalamus, spread was confined to MTh_DN/IPN_, with minimal overlap in VM (spread diameter: AP, 960 ± 73.5 [SD] μm; ML, 957.5 ± 34.9 [SD] μm, N = 4 mice) (Figure S4C). Mapping muscimol diffusion using silicon probe recordings *in vivo* further confirmed limited spread beyond 600 μm 10 min after injection (Figure S4D), consistent with previously published estimates (Allen et al., 2008; Krupa et al., 1999; Martin, 1991). Inactivation of each node along the DN/IPN thalamocortical pathway significantly reduced the number of successful push trials (normalized number successful trials post muscimol: DN/IPN, 0.20 [0.10, 0.34], p = 0.0013; MTh_DN/IPN_, 0.15 [0.05, 0.25], p = 0.007; CFA, 0.19 [0.08, 0.30] 95% CI, p = 0.025, N = 6, 5, and 5, respectively, two-sample t test; comparison of effect size across manipulations: p = 0.85, one-way ANOVA with Tukey-Kramer post hoc test) due to an increase in miss trials rather than incomplete lever pushes (Figures 4B, 4C, and S4E–S4G; Video S2). Miss trials did not result from task disengagement, as the go cue reproducibly evoked short-latency whisking and increased arousal (see Video S2). Silencing DN/IPN and CFA reduced paw position accuracy in some trials (i.e., the forepaw was not placed on the lever), indicative of a role in controlling posture and movement initiation, while inactivating MTh_DN/IPN_ selectively blocked movement initiation with no effect on paw placement accuracy (Figures S4E–S4G; Video S2).

Video S2. Muscimol inactivation of the CFA, MTh_DN/IPN_, and DN/IPN, related to Figure 4

To better understand how MTh_DN/IPN_ output shapes cortical activity and behavior, we performed patch-clamp recordings of CFA layer 5B projection neurons while inactivating MTh_DN/IPN_ (Figure 4D). Comparing the integral of subthreshold ΔV_m_ before and after silencing highlighted a reduction in ΔV_m_ magnitude irrespective of whether responses were depolarizing or hyperpolarizing. On average, neurons displaying depolarizing ΔV_m_ were reduced by ∼80% (normalized area under the curve [AUC] after muscimol, 0.22 [−0.27, 0.64] 95% CI, p = 0.03, two-sample t test, N = 6 mice), while hyperpolarizing responses switched polarity to become moderately depolarizing (normalized AUC after muscimol, 0.44 [0.17, 0.85] 95% CI, p = 0.006, two-sample t test, N = 4 mice) (Figures 4E and 4F). Residual ΔV_m_ changes likely reflect convergence of other long-range inputs conveying task-related information (Guo et al., 2018; Hooks et al., 2013) (see Figures 1K and S3K), which combine with MTh_DN/IPN_ input to trigger movement. As expected, blocking MTh_DN/IPN_ activity reduced layer 5B firing rate changes and the number of successful push trials (Figure 4G).

### Photoactivation of DN/IPN or MTh_DN/IPN_ mimics go cue-evoked movement initiation

**Figure 5:**
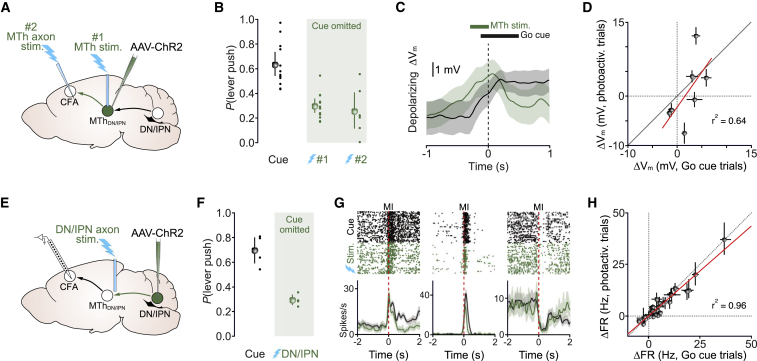
Stimulation of DN/IPN or MTh_DN/IPN_ axon terminals triggers movement initiation (A) Dual MTh photoactivation strategy; ChR2 expression targeted to MTh_DN/IPN_, stimulation via optic fiber directly above MTh_DN/IPN_ (#1) or tapered fiber in CFA (#2). (B) Full lever push probability during an auditory go cue (black) or photoactivation of MTh_DN/IPN_ (#1) or MTh_DN/IPN_ axons in CFA (#2) in the absence of a go cue (green). Colored dots, individual mice; colored circles, mean ± 95% CI (cue, 1 and 2; N = 12, 12, and 6 mice, respectively). (C) Change in subthreshold V_m_ ± 95% CI in a layer 5B projection neuron during the go cue (black) or photoactivation of MTh_DN/IPN_ (green) in the absence of a cue. Dashed line, movement initiation. (D) Peri-movement cue-evoked and photoactivated subthreshold ΔV_m_ correlation during push trials (n = 7 neurons, N = 6 mice). Filled symbols, mean ± 95% CI; red line, linear fit to the data (Pearson’s *r*). (E) Recording and photoactivation strategy: silicone probe recordings of deep-layer putative pyramidal cells in CFA during photoactivation of ChR2-expressing DN/IPN axon terminals in MTh_DN/IPN_. (F) Full lever push probability during an auditory go cue or photoactivation of DN/IPN axon terminals in MTh_DN/IPN_ in the absence of a go cue. Colored dots, individual mice; colored circles, mean ± 95% CI (N = 4 and 4 mice). (G) Spike rasters and peri-stimulus time histograms (PSTHs) from deep-layer CFA neurons aligned to movement initiation (dashed line). Black, cue trials; green, photoactivation trials. (H) Peri-movement cue-evoked and photoactivated Δfiring rate correlation during push trials (n = 30/47 neurons, N = 4 mice). Symbols, mean ± 95% CI; red line, linear fit to the data (Pearson’s *r*).

Although our loss-of-function experiments suggest the DN/IPN thalamocortical pathway is necessary for movement initiation, cerebellar and thalamic nuclei send projections to multiple brain regions involved in motor control (Asanuma et al., 1983; Hunnicutt et al., 2014; Kuramoto et al., 2009; Teune et al., 2000); therefore, we next tested whether stimulating MTh_DN/IPN_ input to CFA or cerebellar input to MTh_DN/IPN_ triggered movement. We reasoned that if the DN/IPN thalamocortical pathway conveys a movement timing signal, then photoactivation should mimic the effects of the go cue. To stimulate MTh_DN/IPN_, we injected AAV-ChR2 unilaterally, centered on MTh_DN/IPN_, chronically implanted an optic fiber directly above thalamus, and acutely inserted a tapered optic fiber directly into CFA (Figures 5A and S5A). ChR2 expression was restricted to the center of MTh_DN/IPN_ (i.e., VAL thalamic nuclei) with minimal off-target expression (Figures S5B and S5C). Direct stimulation of MTh_DN/IPN_ or axon terminals in CFA in the absence of an auditory cue-triggered full lever push movements in ∼30% of trials (go cue, lever push probability: *P*(lever push) 0.63 [0.53, 0.73] 95% CI; direct MTh_DN/IPN_ stimulation, *P*(lever push) 0.29 [0.24, 0.35] 95% CI; axon terminal stimulation, *P*(lever push) 0.25 [0.11, 0.40] 95% CI) and a small proportion of partial lever pushes (N = 12 mice) (Figures 5B, S5D, and S5E; Video S3). RTs and duration of photoactivated push movements were comparable to cue-evoked trials (Figures S5F and S5G), while stimulating in the absence of ChR2 expression did not evoke any detectable forelimb movements (go cue, *P*(lever push) 0.95 [0.89, 1.00] 95% CI; direct MTh_DN/IPN_ stimulation, *P*(lever push) 0.03 [0.00, 0.07] 95% CI, N = 2 mice) (data not shown). To compare cortical activity during go cue and photoactivation trials, we performed patch-clamp recordings from CFA layer 5B projection neurons. Go cue- and photoactivation-evoked ΔV_m_ were remarkably similar, both in the timing and direction of change, suggesting recruitment of the same inputs to CFA (Figures 5C, 5D, and S5H). Since stimulation of the ventral thalamus, including VM and VAL, has been shown to trigger short-latency licking (Catanese and Jaeger, 2021; Inagaki et al., 2020), we investigated whether MTh_DN/IPN_ acts as a convergence hub coordinating motor timing signals necessary for triggering both tongue and forelimb movements. However, photoactivation of MTh_DN/IPN_ rarely evoked short-latency licking or orofacial movements similar to those observed during a tactile delayed-response licking task (cue, *P*(lick): 0.77 [0.67, 0.87] 95% CI; photoactivation *P*(lick): 0.04 [0.00, 0.10] 95% CI, N = 12 mice, p = 4.1 × 10^−11^, two-sample t test) (Figure S5I) (Catanese and Jaeger, 2021; Guo et al., 2014; Inagaki et al., 2020). The selective triggering of forelimb push movements in our behavior suggests parallel but distinct thalamocortical pathways for tongue and limb movements.

Video S3. Photoactivation of MTh_DN/IPN_ axon terminals in CFA, related to Figure 5

We next investigated whether cerebellar input to MTh_DN/IPN_ can initiate movement by targeting ChR2 expression to DN/IPN and stimulating axons terminals in MTh_DN/IPN_ (Figures 5E and S5J–S5O). ChR2 expression was restricted to DN/IPN with minimal or no expression in surrounding nuclei (Figures S5J, S5K, and S5O). Photoactivation in the absence of the auditory go cue-triggered full lever push movements in ∼30% of trials (go cue, *P*(lever push) 0.70 [0.59, 0.80] 95% CI; DN/IPN axon terminal stimulation, *P*(lever push) 0.29 [0.25, 0.33] 95% CI, N = 4 mice), similar to direct MTh_DN/IPN_ stimulation (Figure 5F; Video S4). To investigate whether overlapping populations of CFA neurons were recruited during go cue and photoactivation trials, we used silicon probe recordings, focusing on a subset of deep-layer putative pyramidal neurons that were movement responsive (n = 47/216 neurons; N = 4 mice) (Figures S5P–S5U). Responses in individual neurons were highly consistent trial to trial, with movement-related activity patterns varying widely across the population (Figures 5G and S5U), consistent with our ground truth intracellular data (see Figures 3A–3C). A large proportion of cue-responsive neurons also displayed significant responses during photoactivation trials (30/47 neurons, 63.8%, from N = 4 mice), irrespective of the direction of firing rate change (Figures 5G, 5H, and S5U), suggesting photoactivation of the DN/IPN thalamocortical pathway mimics cue-evoked activity patterns in CFA.

Video S4. Photoactivation of DN/IPN axon terminals in MTh_DN/IPN_, related to Figure 5

### MTh_DN/IPN_ stimulation triggers behavioral context-dependent movement initiation

**Figure 6:**
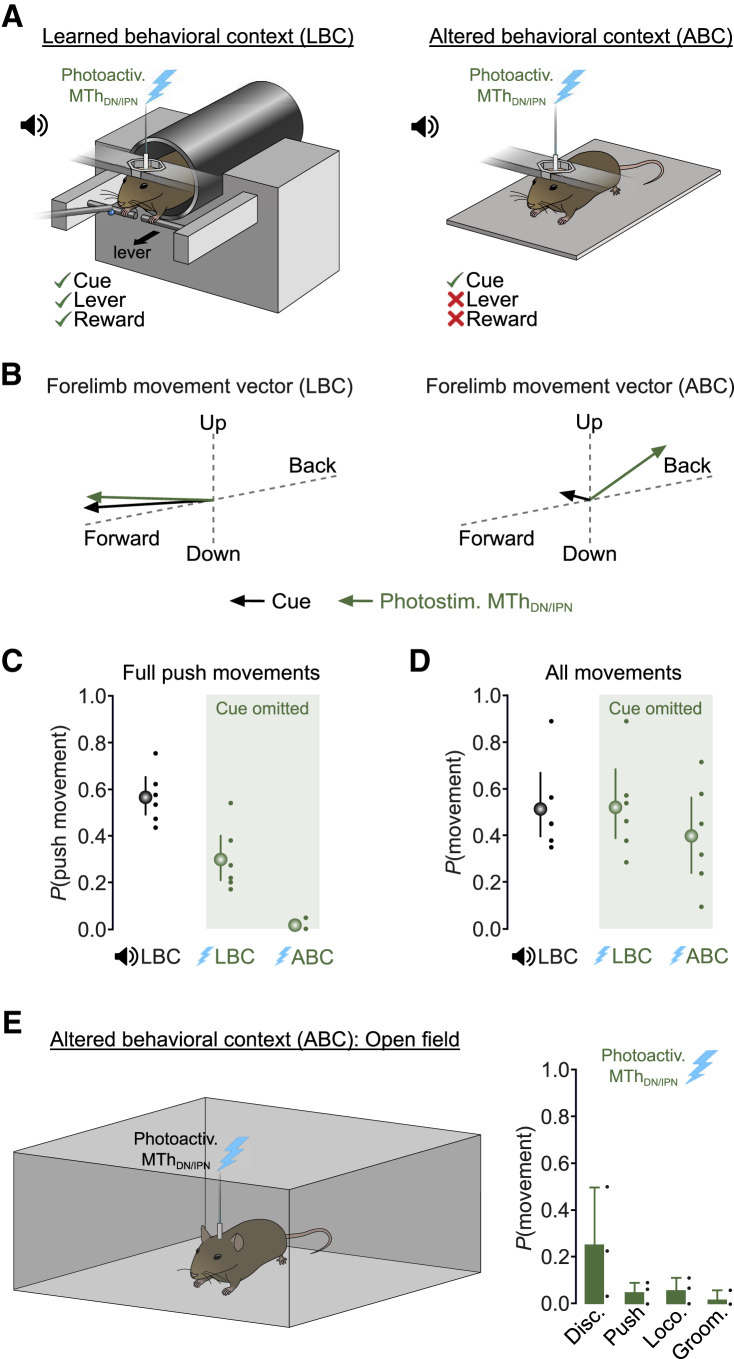
Photoactivation of MTh_DN/IPN_ evokes behavioral context-dependent movement initiation (A) MTh_DN/IPN_ photoactivation in a learned behavioral context (LBC; left) or altered behavioral context (ABC; right). (B) Average forelimb movement vectors from an example mouse during an auditory go cue (black arrows) or photoactivation of MTh_DN/IPN_ in the absence of a cue (green arrows). Arrow length, scaled by total number of across trial movements. (C) Push or push-like movement probability during an auditory go cue (black) or photoactivation of MTh_DN/IPN_ in the absence of a go cue (green). Dots, individual mice; symbols, mean ± 95% CI (N = 6 mice). (D) Forelimb movement probability during an auditory go cue (black) or photoactivation of MTh_DN/IPN_ in the absence of a go cue (green). Mean ± 95% CI (N = 6 mice). (E) Forelimb movement probability evoked by photoactivation of MTh_DN/IPN_ in an open-field environment (N = 3 mice). Disc., discrete movements; Push, push-like movements; Loco., locomotion; Groom, grooming behavior. Bars, mean ± 95% CI; dots, individual mice.

Presentation of a go cue or photoactivation of MTh_DN/IPN_ triggers lever pushes in a learned behavioral context (LBC). But whether push movements would be generated in an altered behavioral context (ABC) is unclear. If thalamocortical stimulation alone is sufficient to trigger the learned behavior, then photoactivation in an ABC should still generate “push-like” movements. However, if MTh_DN/IPN_ simply conveys a motor timing signal that combines with behavioral context-dependent inputs from other brain areas, then photoactivation of MTh_DN/IPN_ in an ABC will likely trigger movement, but not learned push movements. To address this, we designed an ABC, which consisted of a flat baseplate in the absence of any support/movable levers, reward spout, or water reward and compared cue- and MTh_DN/IPN_-photoactivation-evoked movements across contexts (LBC versus ABC) (Figure 6A). Mice were first trained in the LBC before being habituated to the ABC within and across training sessions to ensure cued lever push movements were not extinguished in the LBC. As expected, trained mice generated cue-evoked lever pushes in 56% of trials in the LBC but very few push movements in the ABC (LBC, *P*(push movement) = 0.56 [0.49, 0.65] 95% CI; ABC, *P*(push movement) = 0.01 [0.00, 0.03] 95% CI, p = 3.9 × 10^−7^, two-sample t test, N = 6 mice), confirming that mice acknowledged the difference between the two behavioral contexts (Figures 6B–6D and S6; Video S5). Direct photoactivation of MTh_DN/IPN_ in the LBC, and in the absence of a cue, evoked forelimb movements in 52% of trials, where 30% of trials were successful lever pushes (LBC, *P*(movement) = 0.52 [0.39, 0.69] 95% CI; LBC, *P*(push movement) = 0.30 [0.21, 0.41] 95% CI, N = 6 mice). While in the ABC, direct MTh_DN/IPN_ stimulation reliably evoked forelimb movements in 40% of trials but only 2% contained “push-like” movements (ABC, *P*(movement) = 0.40 [0.24, 0.56] 95% CI; ABC, *P*(push movement) = 0.02 [0.00, 0.03] 95% CI, p = 1.9 × 10^−3^, two-sample t test, N = 6 mice) (Figures 6B–6D and S6; Video S5). The absence of push-like movements could result from differences in posture; however, photostimulated movements in mice mounted on a flat baseplate (ABC) or in a behavioral context that recapitulated the LBC mouse posture (ABC2) were not different (Figure S6). In addition, photostimulation of MTh_DN/IPN_ in an open-field environment triggered discrete forelimb movements in ∼25% of trials, consistent with a role in movement initiation, but very few push-like movements (N = 3 mice) (Figure 6E). Together, these data suggest that the DN/IPN thalamocortical pathway conveys motor timing signals that trigger behavioral context-dependent movement initiation.

Video S5. Cue and photoactivation of MTh_DN/IPN_ (LBC and ABC), related to Figure 6

## Discussion

The cerebellum and motor thalamus are brain areas thought to control movement timing, since activity in both regions precedes movement initiation (Anderson and Turner, 1991; Butler et al., 1992, 1996; Fortier et al., 1989; Harvey et al., 1979; Horne and Porter, 1980; Kurata, 2005; Macpherson et al., 1980; Mushiake and Strick, 1993; Schmied et al., 1979; Strick, 1976; van Donkelaar et al., 1999) and local inactivation disrupts motor timing (Meyer-Lohmann et al., 1977; Nashef et al., 2019; Spidalieri et al., 1983; Thach, 1975; van Donkelaar et al., 2000). Our anatomical mapping identified a high degree of connectivity between DN/IPN and CFA-projecting neurons in VAL, AM, and VPM. In rodents, VAL neurons receive strong driver-like inputs (Gornati et al., 2018) that facilitate rapid depolarization of thalamic projection neurons (Aumann and Horne, 1996a, 1996b; Aumann et al., 1994; Gao et al., 2018; Gornati et al., 2018; Sawyer et al., 1994; Schäfer et al., 2021). This driving input, when integrated with GABAergic input from the basal ganglia and thalamic reticular nucleus, shapes the magnitude and timing of thalamic excitability (Bosch-Bouju et al., 2013; Catanese and Jaeger, 2021; Kim et al., 2017; Kuramoto et al., 2011; Lam and Sherman, 2015; Sakai et al., 1998; Tanaka et al., 2018). Early-onset MTh_DN/IPN_ activity was temporally uncoupled from the go cue but tightly locked to movement initiation, suggestive of a pure motor timing signal that indicates the intention to move rather than a sensorimotor transformation from cue to movement (see Figure 2). Consistent with this view, cue presentation during miss trials did not evoke a change in activity, likely reflecting a lack of intention to move and absence of direct auditory input in VAL thalamus, while suppressing MTh_DN/IPN_ generated a selective block of forelimb movement initiation.

Although we focused on the DN/IPN thalamocortical pathway, projections from DN/IPN also target the ventral tegmental area, substantia nigra reticulata, brainstem reticular nucleus, and magnocellular red nucleus (Carta et al., 2019; Gornati et al., 2018; Houck and Person, 2015; Low et al., 2018; Sakayori et al., 2019; Sathyamurthy et al., 2020; Thanawalla et al., 2020). Direct projections to the brainstem provide an alternate pathway to initiate movement. We found that recruitment of the DN/IPN thalamocortical pathway is necessary for learned forelimb movement initiation given that photoactivation of DN/IPN axon terminals in MTh_DN/IPN_ mimics cue-triggered CFA population dynamics and behavior, while silencing each node along the pathway blocked initiation. These observations differ from photomodulation of cerebellar output in locomoting mice, where stimulation initiates or modifies sequences of limb movements via descending projections to the brainstem (Hoogland et al., 2015; Jelitai et al., 2016; Witter et al., 2013). Direct photoactivation of MTh_DN/IPN_ in an open-field environment triggered discrete, but not rhythmic, forelimb movements, suggesting selective recruitment of descending or thalamocortical pathways depending on movement type and behavioral context. In addition to a proposed role in movement initiation, DN/IPN contribute to the coordination of ongoing movements. IPN inactivation results in disrupted endpoint accuracy, hypermetria, and instability of the forelimb (Becker and Person, 2019; Bracha et al., 1999; Low et al., 2018; Martin et al., 2000; Mason et al., 1998), while DN inactivation increases path curvature and generates hypermetria and a general impairment in coordination (Ishikawa et al., 2014; Martin et al., 2000). The fact that DN/IPN inactivation reduced both paw position accuracy (i.e., the ability to maintain postural control) and movement initiation is consistent with a role in both motor timing and coordination.

Changes in MTh_DN/IPN_ and CFA layer 5B neuron activity occurred prior to movement and peaked around movement completion, indicative of rapid preparatory activity that transforms into output dynamics necessary for execution (Lara et al., 2018). In rodents, rapid go cue-evoked changes in activity have been observed in a delayed directional licking task for mice (Catanese and Jaeger, 2021; Gao et al., 2018; Guo et al., 2014; Li et al., 2015), where input from the pedunculopontine nucleus, midbrain reticular nucleus, and substantia nigra reticulata, via ventral motor thalamus, triggers rapid reorganization of preparatory dynamics to initiate directional licking (Catanese and Jaeger, 2021; Inagaki et al., 2020). Thus, ventral thalamus appears ideally positioned to act as a central convergence hub, integrating input from the cerebellum, brainstem, and basal ganglia in order to initiate precisely timed movements. However, direct photoactivation of MTh_DN/IPN_ did not reproducibly evoke short-latency tongue or orofacial movements, suggesting parallel, non-overlapping thalamocortical pathways for movement initiation. Directional licking requires channeling of information through VM, VAL, mediodorsal, and intralaminar nuclei for both movement planning and execution (Catanese and Jaeger, 2021; Inagaki et al., 2020), while forelimb movements require activity in VAL, AM, and VPM nuclei. Together, this suggests that parallel processing of motor timing signals through different ventral motor thalamic nuclei could provide an anatomical substrate for initiating complex, multifaceted motor behaviors.

Using monosynaptic rabies tracing, we confirmed a direct pathway from MTh_DN/IPN_ to layer 5B projection neurons in CFA, consistent with the idea that VAL projects to both superficial and deep layers of motor cortex, while neurons in VM project primarily to L1 (Hooks et al., 2013; Kuramoto et al., 2009, 2015). Strong thalamic input generates monosynaptic excitation and disynaptic feedforward inhibition in principal neurons (Apicella et al., 2012; Hooks et al., 2013), shaping cortical output via top-down control or direct activation of output layers (Hooks et al., 2013; Sauerbrei et al., 2020; Weiler et al., 2008). Since photoactivation of the MTh_DN/IPN_ thalamocortical pathway reproduced go-cue-evoked layer 5B neuronal dynamics, thalamic input may directly influence cortical output by bypassing top-down processing from layer 2/3 to inform PT-type and IT-type neurons of the intention to move (Hooks et al., 2013; Weiler et al., 2008). This direct timing signal would be integrated with other long-range task-relevant inputs to generate specific output patterns necessary for forelimb motor control (Esposito et al., 2014; Kita and Kita, 2012; Park et al., 2021; Sauerbrei et al., 2020). Although we focused solely on the contribution of CFA, VAL also sends projections to the rostral forelimb area (RFA) (Hooks et al., 2013; Oh et al., 2014), which plays an integral role in movement coordination (Brown and Teskey, 2014; Morandell and Huber, 2017). Given its strong reciprocal connectivity with CFA (Hira et al., 2013; Mohammed and Jain, 2016; Rouiller et al., 1993), assessing the contribution of the VAL-RFA pathway to movement initiation will be an important next step.

Our behavioral context experiments further support MTh_DN/IPN_ conveying a generic motor timing signal that converges, at the level of motor cortex, with other task-relevant inputs. In the absence of thalamic input to MTh_DN/IPN_ (i.e., miss trials or MTh inactivation), layer 5B V_m_ and firing rate changes were significantly reduced, with residual V_m_ changes being insufficient to trigger movement, suggesting input convergence is a prerequisite for learned movement initiation. The origin of the additional input(s) remains unknown, but likely candidates are cortico-cortical interactions between frontal motor areas and CFA (Hooks et al., 2013; Reep et al., 1990), thought to accumulate task-relevant information required for motor planning and execution (Gao et al., 2018; Li et al., 2015), or basal ganglia thalamocortical interactions involved in selecting, timing, and invigorating different actions (Dudman and Krakauer, 2016; Inase et al., 1996; Klaus et al., 2019; Thura and Cisek, 2017; Williams and Herberg, 1987). Directly activating the MTh_DN/IPN_ thalamocortical pathway in the LBC mimicked the go cue by triggering push movements, while in the ABC, photoactivation evoked highly variable forelimb trajectories. Why does photoactivation result in learned movement initiation in the absence of an external sensory cue? We suggest that the DN/IPN thalamocortical pathway provides one of the main driving inputs to CFA, which combines with other task-relevant inputs (e.g., behavioral context, stimulus-reward associations, and reward expectancy) to generate “learned” cortical output patterns and behavior. In the ABC, task-relevant inputs are likely absent, thus mimicking the thalamic “timing signal” is in itself sufficient to generate cortical output patterns necessary for movement (Tanaka et al., 2018), but not the learned movement.

In summary, our findings extend our understanding of how specific subdivisions of the motor thalamus contribute to motor timing, suggesting that the DN/IPN thalamocortical pathway plays a critical role in generating cortical dynamics necessary for context-dependent movement initiation.

## STAR★Methods

### Key resources table

**Table:** 

REAGENT or RESOURCE	SOURCE	IDENTIFIER
**Antibodies**		

Mouse monoclonal anti-Satb2	Thermo Fisher Scientific	Abcam Cat# ab51502; RRID:AB_882455
Rat monoclonal anti-Ctip2	Thermo Fisher Scientific	Abcam Cat# ab18465; RRID: AB_2064130
Anti-VGluT2 Antibody	Sigma-Aldrich	Cat# AB2251-I; RRID:AB_2665454
Cy5 AffiniPure donkey anti-guinea pig	Jackson ImmunoResearch Europe Ltd.	Cat# AB2251-I; RRID:AB_2340462
AlexaFluor-568 goat anti-mouse	Molecular Probes	Cat#: A- 21124; RRID: AB_2535766
AlexaFluor-647 goat anti-rat	Molecular Probes	Cat#: A-21247; RRID: AB_141778

**Bacterial and virus strains**		

AAV2/1-CAG-FLEX-mTagBFP2-2A-TVA	Kimberly Ritola at Janelia Farm-Molecular Biology Shared Resource; Huang et al., 2013	N/A
Pseudotyped SADΔG-mCherry(EnvA) rabies	Salk Institute Vector Core; Wickersham et al., 2007	N/A
AAV2/1-CAG-EGFP	Penn Vector Core	Addgene 28014
AAV1-Syn-GCaMP6s	Penn Vector Core; Chen et al., 2013	Addgene 100844
AAV1-CAG-ChR2-Venus	UNC Vector Core; Addgene; Petreanu et al., 2009	Addgene 20071
AAV2-CAG-mCherry	Homemade: McClure at Edinburgh	Addgene 108685

**Chemicals, peptides, and recombinant proteins**		

Muscimol hydrobromide	Sigma-Aldrich, Missouri, USA	Cat#: G019-5MG
Muscimol, BODIPY TMR-X Conjugate	Thermo Fisher Scientific	Cat#: M23400
Fast Blue	Polysciences	Cat#: 17740
Red Retrobeads™	Lumaflor	N/A
Green Retrobeads™	Lumaflor	N/A
Vybrant DiI Cell-Labeling Solution	Thermo Fisher Scientific	Cat#: V22885
NeuroTrace Blue Fluorescent Nissl Stain	Thermo Fisher Scientific	Cat#: N21479
Cholera Toxin Subunit B (Recombinant), Alexa Fluor 647	Thermo Fisher Scientific	Cat#: C34778

**Deposited data**		

Raw and analyzed data	This paper	N/A

**Experimental models: organisms/strains**		

Mouse: C57BL/6J	The Jackson Laboratory	RRID: IMSR_JAX:000664
Mouse: Rbp4-Cre: Tg(Rbp4-cre)KL100Gsat	The Jackson Laboratory	RRID:MMRRC_031125-UCD

**Software and algorithms**		

MATLAB	MathWorks (https://www.mathworks.com/)	RRID: SCR_001622
Python 3	Python (https://www.python.org/)	RRID: SCR_008394
AxonpCLAMP 10	Molecular Devices (https://www.moleculardevices.com/)	RRID:SCR_011323
Streampix 7.0	Norpix (https://www.norpix.com/products/streampix/streampix.php)	RRID:SCR_015773
NIS-Elements	Nikon (https://www.microscope.healthcare.nikon.com/products/software)	RRID:SCR_014329
Arduino IDE 1.6.5	Arduino (https://www.arduino.cc/en/software)	N/A
SpikeGLX	Jun et al., 2017 (http://billkarsh.github.io/SpikeGLX/)	N/A
Mantis64	https://www.mantis64.com/	N/A
Kilosort3	https://github.com/MouseLand/Kilosort	N/A
Phy	Jun et al., 2017 (https://github.com/cortex-lab/phy)	N/A
FIJI	Schindelin et al., 2012*(*https://github.com/fiji*)*	N/A
Deeplabcut	Adaptive Motor Control Lab (https://github.com/DeepLabCut/DeepLabCut)	N/A
NoRMCorre	Pnevmatikakis and Giovannucci, 2017 (https://github.com/flatironinstitute/NoRMCorre)	N/A
nmf_sklearn	Keemink et al., 2018 (https://github.com/rochefort-lab/fissa)	N/A
Onset detection algorithm	Zong et al., 2003	N/A

**Data acquisition**		

Data acquisition system	Molecular Devices	Digidata 1440A
Amplifier	Molecular Devices	Multiclamp 700B
Neuropixel probes	Janelia/IMEC	Phase 3B
Gradient-index (GRIN) lens	Grintech	Cat#: NEM-060-15-15-520-S-1.0p
Laser, Ti:Sapphire pulsed	Coherent	Chameleon Vision-S

**Other**		

Fiber optic cannula	ThorLabs	Cat#: CFMLC12L05
Optogenix tapered optic fiber cannula	Plexon	LambdaB (0,39, 200, 1.5, 225, 1.25)
Optic fiber patch cable		N/A
Laser, 473 nm	Civillaser	473nm 100 mW Blue DPSS Laser with Power Supply
Shutter	Uniblitz	Cat#: LS3S2T1
Arduino UNO	Arduino (https://www.arduino.cc/en/Guide/ArduinoUno/)	RRID:SCR_017284

### Resource availability

#### Lead contact

Further information and requests for resources and reagents should be directed to and will be fulfilled by the lead contact, Ian Duguid (ian.duguid@ed.ac.uk).

#### Materials availability

This study did not generate new unique reagents.

#### Data and code availability

Data analyzed and code generated in this study are available upon written request to corresponding author.

### Experimental model and subject details

All experiments and procedures were approved by the University of Edinburgh local ethical review committee and performed under license from the UK Home Office in accordance with the Animal (Scientific Procedures) Act 1986. Male adult C57BL/6J wild-type (RRID: IMSR_JAX:000664) and Rbp4-Cre (RRID:MMRRC_031125-UCD) mice (5-14 weeks old, 20-30 g, one to six animals per cage) were maintained on a reversed 12:12 hour light:dark cycle (lights off at 7:00 am) and provided *ad libitum* access to food and water except during behavioral training and experimentation (see below).

### Method details

#### General surgery

Mice undergoing surgery were induced under 4% and maintained under ∼1.5% isoflurane anesthesia, with each animal receiving fluid replacement therapy (0.5 ml sterile Ringer’s solution; to maintain fluid balance) and buprenorphine (0.5 mg/kg; for pain relief) at the beginning of each surgery. Additionally, buprenorphine (0.5 mg/kg) was administered in the form of an edible jelly cube ∼24 hours after recovery from surgery. For surgeries involving removal of the periosteum, each animal received an injection of carprofen (5 mg/kg). A small lightweight headplate (0.75 g) was implanted on the surface of the skull using cyanoacrylate super glue and dental cement (Lang Dental, USA) and mice were left for 24-48 hours to recover. Craniotomies were performed in a stereotactic frame (Kopf, USA) using a hand-held dentist drill with 0.5 mm burr (craniotomy diameter: whole-cell patch-clamp recording ∼⌀300 μm; viral / tracer / pharmacological compound injection ⌀500-1000 μm). Viral vectors and tracing compounds were delivered via pulled glass pipettes (5 μl, Drummond) using an automated injection system (Model Picospritzer iii, Intracell). At the end of each experiment, mice were anesthetized with euthatal (0.10–0.15 ml) and transcardially perfused with 30 mL of ice-cold 0.1 M phosphate-buffered saline (PBS) followed by 30 mL of 4% paraformaldehyde (PFA) in 0.1 M PBS solution. Brains were post-fixed in PFA overnight at 4°C then transferred to 10% sucrose solution for longer-term storage.

#### Behavioral training

Mice were handled extensively before being head restrained and habituated to the behavioral setup. To increase task engagement, mice were placed on a water control paradigm (1 ml/day) and weighed daily to ensure body weight remained above 85% of baseline. Mice were trained for one 30-minute session per day, during which they had to hold a moveable lever still during a random inter-trial-interval (ITI) of 4-6 s, before pushing the lever 4 mm during presentation of a 6 KHz auditory ‘go cue’ to receive a 5 μl water reward. The duration of the auditory cue (and thus response period) was reduced across training sessions in three stages: stage 1) 10 s, stage 2) 5 s, stage 3) 2 s, with mice advancing to the next stage after achieving > 80 rewards during a single session or > 50 rewards during two consecutive sessions. Mice were deemed “expert” after achieving > 80 rewards on two consecutive days of stage 3 training. Lever movements during the ITI would result in a lever reset and commencement of a subsequent ITI. After each 30-minute session, mice were removed from head restraint and given the remainder of their daily water allowance before being returned to their home cage.

#### *In vivo* pharmacology

To assess the behavioral effects of CFA, MTh_DN/IPN_ or DN/IPN inactivation, a craniotomy was performed above the target area under general anesthesia. After 5/10 minutes of baseline task execution, the lever was locked and a small volume of the GABA_A_ receptor agonist muscimol (dissolved in external solution containing 150 mM NaCl, 2.5 mM KCl, 10 mM HEPES, 1.5 mM CaCl_2_ and 1 mM MgCl_2_) or saline was injected into the target area (CFA: 200 nl of 2 mM muscimol at each of 5 sites centered on AP: 0.6, ML: 1.6, DV: −0.7 mm; MTh_DN/IPN_: 200 nl of 1 mM muscimol, AP: −1.1, ML: 1.0, DV: −3.4 mm; ipsilateral DN: 100 nl of 1 mM muscimol, AP: −6.0, ML: −2.25, DV: −2.4 mm; ipsilateral IPN: 50nl of 1 mM muscimol, AP: −6.0, ML: −1.75, DV: −2.4 mm) at a rate of 5-20 nl/s. To confirm the anatomical location of drug injection, 1% w/v of retrobeads (Lumaflor Inc.) was included in the muscimol/saline solution. Mice were randomly assigned to drug or control groups, and experiments performed blinded. After each experiment, mice were transcardially perfused and coronal sections (60 μm) of CFA, MTh_DN/IPN_ or DN/IPN were cut with a vibratome (Leica VT1000S), mounted with Vectashield mounting medium (H-1000, Vector Laboratories), imaged using a fluorescence microscope (Leica DMR, 5x objective) and manually referenced to the Paxinos and Franklin Mouse Brain Atlas (Paxinos and Franklin, 2008). Behavioral metrics were analyzed in 5-minute epochs using custom-written MATLAB (MathWorks) scripts, a two-sample t test was used to compare experimental cohorts during the first post injection epoch, and a one-way ANOVA was used to compare data between manipulation experiments. Behavioral video data for all pharmacology experiments was captured using a high speed camera (Genie HM640, Dalsa), and paw position accuracy was calculated as the proportion of trials in which mice were holding the moveable lever with their contralateral forepaw at the onset of the auditory cue.

#### GRIN lens imaging

To perform population calcium imaging in motor thalamus, 200 nl of AAV1-Syn-GCaMP6s was injected into contralateral MTh_DN/IPN_ (AP: − 1.1, ML: 1.0, DV: − 3.4 mm) and mice were implanted with a lightweight headplate. 7-10 days after virus injection, a gradient-index (GRIN) lens (Grintech NEM-060-15-15-520-S-1.0p; 600 μm diameter, 4.83 mm length, 0.5 numerical aperture) was implanted as described previously (Xu et al., 2016). In brief, a sterile needle (1.1 mm OD) surrounding the GRIN lens was lowered to a depth of 3.2 mm and subsequently retracted leaving the lens at the desired depth. The lens was then secured in place with UV curing glue (Norland Products, USA) and dental cement. Fields of view were checked for clarity and GCaMP6s expression after implantation. After 4-8 weeks mice began water restriction and behavioral training. Two-photon calcium imaging was performed in expert mice during lever task execution with a 318 × 318 μm field of view (600 × 600 pixels) at 40 Hz frame rate, using a Ti:Sapphire pulsed laser (Chameleon Vision-S, Coherent, CA, USA; < 70 fs pulse width, 80 MHz repetition rate) tuned to 920 nm wavelength with a 40x objective lens. For confirmation of GRIN lens location and viral expression, mice were perfused, sagittal sections (100 μm) of MTh_DN/IPN_ were cut with a vibratome, counterstained with Nissl blue, and imaged using a slide scanner (Axioscan, Zeiss). GRIN lens location was referenced to the Paxinos and Franklin Mouse Brain atlas.

Raw imaging videos were motion corrected using NoRMCorre (Pnevmatikakis and Giovannucci, 2017). In brief, NoRMCorre performs non-rigid motion correction by splitting each FOV into overlapping patches, estimating the xy translation for each patch, and upsampling the patches to create a smooth motion field, correcting for non-uniform motion artifacts caused by raster scanning or brain movement. Regions of interest (ROIs, polygonal areas) were drawn manually using Fiji (Schindelin et al., 2012). Signals were extracted and neuropil decontaminated using nmf_sklearn (Keemink et al., 2018). Normalized signal was calculated as ΔF/F_0_, where F_0_ was calculated as the bottom 5th percentile of the 1Hz low pass filtered raw signal, and ΔF = (F-F_0_). Normalized signals were then aligned to the behavioral data and analyzed using custom-written MATLAB scripts.

To detect activity changes of cells, a Friedman test was used to compare 250 ms time binned Ca^2+^ signals from 500 ms before movement to 1500 ms after movement with a significance threshold of p < 0.01. A Tukey-Kramer post hoc test was used to identify significantly different bins, and the direction of each response was defined based on the difference between baseline and the mean value of the Ca^2+^ signal in the earliest two significantly different bins. The median onset time of each cell was calculated by employing a previously published onset detection algorithm using a slope sum function (SSF; Zong et al., 2003) with the decision rule and window of the SSF adapted to the calcium imaging data (threshold 10% of peak, SSF window 375 ms, smoothed with a Savitzky Golay filter across 27 frames with order 2) and reported as the median of 10,000 bootstrapped samples to reduce the influence of noisy individual trials. Neurons whose bootstrapped samples had inter-quartile ranges exceeding 3 standard deviations of the median inter-quartile range were considered to have undetectable onset times and categorized as non-responsive. Prior to extracting ΔF/F_0_ onsets, we verified this algorithm with simulated data thereby accounting for any bias in the onset detection potentially introduced by filtering and/or the decision rule. To simulate the rising phase of the movement related calcium events in our data we used linear ramps with defined onset times and a rise time of 0.5 s mimicking GCaMP6s kinetics. We then calibrated the onset detection algorithm on the simulated data (100 simulated cells with 30 simulated trials per cell and artificially added noise in each trial matching the noise level in the imaging data) and updated it by a small FOV-specific correction factor.

Onset times were used to classify MTh_DN/IPN_ neurons as preceding movement initiation (early) from those occurring after movement initiation (late). To investigate the relationship between ΔF/F_0_ trajectories and reaction time, reaction times were split into thirds (short [0 – 350 ms], medium [350 - 900 ms] and long [> 900 ms]) and only FOVs with a sufficient number of trials per reaction time category were included in further analysis. To compare the onset times across short, medium and long reaction time trials, the onset time of each neuron was calculated using only these subsets of trials. Movement-aligned time binned Ca^2+^ signals were presented smoothed with the loess method using a 40-frame sliding window and baseline corrected to the mean of the 500 ms pre-cue period. A kernel density estimate was calculated for each onset across all trials to calculate a mean. The area under the mean population kernel density estimate was calculated using numerical trapezoidal integration.

To investigate whether GRIN lens implantation surgery affected lever task execution, a two-sample Kolmogorov-Smirnov goodness-of-fit test was used to compare reaction time, push duration and task success (the ratio of the number of rewarded trials to total number of cued trials) of the GRIN lens-implanted cohort and a control group.

#### Whole-cell patch-clamp electrophysiology

Whole-cell patch-clamp recordings targeted to layer 5B, 550–1000 μm from the pial surface, were obtained from awake head restrained mice after performing a craniotomy and durotomy centered above CFA. Signals were acquired at 20 kHz using a Multiclamp 700B amplifier (Molecular Devices) and filtered at 10 kHz using PClamp 10 software in conjunction with a DigiData 1440 DAC interface (Molecular Devices). No bias current was injected during recordings and the membrane potential was not corrected for junction potential. Resting membrane potentials were recorded immediately after attaining the whole-cell configuration (break-in). Series resistances (Rs) ranged from 23.6 to 45.5 MΩ. Patch pipettes (5.5–7.5 MΩ) were filled with internal solution (285–295 mOsm) containing: 135 mM K-gluconate, 4 mM KCl, 10 mM HEPES, 10 mM sodium phosphocreatine, 2 mM MgATP, 2 mM Na_2_ATP, 0.5 mM Na_2_GTP, and 2 mg/ml biocytin (pH adjusted to 7.2 with KOH). External bath solution contained: 150 mM NaCl, 2.5 mM KCl, 10 mM HEPES, 1 mM CaCl_2_, and 1 mM MgCl_2_ (adjusted to pH 7.3 with NaOH). All electrophysiology recordings were analyzed using custom-written scripts in MATLAB. Individual action potentials (APs) were detected with a wavelet-based algorithm (Nenadic and Burdick, 2005) and AP threshold was defined as the membrane potential (V_m_) at maximal d^2^V/dt^2^ up to 3 ms before AP peak and manually verified. For subthreshold V_m_ analysis APs were clipped by removing data points between −1 and +9 ms peri-AP threshold. Average AP firing frequencies were calculated by convolving spike times with a 50 ms Gaussian kernel. Significant changes in subthreshold V_m_ and AP firing frequency were defined by comparing bootstrapped 95% confidence intervals of mean movement-aligned V_m_ and AP frequency trajectories to zero (baseline epoch = 200 ms pre-cue; movement epoch = −100 to +100 ms peri-movement). Mean ΔV_m_ trajectories were calculated by subtracting the mean V_m_ during baseline (1 s epoch prior to cue) from the mean V_m_ during the peri-movement epoch (−250 to +250 ms when aligned to movement onset). All mean ΔV_m_ trajectories were decimated and median filtered with a 50 ms sliding window. Population mean ΔV_m_ trajectories were normalized to the largest absolute mean ΔV_m_ value in a 1.5 s peri-movement window. Peri-movement ΔV_m_ onsets were detected as the 10% rise-time of V_m_ trajectories when aligned to movement.

To inactivate motor thalamus during patch clamp recordings we performed a second craniotomy above MTh_DN/IPN_ and a pipette containing 1 mM muscimol (dissolved in external solution) and 1% w/v red retrobeads was targeted to MTh_DN/IPN_ (AP: −1.1, ML: 1.0, DV: −3.4 mm). Once the whole-cell recording configuration had been obtained, 5-10 minutes of baseline behavior and electrophysiological data were acquired before 200 nL of muscimol was injected at a rate of 5-10 nl/s. Mice were perfused, and data analyzed from animals in which retrobeads were found within MTh_DN/IPN_. To compare subthreshold V_m_ dynamics during pre- and post- injection epochs, cue-aligned periods of V_m_ were baseline subtracted and the area under the |ΔV_m_| trajectory from cue onset to median reward delivery was calculated via trapezoidal numerical integration with a 50 ms sample rate. To compare firing rate dynamics, the proportional difference between peri-median reaction time versus baseline Gaussian kernel smoothed firing rate was calculated in both epochs using bin sizes described previously.

#### Immunohistochemistry

To morphologically identify neurons after whole-cell patch-clamp recording, mice were transcardially perfused and coronal sections (60 μm) of CFA were cut with a vibratome. To recover neurons, sections were incubated in streptavidin AlexaFluor-488 (1:1000, Molecular Probes) in 0.1 M PBS containing 0.5%Triton X-100, mounted, imaged using a confocal microscope (Zeiss LSM800, 20x objective) and referenced to the Paxinos and Franklin Mouse Brain Atlas. To identify projection targets of individually recorded neurons (Schiemann et al., 2015), sections were further processed by heat-mediated antigen retrieval in 10 mM sodium citrate buffer (pH 6.0) for 3 hours at 80°C. Sections were incubated in blocking solution (0.01 M PBS, 10% normal goat serum (NGS), 0.5% Triton X-100) at 22°C for 2 hours and incubated overnight at 22°C in a primary antibody mixture containing mouse monoclonal anti-Satb2 (1:200, Cat. No. ab51502, Abcam) and rat monoclonal anti-Ctip2 (1:1000, Cat. No. ab18465, Abcam) dissolved in carrier solution (0.01 M PBS, 1% NGS, 0.5% Triton X-100). Slices were then incubated overnight at 22°C in a secondary antibody mixture containing AlexaFluor-568 goat anti-mouse (1:750, Molecular Probes) and AlexaFluor-647 goat anti-rat (1:750, Molecular Probes) dissolved in carrier solution (0.01 M PBS, 1% NGS, 0.5% Triton X-100), mounted and imaged using a confocal microscope (Zeiss LSM800, 20x objective).

To assess the proportion of CFA-projecting MTh_DN/IPN_ neurons that receive glutamatergic synaptic input from dentate/interpositus nuclei, selected motor thalamic coronal sections (60 μm) were rinsed thee times with 0.1 M PBS for 10 minutes, incubated for 2 hours at room temperature in blocking solution (containing 10% normal horse serum (NHS) and 0.5% triton diluted in 0.1 M PBS), rinsed briefly in 0.1 M PBS and incubated overnight with a primary antibody for vesicular glutamate transporter type 2 (VGluT2) (anti-guinea pig, Millipore Bioscience; diluted 1:2000 in 0.1 M PBS containing 1% NHS and 0.5% Triton-X). Slices were then rinsed four times in 0.1 M PBS for 10 minutes before being incubated for at least 2 hours with secondary antibody anti-Guinea Pig Cy5 (diluted 1:200 in in 0.1 M PBS containing 1% NHS and 0.5% Triton-X). Sections were rinsed four times in 0.1 M PBS for 10 minutes, mounted with Vectashield mounting medium and imaged using a confocal microscope (Leica LS8; 63x objective). Fast Blue labeled cells with overlapping Venus-labeled + VGluT2 +ve axons (with 1 μm) were manually counted.

#### Motor thalamic activation

For optogenetic activation of MTh_DN/IPN_ neurons or their axon terminals in CFA, 250 nl of AAV1-CAG-ChR2-Venus (2.3x10^12^ GC/ml, Addgene 20071; control virus: AAV2-CAG-mCherry (5.2x10^11^ GC/ml)) was injected into contralateral MTh_DN/IPN_ (AP: −1.1, ML: 1.0, DV: −3.4 mm). For direct MTh_DN/IPN_ stimulation, an optic fiber (200 μm diameter, 0.39 NA; Thorlabs) was implanted ∼300 μm dorsal to the viral injection site (sealed with RelyX Unicem2 Automix cement, 3M) and trains of pulsed 473 nm light (5-8 mW, 16.6 Hz pulse frequency, 33.3% duty cycle) were delivered using a solid-state laser (DPSS, Civillaser, China) and shutter (LS3S2T1, Uniblitz) controlled by an Arduino control system, coupled to the implanted optic fiber by means of an optic patch cable (Thorlabs, FT200UMT). For direct simulation of MTh_DN/IPN_ axon terminals, a tapered optic fiber (Optogenix, Italy) was implanted to a depth of 1 mm at the center of CFA (AP: 0.6, ML: 1.6, DV: −1.0 mm) and 12 mW, 473 nm light was delivered as above. Prior to optogenetic stimulation experiments, mice were trained to expert level performance and habituated to light emanating from an uncoupled optic patch cable and the sound of shutter activation. During habituation and experimental sessions, mice were exposed to 3 different trial types: (1) cue and shutter; (2) laser and shutter; and (3) shutter only. Trials were presented with the following pattern: 1, 1, 3, 1, 1, 2,… repeating for 30 minutes. MTh_DN/IPN_ axon terminal stimulation in CFA was performed on the following day, while whole-cell patch-clamp recordings from CFA were performed in combination with direct MTh_DN/IPN_ stimulation in a separate cohort of mice. To investigate the effects of behavioral context, mice which had previously undergone MTh_DN/IPN_ stimulation were head restrained above a 3D printed baseplate (Wanhao i3 Duplicator) without support/movable levers or reward spout (ABC), or within the same lever pressing apparatus with the reward and moveable levers replaced by a 3D printed platform (ABC2), and habituated for 2 sessions, interleaved with normal training to ensure that the cued motor behavior was not extinguished. To compare effects of MTh_DN/IPN_ stimulation in the learned and altered behavioral contexts, mice first underwent a 15-minute optogenetic stimulation protocol in the learned context, before being exposed to an identical 15-minute optogenetic stimulation protocol in one of the two altered behavioral contexts (ABC or ABC2). To investigate the effects of MTh_DN/IPN_ stimulation during freely moving behavior, mice were placed in an open field arena (dimensions 30 × 20 cm) with a camera phone (Samsung Galaxy S5) recording the full arena from beneath. A patch cable was coupled to the MTh_DN/IPN_ optic fiber, and mice underwent a 15-minute optogenetic stimulation protocol. Peri-trial movements were classified from the videos as push-like (a single movement of the left forepaw in a defined forward direction), discrete (a single movement of the left forepaw in any other direction), locomotion (> 2 consecutive steps/strides made by the left forelimb) and grooming, by two researchers and cross validated.

For histological confirmation of the injection site and optic fiber placement, mice were transcardially perfused, decapitated and the whole head (including headplate and optic fiber) was post-fixed in 4% PFA for 2 days to improve preservation of the optic fiber tract. Coronal sections (60 μm) of CFA and MTh_DN/IPN_ were cut with a vibratome, mounted with Vectashield, and imaged using a slidescanner (Axioscan, Zeiss). The center of the optic fiber (COF) was defined as the most ventral extent of the optic fiber tract across all slices from each brain as measured from the pial surface. Where tracts of equal depth were present, the coronal section containing the largest diameter tract tip was identified as the COF. The expression of ChR2-Venus in MTh_DN/IPN_ was coarsely defined by first referencing three coronal slices (120 μm spacing) centered on the COF to the Paxinos and Franklin Mouse Brain Atlas before manually evaluating the proportion of each of the principle motor thalamic nuclei (AM, anteromedial; VL, ventrolateral; VPM, ventral posteromedial nucleus; VPL, ventral posterolateral; VM, ventromedial) containing fluorescence, and categorizing three levels based on expression covering 0%–5%, 5%–50% and 50%–100% of each nucleus. Data were not included from mice in which the COF was misaligned to virus expression. To investigate whether photostimulation of MTh_DN/IPN_ evokes tongue movements, a ROI was drawn in front of the mouth and tongue movements were detected using a motion index threshold (see below).

The proportion of full and partial push-like movements in cue- and laser- trials were calculated by correcting for the behavioral “error” rate, i.e., subtracting the proportion of pushes observed in shutter only trials (trial type 3) to obtain a lower bound. ΔV_m_ trajectories for both cue-evoked and photoactivation-evoked movement trials were calculated as described previously, and trial-by-trial ΔV_m_ changes were based on comparing the 200ms pre-cue or pre-photoactivation epoch with the 200 ms peri-movement epoch within each trial.

#### Cerebellar-motor thalamic pathway tracing and activation

For tracing and activation of the dentate/interpositus-motor thalamus pathway, AAV1-CAG-ChR2-Venus (2.3x10^12^ GC/ml, Addgene 20071) was injected unilaterally into ipsilateral dentate (AP: −6.0, ML: −2.25, DV: −2.6 & −2.2 mm) and interpositus (AP: −6.0, ML: −1.75, DV: −2.4 mm) cerebellar nuclei, with 75 nl injected at each depth within each nucleus. For activation of dentate/interpositus axons in motor thalamus, an optic fiber (200 μm diameter, 0.39 NA; Thorlabs) was implanted into contralateral MTh_DN/IPN_ (AP: −1.1, ML: 1.0, DV: −3.2 mm) and trains of pulsed 473 nm light (8 mW, 16.6 Hz pulse frequency, 33.3% duty cycle) were delivered as previously described. Mice were trained to expert level performance, habituated to light emanating from an uncoupled optic fiber and the sound of shutter activation and exposed to the same alternating trial structure as for MTh_DN/IPN_ activation experiments described previously. For histological confirmation of the injection site, mice were transcardially perfused and coronal sections (60 μm) of MTh_DN/IPN_ and DN/IPN were cut with a vibratome, mounted, and imaged using a slidescanner (Axioscan, Zeiss). Optic fiber location within MTh_DN/IPN_ was ascertained as described previously. The expression of ChR2-Venus in DN/IPN was coarsely defined by first referencing the three coronal slices centered on the DN and IPN to the Paxinos and Franklin Mouse Brain Atlas before manually evaluating the proportion of each of the cerebellar (DN, dentate; IPN, interpositus; FN, fastigial) and vestibular nuclei containing fluorescence, and categorizing three levels based on expression covering 0%–5%, 5%–50% and 50%–100% of each nucleus. Data were not included from mice that had insufficient ChR2-Venus expression in DN and IPN, or in which the COF was not aligned to MTh_DN/IPN_.

To map DN/IPN projections to CFA-projecting neurons in MTh_DN/IPN_, some mice underwent surgery to perform an additional craniotomy above contralateral CFA (AP: 0.6, ML: 1.6 mm), where Fast Blue retrograde tracer (Polysciences; 0.2% Fast Blue in 1 M PB with 0.2% DMSO) was injected at four points equidistant from the center of the CFA craniotomy, with 100 nl injected at two depths, −800 μm and −400 μm below the pial surface. After recovery, mice were returned to the home cage for a further 5 days, before being transcardially perfused. Coronal sections (60 μm) of CFA, MTh_DN/IPN_ and DN/IPN were cut with a vibratome, mounted using Vectashield, and imaged using a confocal microscope (Leica SP8; 20x objective). Raw data images of coronal sections of the motor thalamus were manually referenced to the Paxinos and Franklin Mouse Brain Atlas, aligned and cropped to the same exact motor thalamic subregion. These cropped images were combined into a stack using Fiji and an average intensity projection of each channel (Venus & Fast Blue) was calculated. The resultant average image for each channel were considered as a matrix of gray-scale pixel values in MATLAB, and to calculate a matrix of proportional overlap of the two channels, the two matrices were square-rooted and then multiplied together. A 2-D Gaussian smoothing kernel with SD = 5 pixels was then used to smooth the resultant image which was then remapped with the Jet colormap. For the density plots of individual channels, the average projection matrices were similarly smoothed and remapped.

To assess the density of CFA-projecting neurons in ventrolateral motor thalamus, 200 nl of CTB-Alexa647 (ThermoFisher) was injected into contralateral CFA (AP: 0.6, ML: 1.6, DV: −0.7 mm). After recovery, mice were returned to the home cage for ∼7 days before being perfused. Coronal sections (100 μm) of MTh_DN/IPN_ were collected, counterstained with NeuroTrace Nissl blue (ThermoFisher), mounted using Vectashield mounting medium and imaged with a confocal microscope (Leica LSM800). Cells were counted in a representative 300 × 300 μm region and counts were independently verified.

To quantify vestibular nuclei projections to motor thalamus, we performed a craniotomy above contralateral MTh_DN/IPN_ (AP: −1.1, ML: 1.0 mm) and injected 100 nl of Fast Blue at a depth of 3.4 mm below the pial surface. After recovery, mice were returned to the home cage for 5 days, before being perfused, and brains processed and imaged as described above.

#### Monosynaptic retrograde rabies tracing

For monosynaptic retrograde rabies tracing, conditional expression of TVA receptor was achieved by injecting 60 nl of AAV2/1-CAG-FLEX-mTagBFP2-2A-TVA (9.0x10^12^ GC/ml) into contralateral M1_FL_ (AP: 0.6, ML: 1.6, DV: −0.7 mm) of three Rbp4-Cre mice. Pseudotyped SADΔG-mCherry(EnvA) rabies virus (produced as previously described (Wickersham et al., 2007, 2010) was injected into CFA three weeks after the initial injections. Mice were perfused seven days post-rabies virus injection. Coronal sections (60 μm) were cut, mounted and imaged using a Nanozoomer Slide Scanner (Hamamatsu, 20x objective). Raw data images were manually referenced to the Paxinos and Franklin Mouse Brain Atlas and the distribution of fluorescence was manually outlined and independently verified.

#### Extracellular recording and spike sorting

To compare neural activity during cue-evoked and photoactivated movements, extracellular unit recordings in CFA were performed using acutely implanted silicone probes (Neuropixels Phase 3B probes, IMEC). Data were acquired from the 384 channels closest to the probe tip. Data were acquired with SpikeGLX software at 30 KHz with an amplifier gain of 500 for each channel and high-pass filtered with a cutoff frequency of 300 Hz. Spike sorting was performed using Kilosort3 to automatically cluster units from raw data (Pachitariu et al., 2016). The resulting spike clusters were manually curated using Phy (https://github.com/cortex-lab/phy), and any unit with sufficient refractory period violations, inconsistent waveform amplitude across the duration of the recording, or clipped amplitude distribution was excluded from analyses. Probe location was confirmed via DiI (Thermofisher) reconstruction of the recording track, and units from 500-1200 μm below the pial surface were included in our analyses. To detect changes in activity, firing rates were calculated by convolving spike times with a 200 ms Gaussian kernel and mean changes in firing rate were calculated by subtracting the firing rate during a baseline period (200 ms period before cue or laser presentation) from a response period (−100 to +100 ms peri-movement onset). Significant changes were identified by comparing bootstrapped 95% confidence intervals of these mean changes in firing rates to 0. Firing rate trajectories are presented as spike times convolved with a 50 ms Gaussian kernel. Spike time rasters are presented showing a random sample of cue trials matching the number of photostimulation trials. Spike widths were calculated as the duration from trough to peak of the spike waveform.

#### Quantifying muscimol diffusion

To measure muscimol diffusion, a small volume of muscimol-BODIPY TMR-X Conjugate (ThermoFisher Scientific; dissolved in 0.1 PBS w/1% dimethyl sulfoxide) was injected into MTh_DN/IPN_ (200 nl of 1 mM), CFA (100 nl of 2 mM at −700 μm and −400 μm below the pial surface at 5 sites centered on CFA) or DN/IPN (100 nl and 50 nl of 1mM, respectively). To mark the center of injection, pipettes were backfilled with a small volume (∼20 nl) of green (505 nm) retrobeads (Lumafluor Inc.) prior to filling with muscimol-BODIPY. Following injection, animals were transcardially perfused and brains snap-frozen on dry ice within 10 minutes of completion of muscimol injection. Brains were stored on dry ice, coronal sections (60 μm) collected with a cryostat (Leica) at −20°C and imaged with a light microscope (Leica DMR, 5x objective). We assumed maximum fluorescence ≈maximum injected concentration and that grayscale pixel intensity was proportional to muscimol-BODIPY concentration. Therefore, pixel values were thresholded at the equivalent pixel value of an EC_20_ concentration of muscmiol and fluorescence outlines were drawn to generate a ‘spread profile’. Green retrobeads were used to mark the center of each injection, and images were aligned to the injection center of gravity. From the aligned profiles, a modal spread profile (i.e., pixels with positive grayscale values across all mice) was generated and aligned to the Paxinos and Franklin Mouse Brain Atlas.

To assess the functional time course of muscimol inactivation, a silicone probe was vertically implanted into CFA (AP: 0.6, ML: 1.2-1.6, DV: −2.0 mm) of naive head restrained mice at rest. 15 minutes of baseline activity was recorded, after which 200 nl of 1 mM muscimol (containg 1% w/v red retrobeads) was injected at a point 500-700 μm horizontal from the shank of the probe (AP: 0.6, ML: 1.8-2.4, DV: −1.0 mm). The recording was continued for a further 30 minutes before perfusing and collecting tissue as described previously. Probe location was confirmed by DiI labeling, neural activity was spike sorted and analyzed as previously described, individual units were localized and the change in spike rate over time was correlated with the distance of each individual unit from the center of muscimol injection.

#### Forelimb kinematic tracking

Behavior was recorded using a high-speed camera (Pharmacological experiments: Genie HM640, Dalsa; optogenetic experiments: Mako U U-029, Allied Vision) and acquired with Streampix 7 (Norpix), synced using a TTL output from the DigiData 1440 DAC interface. Forepaw and wrist positions during pharmacological inactivation experiments were calculated by tracking forepaw markers using a custom written tracking script in Blender (2.79b, Blender Foundation). Directional tracking of forelimb movement in the learned/altered behavioral contexts was performed using Deep Lab Cut, a markerless video tracking toolbox (Mathis et al., 2018). Initial paw vector trajectories were plotted for the 50 ms post movement onset epoch in the learned behavioral context (LBC), and for the altered behavioral contexts (ABC & ABC2) we plotted trajectories in the epoch 50 ms after the LBC median reaction time. Push-like movements were defined as trials with an initial paw trajectory vector between 170° and 210°, and manually verified. To measure gross forelimb movement, we defined a region-of-interest (ROI) covering the contralateral (left) forelimb and calculated a motion index (MI) for each successive frame f as $MI_{f}=\sum_{i=1}^{N}{\left(c_{f+1,i}-c_{f,i}\right)}^{2}$, where $c_{f,i}$ is the grayscale level of the pixel of the ROI, pixels per ROI (Schiemann et al., 2015). Movement trials were defined by calculating the MI > θ within 500 ms of cue/photostimulation onset, with the threshold θ defined as three standard deviations above the mean MI for detecting forelimb movements, and 10 standard deviations above the mean MI for detecting licking.

### Quantification and statistical analysis

Data analysis was performed using custom-written scripts in MATLAB or Python3 and code will be made available on request. All statistical details of experiments can be found in the figure legends, including description of the specific test used and sample sizes. Data are reported as mean ± 95% bootstrapped confidence interval, 10,000 bootstrap samples, unless otherwise indicated. Where multiple measurements were made from a single animal, suitable weights (proportional to the contribution from each animal) were used to evaluate summary population statistics and to obtain unbiased bootstrap samples. Statistical comparisons using the significance tests stated in the main text were made in MATLAB, and statistical significance was considered when p < 0.05 unless otherwise stated. Data were tested for normality with the Shapiro–Wilk test, and parametric/non-parametric tests were used as appropriate and as detailed in the text. Data inclusion/exclusion criteria have been listed throughout the method details section where relevant.

## Supplemental information

Document S1. Figures S1–S6 and Table S1

Document S2. Article plus supplemental information
