# Supplementary material for: A cerebellar-thalamocortical pathway drives behavioral context-dependent movement initiation
Source: Neuron. 2021 Jul 21;109(14):2326–2338.e8. doi: 10.1016/j.neuron.2021.05.016 (PMC8315304; doi:10.1016/j.neuron.2021.05.016)

Neuron, Volume *109*

Supplemental information

A cerebellar-thalamocortical pathway drives behavioral context-dependent movement initiation

Joshua Dacre, Matt Colligan, Thomas Clarke, Julian J. Ammer, Julia Schiemann, Victor Chamosa-Pino, Federico Claudi, J. Alex Harston, Constantinos Eleftheriou, Janelle M.P. Pakan, Cheng-Chiu Huang, Adam W. Hantman, Nathalie L. Rochefort, and Ian Duguid

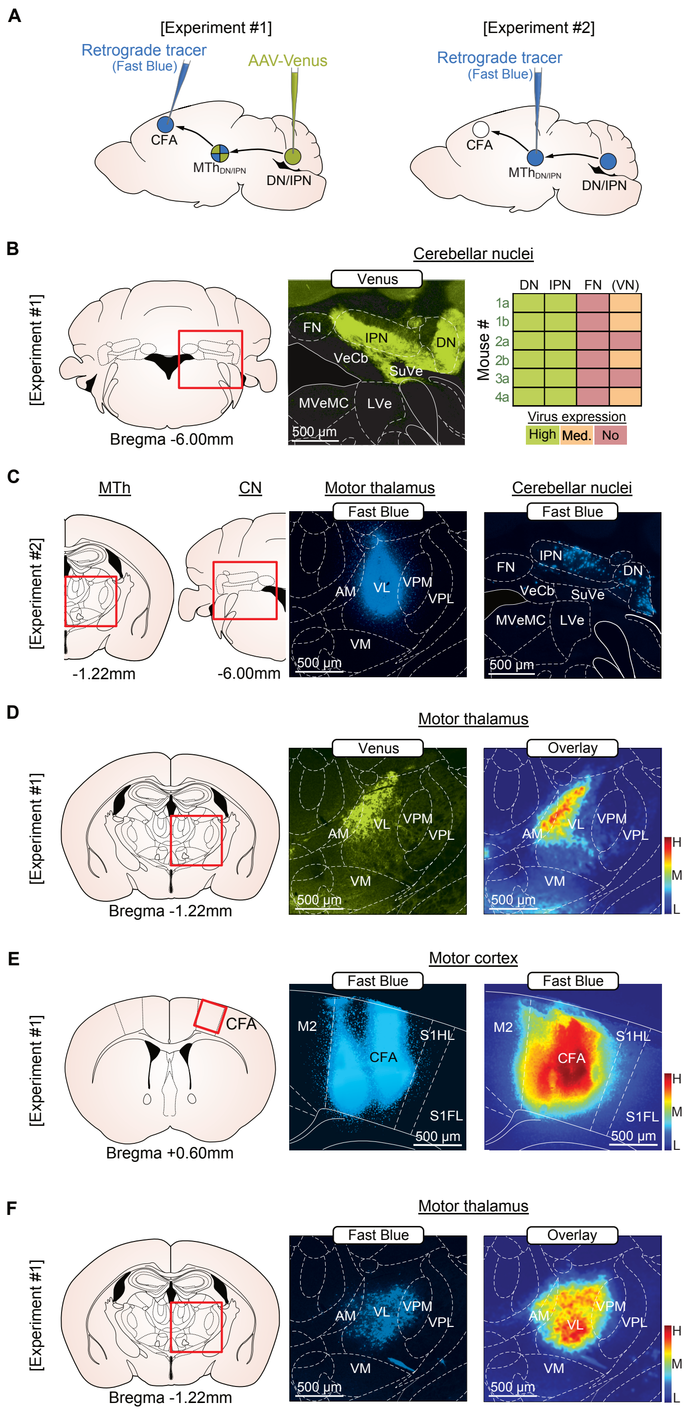

Figure S1. Mapping the DN/IPN thalamocortical pathway, related to Figure 1.
(A) *Experiment #1*, retrograde tracing of CFA-projecting neurons (Fast Blue) and anterograde tracing of DN/IPN axons (AAV-Venus) in motor thalamus. *Experiment #2*, retrograde tracing of VAL-projecting neurons in cerebellar nuclei (Fast Blue). (B) *Left & middle*, Virus labelling of cerebellar nuclei. *Right*, quantification of Venus expression in cerebellar and vestibular nuclei (red/no = <5 %, orange/medium = 5-50 % & green/high = 50-100 % expression within each nuclei) (bilateral injection, n = 6 slices from N = 4 mice). DN, dentate nucleus; IPN, interpositus nucleus; FN, fastigial nucleus, VN, vestibular nuclei including: VeCb, vestibulocerebellar nuclei; SuVe, superior vestibular nucleus; MVeMC, medial vestibular nucleus magnocellar part; LVe, lateral vestibular nucleus. (C) *Left & middle*, Fast Blue injection site in motor thalamus centered on VL. *Right*, retrograde labelling of cerebellar and vestibular nuclei neurons. AM, anteromedial; VL, ventrolateral; VPM, ventral posteromedial; VPL, ventral posterolateral; VM, ventromedial thalamic nuclei. (D) *Left & Middle*, Anterograde labelling of DN/IPN axons in motor thalamus. *Right*, average density of DN/IPN axons across motor thalamic nuclei (N = 6 slices from 4 mice). Scale bar, H - high, M - medium, L - low-level expression. (E) *Left & centre*, Fast Blue injection site in CFA. Right, average density of Fast Blue in CFA (N = 6 slices from 4 mice). Scale bar, H - high, M - medium, L - low-level fluorescence. (F) *Left & Middle*, Retrograde labelling of CFA-projecting thalamic neurons. *Right*, average density of CFA-projecting neurons across thalamic nuclei (N = 6 slices from 4 mice). Scale bar, H - high, M - medium, L - low-level expression.

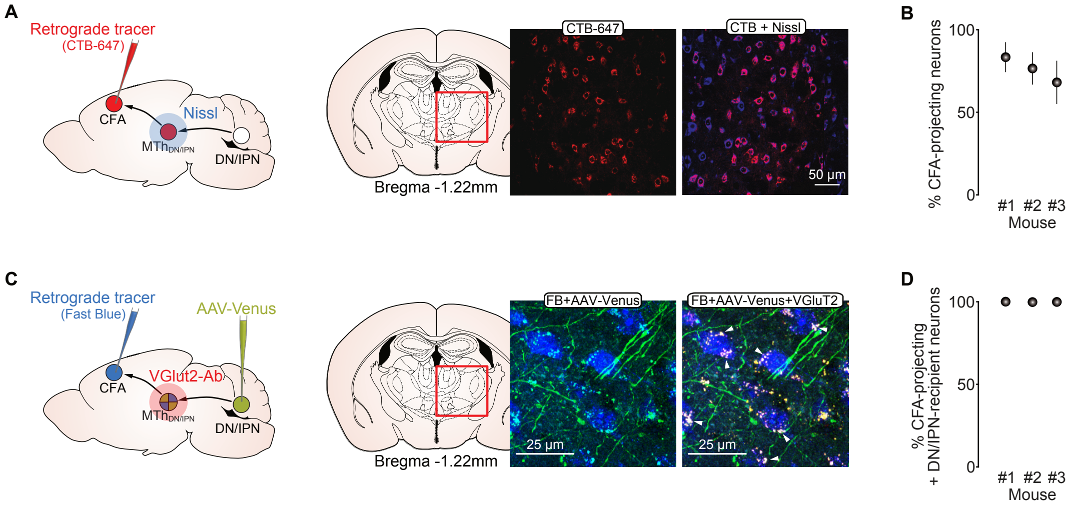

Figure S2. Quantification of CFA-projecting & DN/IPN-recipient neurons in ventral motor thalamus, related to Figure 1.
(A) *Left*, Nissl stain and retrograde labelling of CFA-projecting neurons in ventrolateral thalamus (CTB-647). *Right*, retrograde labelling of CFA-projecting neurons in ventrolateral thalamus (CTB-647) with all neurons labelled with Nissl. (B) Proportion of CFA-projecting motor thalamic neurons in VL thalamus (N = 3 mice, 4-6 slices per mouse, mean ± bootstrapped 95% CI). Filled circles represent population means ± 95% CI. (C) *Left & right,* retrograde tracing of CFA-projecting neurons (Fast Blue) and anterograde tracing of DN/IPN axon terminals (AAV-Venus + VGluT2) in ventrolateral motor thalamus. (D) Proportion of CFA-projecting motor thalamic neurons in VL thalamus that receive glutamatergic synaptic input from dentate/interpositus nuclei (N = 3 mice, 2-4 slices per mouse, mean ± bootstrapped 95% CI). Filled circles represent population means ± 95% CI.

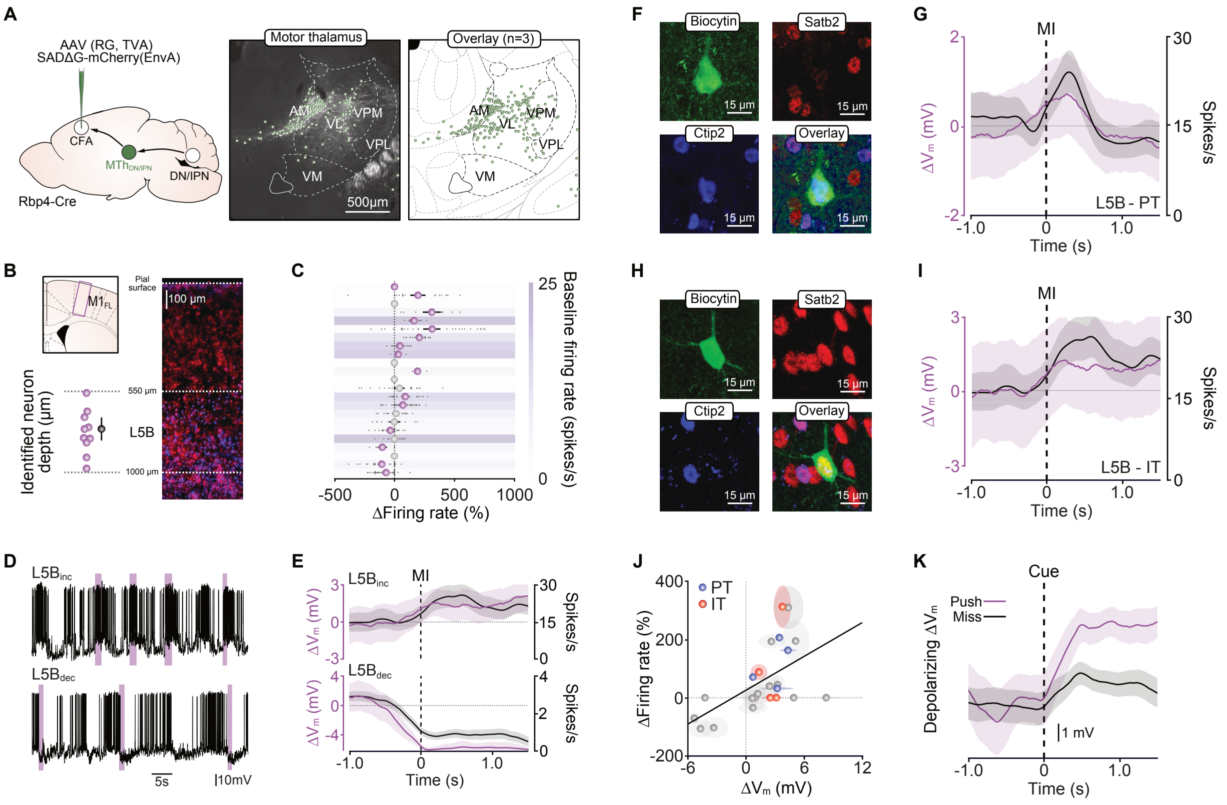

Figure S3. Membrane potential dynamics and projection class identity of CFA L5B neurons, related to Figure 3.
(A) *Left*, Monosynaptic rabies tracing strategy: injection of AAV2/1-CAG-FLEX-mTagBFP2-2A-TVA & SADΔG-mCherry(EnvA) into caudal forelimb motor area (CFA) of an Rbp4-Cre mouse. *Centre & Right*, CFA-projecting neurons in ventral motor thalamus. AM, anteromedial; VL, ventrolateral; VPM, ventral posteromedial; VPL, ventral posterolateral; VM, ventromedial thalamic nuclei. (B) *Top left*: Schematic coronal brain slice showing location of CFA. Purple rectangle depicts the expanded view shown on the right. *Right:* Distribution of PT-type (blue, Ctip2 staining) and IT-type (red, Satb2 staining) projection neurons in layer 5B of CFA. *Bottom left:* depth of recovered layer 5B neurons as measured perpendicularly from the pial surface (n = 11/23 neurons identified, black symbol represents mean ± 95% CI). (C) Average firing rate change ± 95% CI as a function of baseline firing rate. Gray dots represent individual trials, purple symbols represent significant changes in firing rate, gray symbols represent non-significant changes, defined by comparing 95% bootstrapped confidence intervals. (D) Voltage traces from a (*top*) depolarizing and (*bottom*) hyperpolarizing layer 5B neuron across multiple trials. Filled purple bars depict push trials. (E) Average subthreshold ΔV_m (purple) and firing rate (FR, black) trajectories for the layer 5B neurons shown in *(B)* aligned to movement initiation (MI). Thick lines represent the mean ± 95% CI. (F) Biocytin staining (green) and post-hoc immunohistochemical staining for Satb2 (red) and Ctip2 (blue) confirmed the PT-type projection class identity of an individually recorded layer 5B pyramidal neuron. (G) Mean subthreshold V_m and firing rate of the layer 5B PT-type projection neuron depicted in *(F)*. (H) Biocytin staining (green) and post-hoc immunohistochemical staining for Satb2 (red) and Ctip2 (blue) confirmed the IT-type projection class identity of an individually recorded L5B pyramidal neuron. (I) Mean subthreshold V_m and firing rate of the L5B IT-type projection neuron depicted in *(H)*. (J) Correlation between movement-related subthreshold ΔV_m and firing rate changes. Blue/red symbols represent means ± 95% CI from individual PT-/IT-type neurons respectively, black line is a linear fit to the data. Gray symbols represent means ± 95% CI from neurons where projection class identity was not determined (N = 23 neurons from 23 mice). (K) Example cue aligned push-trial mean ΔVm (purple) vs miss-trial mean ΔVm (black) trajectories from a representative layer 5B neuron in CFA.

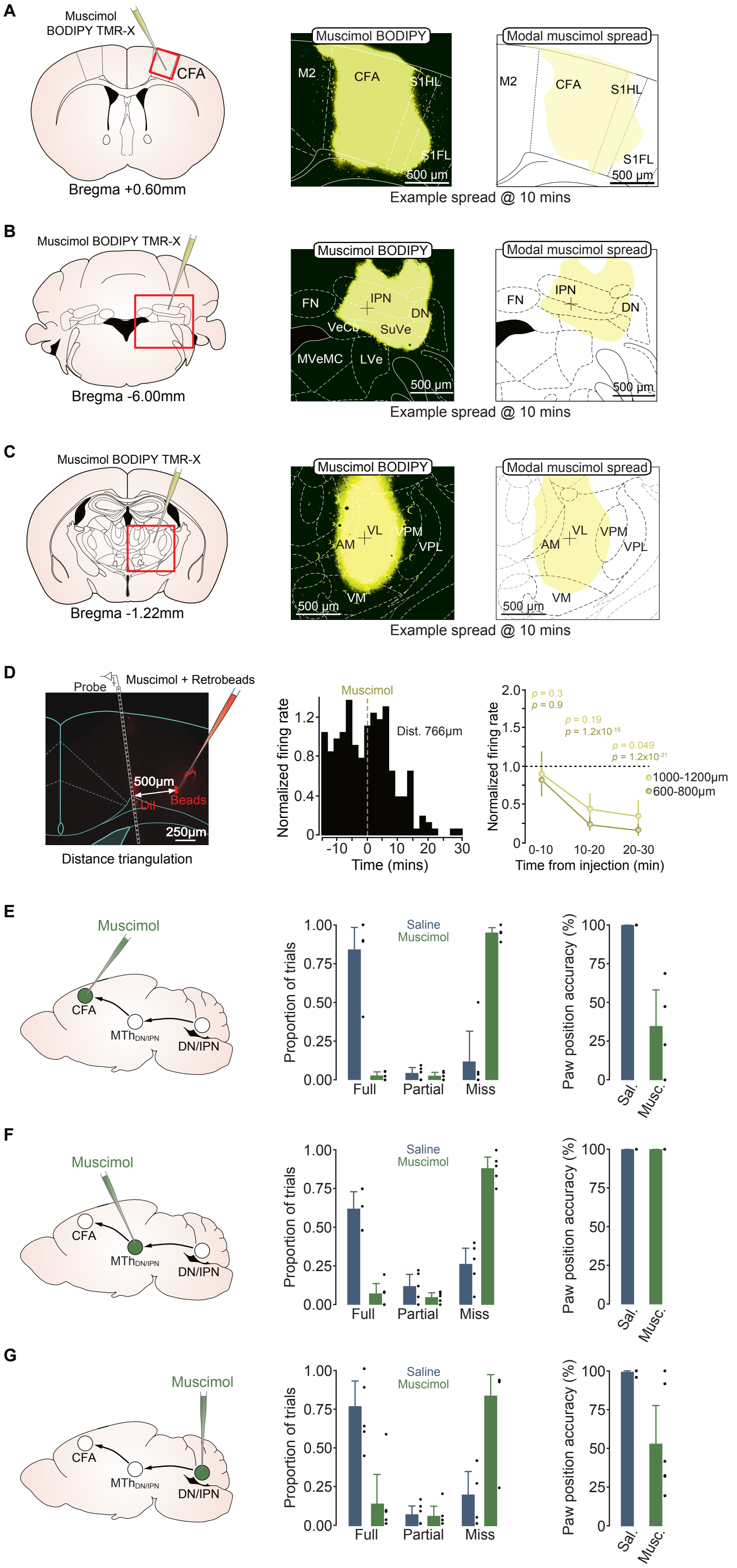

Figure S4. Diffusional spread and behavioral effects of muscimol in CFA, cerebellar nuclei and ventral thalamus, related to Figure 4.
(A) *Left*, injection of muscimol BODIPY TMR-X into CFA. *Middle*, example image of fluorescent muscimol spread in CFA at 10 mins post injection. *Right*, Modal spread of fluorescent muscimol in CFA (i.e., area in which fluorescence is present across all mice) (N = 3 mice). M2, secondary motor cortex; CFA, caudal forelimb area; S1HL, primary hindlimb somatosensory cortex. (B) *Left*, injection of muscimol BODIPY TMR-X targeted to dentate and interpositus cerebellar nuclei. *Middle*, example image of fluorescent muscimol spread across cerebellar nuclei at 10 mins post injection. *Right*, Example of diffusional spread outline used to calculate the modal spread shown in in Figure 4B. DN, dentate nucleus; IPN, interpositus nucleus; FN, fastigial nucleus, VN, vestibular nuclei including: VeCb, vestibulocerebellar nuclei; SuVe, superior vestibular nucleus; MVeMC, medial vestibular nucleus magnocellar part; LVe, lateral vestibular nucleus. The cross represents the median point of injection located using fluorescent retrobeads (N = 4 mice). (C) *Left*, injection of muscimol BODIPY TMR-X targeted to $MTh_{DN/IPN}$. *Middle*, example image of fluorescent muscimol spread in ventral thalamus at 10 mins post injection. *Right*, Example of diffusional spread outline used to calculate the modal spread shown in in Figure 4C. AM, anteromedial; VL, ventrolateral; VPM, ventral posteromedial; VPL, ventral posterolateral; VM, ventromedial thalamic nuclei. The cross represents the median point of injection located using fluorescent retrobeads (N = 4 mice). (D) *Left*, example silicon probe tract through cortex visualized using DiI and muscimol injection site visualized using red fluorescent retrobeads. The distance between the centre of the bead injection and the electrode position of each recorded unit was used to group neurons (600-800 µm or 1000-1200µm). *Middle*, normalized firing rate as a function of time from muscimol injection in an example CFA neuron. *Right*, normalized suppression of firing rate in CFA neurons as a function of time and distance. Group: 600-800 µm, n = 43 neurons; 1000-1200 µm, n = 25 neurons, one-way t-test for significant differences from baseline at each time point. (E) *Left*, Muscimol injection into CFA. *Middle*, Proportion of full / partial lever pushes and miss trials 10 minutes after saline (blue) or muscimol (green) injection into CFA. Black dots represent data from individual mice, bars represent population means ± 95% CI. *Right*, Paw position accuracy at the point of cue presentation and 10 mins after saline (Sal., blue) or muscimol (Musc., green) injection into CFA (N = 5 and 5 mice). (F) Same as *(E)* but with injections targeted to $MTh_{DN/IPN}$. (N = 5 and 5). (G) Same as *(E)* but with injections targeted to DN/IPN (N = 5 and 5 mice).

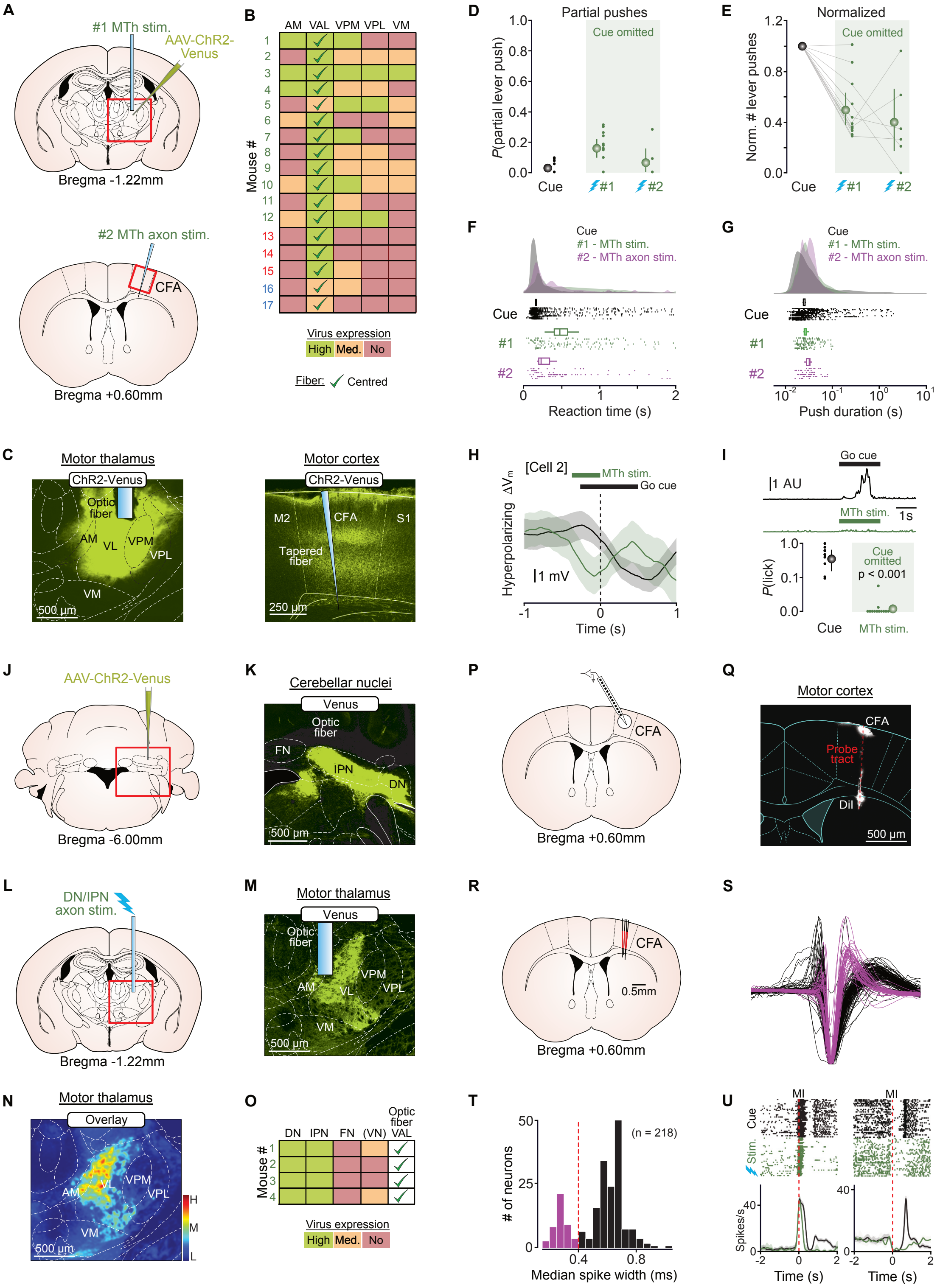

Figure S5. Photoactivation of the cerebello thalamocortical pathway, related to Figure 5.
(A) Injection of AAV-Venus-ChR2 targeted to $MTh_{DN/IPN}$ with optic fiber chronically implanted directly above thalamus (*top, #1*) or a tapered optic fiber acutely implanted into CFA (*bottom, #2*). (B) Quantification of viral expression in ventral motor thalamus (red/no = <5 %, orange/medium = 5-50 % & green/high = 50-100 % expression within each nuclei, green ticks represent correct fiber placement above ventral anterolateral thalamus). Data from mice 1-12 are included in Figure 5B, mice 13-15 displayed no behavioral effects upon photoactivation, mice 16-17 were transduced with AAV-mCherry as controls. (C) Expression of ChR2-Venus and fiber placement in (*left*) ventrolateral thalamus and (*right*) CFA. AM, anteromedial; VL, ventrolateral; VPM, ventral posteromedial; VPL, ventral posterolateral; VM, ventromedial thalamic nuclei. M2, secondary motor cortex; CFA caudal forelimb area; S1, primary sensory cortex. (D) Probability of partial lever push movements evoked by an auditory go cue (black) or photoactivation of $MTh_{DN/IPN}$ (#1) or $MTh_{DN/IPN}$ axons in CFA (#2) in the absence of a go cue (green). Colored dots represent data from individual mice, colored circles represent mean ± 95% CI. For Cue, #1 and #2, N = 12, 12 and 6 mice, respectively. (E) Normalized number of lever pushes evoked by an auditory go cue (black) or photoactivation of $MTh_{DN/IPN}$ (#1) or $MTh_{DN/IPN}$ axons in CFA (#2) in the absence of a go cue (green). Colored dots represent data from individual mice, colored circles represent mean ± 95% CI. For Cue, #1 and #2, N = 12, 12 and 6 mice, respectively. (F-G) Raincloud plots showing the distributions of *(F)* reaction times and *(G)* push durations of cue-evoked (black) and photoactivation (#1 & #2) push trials. Box-and-whisker plots represent bootstrapped estimates of median statistics. (H) Example hyperpolarizing subthreshold V_m change ± 95% CI in a layer 5B projection neuron in response to the cue (black) or photoactivation of $MTh_{DN/IPN}$ (green) in the absence of a cue. Dashed line indicates movement initiation. (I) *Top traces*, average across-trial motion index from an ROI covering the tongue (i.e. licking). Black, cue-evoked licking; green, $MTh_{DN/IPN}$ photoactivation-evoked licking. *Bottom*, probability of licking evoked by an auditory go cue (black) or photoactivation of $MTh_{DN/IPN}$ in the absence of a go cue (green). Colored dots represent data from individual mice, colored circles represent mean ± 95% CI (N = 12 mice). (J) Injection of AAV-Venus-ChR2 targeted to DN/IPN. (K) Expression of ChR2-Venus in DN/IPN. FN, fastigial nucleus; IPN, interpositus nucleus ; DN, dentate nucleus. (L) Photoactivation of DN/IPN axon terminals in $MTh_{DN/IPN}$. (M) Anterograde labelling of DN/IPN axons in motor thalamus and optic fiber placement. (N) Average density of DN/IPN axons across motor thalamic nuclei (N = 4 slices from 4 mice). Scale bar, H - high, M - medium, L - low-level expression. AM, anteromedial; VL, ventrolateral; VPM, ventral posteromedial; VPL, ventral posterolateral; VM, ventromedial thalamic nuclei. (O) Quantification of viral expression (red/no = <5 %, orange/medium = 5-50 % & green/high = 50-100 % expression within each nuclei, green ticks represent correct fiber placement above ventral anterolateral thalamus) (N = 4 mice). FN, fastigial nucleus; IPN, interpositus nucleus ; DN, dentate nucleus. (P) Silicone probe recordings in deep layers of CFA. (Q) Example probe tract in CFA visualized using DiI. (R) Overlay of probe tracts in CFA (N = 4 mice). (S) Overlaid mean spike waveforms for putative interneurons (purple) and pyramidal cells (black). (T) Histogram of spike durations (see methods). (U) Rasters and peri-stimulus time histograms of activity in 2 example deep layer CFA neurons aligned to movement initiation (red dashed line). Black represents cue trials, green represents photoactivation trials.

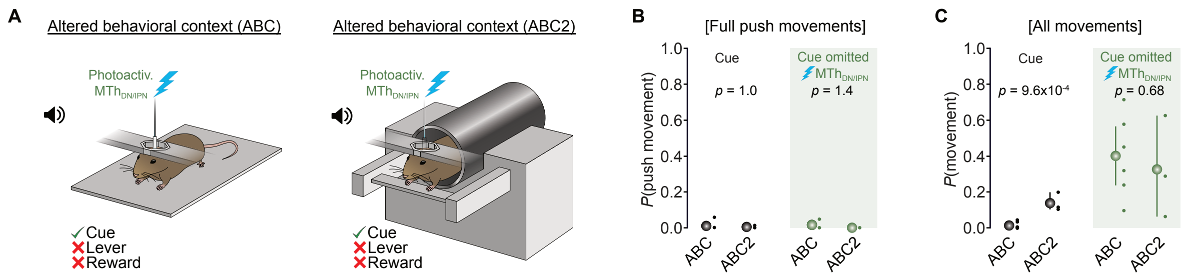

Figure S6. Comparison of photoactivated forelimb movements in two altered behavioral contexts, related to Figure 6.
(A) Photoactivation of $MTh_{DN/IPN}$ in (*left*) an altered behavioral context with flat baseplate (ABC) and (*right*) altered behavioral context that recapitulates LBC mouse posture (ABC2, i.e. horizontal bar positioned at the height of the LBC movable lever - see Figure 6A). (B) Probability of push-like movements evoked by an auditory go cue (black) or photoactivation of $MTh_{DN/IPN}$ in the absence of a go cue (green). Colored dots represent data from individual mice, colored circles represent mean ± 95% CI. ABC, N = 6 mice, ABC2 N = 3 mice. (C) Probability of any forelimb movement evoked by an auditory go cue (black) or photoactivation of $MTh_{DN/IPN}$ in the absence of a go cue (green) in ABC (N = 6 mice) or ABC2 (N = 3 mice).

Table S1. Contributions Matrix

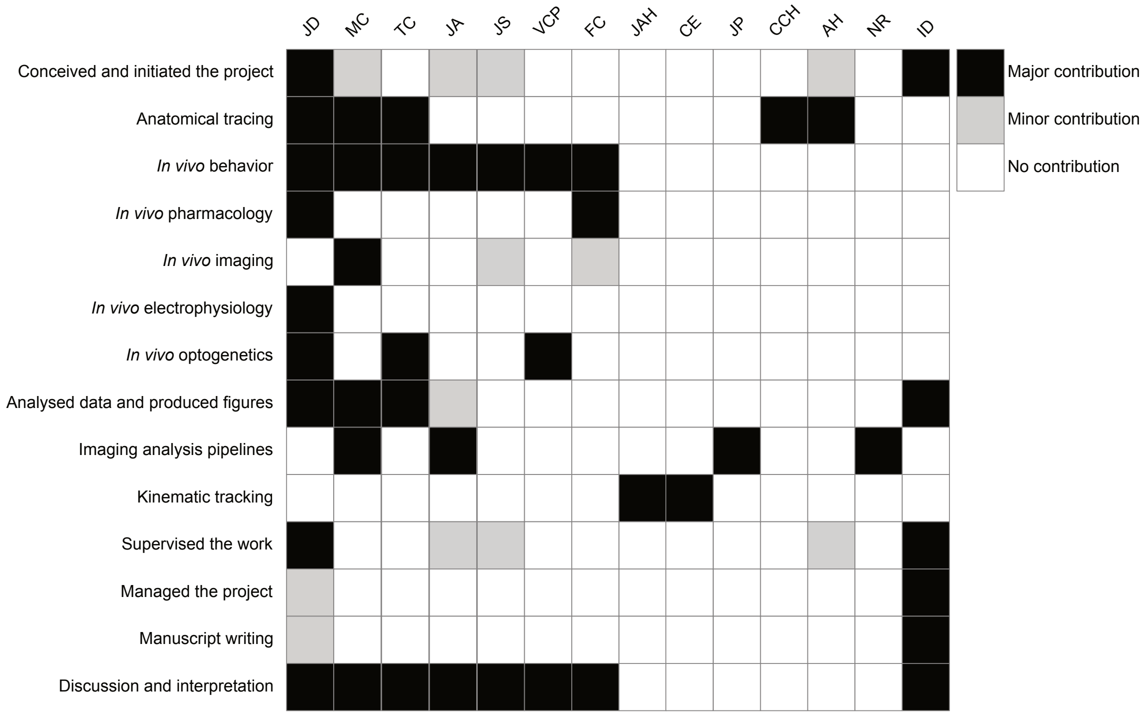

Supplement: Document S1. Figures S1–S6 and Table S1 [file mmc1.pdf]
