# Supplementary material for: A cerebellar-thalamocortical pathway drives behavioral context-dependent movement initiation
Source: Neuron. 2021 Jul 21;109(14):2326–2338.e8. doi: 10.1016/j.neuron.2021.05.016 (PMC8315304; doi:10.1016/j.neuron.2021.05.016)
Supplement: Document S2. Article plus supplemental information [file mmc7.pdf]

Neuron

Article

A cerebellar-thalamocortical pathway drives behavioral context-dependent movement initiation

Graphical abstract

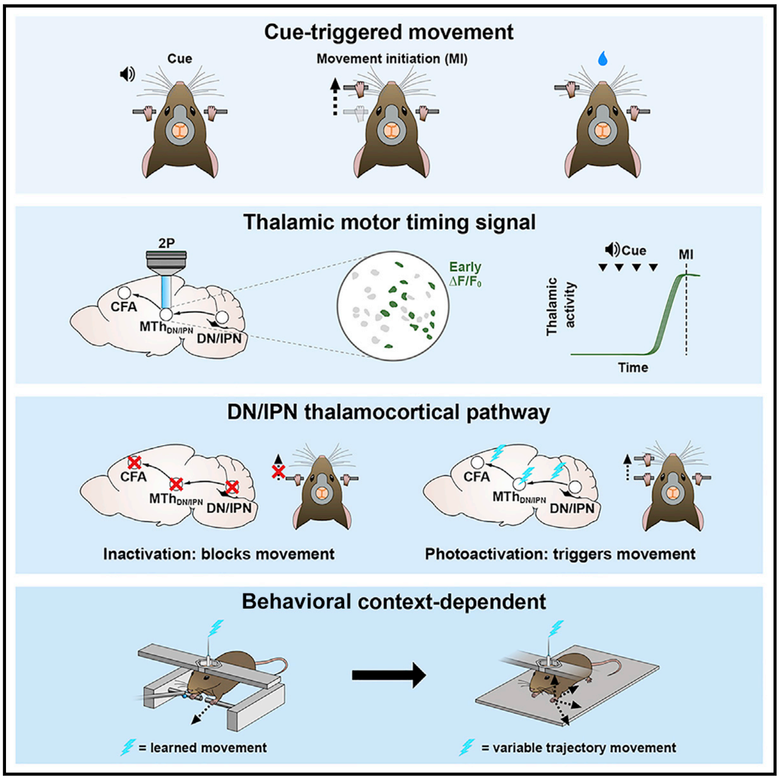

Highlights

- DN/IPN thalamocortical activity conveys a reliable feedforward motor timing signal
- Silencing DN/IPN or recipient regions of thalamus blocks movement initiation
- Photostimulation of the DN/IPN thalamocortical pathway triggers movement
- Thalamocortical activation drives behavioral context-dependent movement initiation

Authors

Joshua Dacre, Matt Colligan, Thomas Clarke, ..., Adam W. Hantman, Nathalie L. Rochefort, Ian Duguid

Correspondence

ian.duguid@ed.ac.uk

In brief

Dacre et al. show the contribution of a cerebellar-thalamocortical pathway to movement initiation. Using gain- and loss-of-function manipulations they demonstrate that output from dentate/interpositus nuclei, via the thalamus, shapes cortical activity dynamics necessary for learned behavioral context-dependent movement initiation.

Dacre et al., 2021, Neuron *109*, 2326–2338
July 21, 2021 © 2021 The Authors. Published by Elsevier Inc.
https://doi.org/10.1016/j.neuron.2021.05.016

CellPress
OPEN ACCESS

Neuron

Article

A cerebellar-thalamocortical pathway drives behavioral context-dependent movement initiation

Joshua Dacre,[1] Matt Colligan,[1,4] Thomas Clarke,[1,4] Julian J. Ammer,[1,4] Julia Schiemann,[1,5] Victor Chamosa-Pino,[1] Federico Claudi,[1,6] J. Alex Harston,[1,7] Constantinos Eleftheriou,[1,2] Janelle M.P. Pakan,[1,8] Cheng-Chiu Huang,[3,9] Adam W. Hantman,[3] Nathalie L. Rochefort,[1,2] and Ian Duguid[1,2,10,*]
[1]Centre for Discovery Brain Sciences, Edinburgh Medical School: Biomedical Sciences, University of Edinburgh, Edinburgh, UK
[2]Simons Initiative for the Developing Brain, Centre for Discovery Brain Sciences, University of Edinburgh, Edinburgh, UK
[3]Janelia Research Campus, HHMI, Ashburn, VA, USA
[4]These authors contributed equally
[5]Present address: Center for Integrative Physiology and Molecular Medicine, Saarland University, Homburg, Germany
[6]Present address: UCL Sainsbury Wellcome Centre for Neural Circuits and Behaviour, London, UK
[7]Present address: Brain and Behaviour Lab, Department of Bioengineering, Royal School of Mines, Imperial College London, UK
[8]Present address: Center for Behavioral Brain Sciences, Institute for Cognitive Neurology and Dementia Research, Otto von Guericke University, Magdeburg, Germany
[9]Present address: Eli Lilly and Company, Lilly Cambridge Innovation Center, Cambridge, MA, USA
[10]Lead contact
*Correspondence: ian.duguid@ed.ac.uk
https://doi.org/10.1016/j.neuron.2021.05.016

SUMMARY

Executing learned motor behaviors often requires the transformation of sensory cues into patterns of motor commands that generate appropriately timed actions. The cerebellum and thalamus are two key areas involved in shaping cortical output and movement, but the contribution of a cerebellar-thalamocortical pathway to voluntary movement initiation remains poorly understood. Here, we investigated how an auditory "go cue" transforms thalamocortical activity patterns and how these changes relate to movement initiation. Population responses in dentate/interpositus-recipient regions of motor thalamus reflect a time-locked increase in activity immediately prior to movement initiation that is temporally uncoupled from the go cue, indicative of a fixed-latency feedforward motor timing signal. Blocking cerebellar or motor thalamic output suppresses movement initiation, while stimulation triggers movements in a behavioral context-dependent manner. Our findings show how cerebellar output, via the thalamus, shapes cortical activity patterns necessary for learned context-dependent movement initiation.

INTRODUCTION

The ability to generate appropriately timed motor actions in response to sensory cues is a hallmark of mammalian motor control. Movement timing is controlled in part by the cerebellum, as dysfunction leads to the execution of poorly timed actions (Bastian and Thach, 1995; Holmes, 1939; Milak et al., 1997; Thach, 1975). However, the pathway and circuit dynamics involved in initiating movements remain unclear. Two distinct pathways could contribute to movement initiation, the cerebellar-rubrospinal tract (Asanuma et al., 1983; Gibson et al., 1985; Teune et al., 2000) or the cerebellar-thalamocortical pathway (Bostan et al., 2013; Gornati et al., 2018; Horne and Butler, 1995; Kuramoto et al., 2009; Nashef et al., 2019). The latter is supported by neuronal activity in dentate/interpositus nuclei (DN/IPN) and recipient motor thalamic regions preceding cortical activity (Nashef et al., 2018; Strick, 1976; Thach, 1975, 2014) and movement initiation (Anderson and Turner, 1991; Butler et al., 1992, 1996; Fortier et al., 1989; Harvey et al., 1979; Horne and Porter, 1980; Kurata, 2005; Macpherson et al., 1980; Mushiake and Strick, 1993; Schmied et al., 1979; Strick, 1976; van Donkelaar et al., 1999), while disrupting activity in either region alters the timing of sensory-triggered actions (Meyer-Lohmann et al., 1977; Nashef et al., 2019; Spidalieri et al., 1983; Thach, 1975; van Donkelaar et al., 2000). Beyond a proposed role in movement initiation, DN/IPN and recipient regions of motor thalamus coordinate the timing and accuracy of ongoing movements given that focal inactivation alters endpoint accuracy (dysmetria/hypermetria), reach path curvature and grasping (Becker and Person, 2019; Bracha et al., 1999; Butler et al., 1992; Cooper et al., 2000; Horne and Butler, 1995; Ishikawa et al., 2014; Martin et al., 2000; Mason et al., 1998; Thanawalla et al., 2020), and loss of anticipatory limb adjustments to unexpected obstacles during complex locomotion (Martin et al., 2000; Milak et al., 1997). In contrast, disrupting output from fastigial nucleus results in deficits in posture, locomotion, and motor planning, with minimal effects on reaching (Li et al., 2015; Martin et al., 2000; Thach and Bastian, 2004). Thus, individual cerebellar nuclei provide

 © 2021 The Authors. Published by Elsevier Inc.
This is an open access article under the CC BY license (http://creativecommons.org/licenses/by/4.0/).

CellPress
OPEN ACCESS

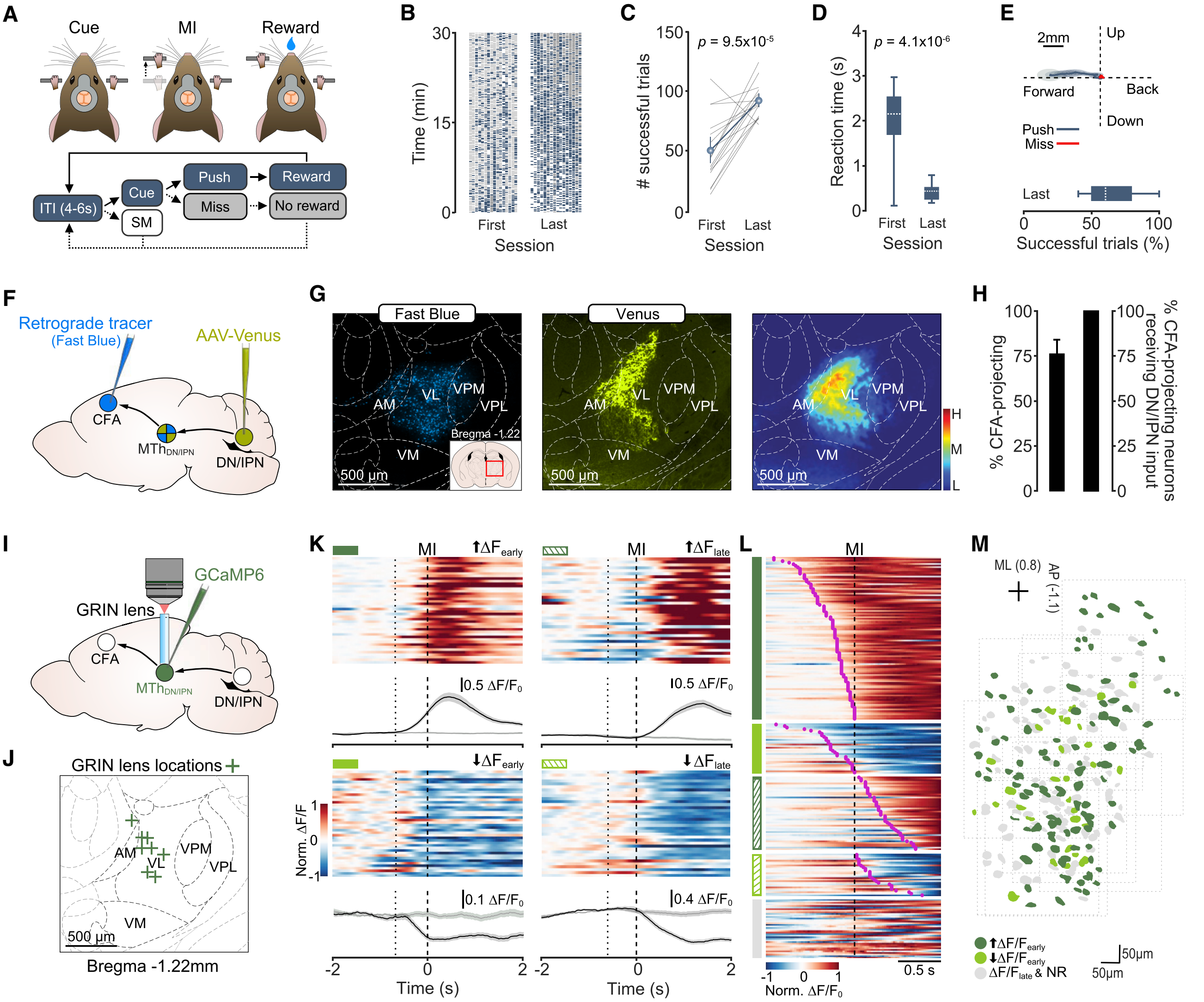

Figure 1. Increased activity dominates trial-to-trial $MTh_{DN/IPN}$ population responses prior to movement initiation

(A) Top: cued forelimb push task. MI, movement initiation. Bottom: behavioral task structure. ITI, inter-trial interval; SM, spontaneous movement.

(B) Trial outcome rasters with each column representing individual mice (N = 16 mice). Blue, push trials; gray, miss trials; white, spontaneous movements.

(C) Number of successful trials per 30-min behavioral session (N = 16 mice, two-sample t test). Mean ± 96% CIs.

(D) Box-and-whisker plots showing mouse reaction times across learning (N = 16 mice, two-sample t test).

(E) Top: forepaw trajectories for push (blue) and miss (red) trials from the mouse in Video S1. Thick line, average paw trajectory overlaid with 95% CI of paw position variance. Bottom: box-and-whisker plot of the percentage of successful trials in “expert” mice.

(F) Mapping the dentate/interpositus thalamocortical pathway. CFA, caudal forelimb area; $MTh_{DN/IPN}$, dentate/interpositus nuclei-recipient region of motor thalamus; DN, dentate nucleus; IPN, interpositus nucleus.

(G) Left: retrograde labeling of CFA-projecting motor thalamic neurons. Middle: anterograde labeling of DN/IPN axons in motor thalamus. Right: density plot of regions of overlap of DN/IPN axons and CFA-projecting neurons across thalamic nuclei (N = 6 hemispheres from four mice). Inset: location of motor thalamic nuclei. AM, anteromedial; VL, ventrolateral; VPM, ventral posteromedial; VPL, ventral posterolateral; VM, ventromedial nuclei.

(H) Left: percentage of CFA-projecting $MTh_{DN/IPN}$ neurons. Right: percentage of CFA-projecting $MTh_{DN/IPN}$ neurons receiving glutamatergic synaptic input from DN/IPN. Mean ± 95% CIs.

(I) Two-photon population calcium imaging in $MTh_{DN/IPN}$.

(J) Locations of GRIN lenses in $MTh_{DN/IPN}$ (N = 8 mice).

(K) Activity of four example $MTh_{DN/IPN}$ neurons. Clockwise from top left: early-onset increase (dark green), late-onset increase (dark green hatching), late-onset decrease (light green hatching), and early-onset decrease (light green). Top: normalized $\Delta F/F_0$ across successive trials. Bottom: $\Delta F/F_0$ mean ± SEM. Black lines, push trials; gray lines, miss trials; dotted lines, median cue onset; dashed lines, movement initiation (MI).

(legend continued on next page)

differing contributions to movement control, where DN/IPN likely convey motor timing signals via thalamus to cortex in order to initiate and modify ongoing movements (Kurata, 2005; Nashef et al., 2018; Thach, 2014).

In rodents, cerebellar nuclei project to different regions of ventral thalamus. The fastigial nucleus primarily targets ventromedial (VM), while DN/IPN target the anteromedial (AM), and ventral anterolateral (VAL) subdivisions (Angaut et al., 1985; Gornati et al., 2018; Haroian et al., 1981; Kuramoto et al., 2009; Teune et al., 2000). DN/IPN axon terminal fields overlap substantially displaying morphological and functional characteristics consistent with strong feedforward driver inputs, such as large synaptic boutons (Aumann and Horne, 1996a, b; Aumann et al., 1994; Gornati et al., 2018) and large unitary responses (Gornati et al., 2018; Sawyer et al., 1994; Schäfer et al., 2021). Cerebellar input drives short-latency spiking in thalamic neurons that project to superficial and deep layers of motor cortex (Hooks et al., 2013; Kuramoto et al., 2009; Schäfer et al., 2021), transforming output via top-down excitation through layer 2/3 (Weiler et al., 2008) or direct excitation of layer 5 (Hooks et al., 2013; Sauerbrei et al., 2020). Key remaining questions are whether ventral motor thalamus plays a role in movement initiation and whether this is dependent on cerebellar input.

To address these questions, we developed a cued lever push task for mice requiring execution of a basic stimulus-response behavior for reward. This habitual behavior depends on antecedent stimuli rather than goal value, likely recruiting feedback reinforcement circuits, including VAL thalamus (Balleine, 2019; Graybiel, 2008). Using imaging, electrophysiology, and gain- and loss-of-function experiments, we investigated how an auditory go cue transforms thalamic and motor cortical activity patterns during movement initiation. Population responses in DN/IPN-recipient regions of motor thalamus were dominated by a time-locked increase in activity immediately prior to movement initiation, providing a fixed-latency feedforward timing signal to motor cortex. Consistent with this view, membrane potential dynamics of layer 5B projection neurons matched premovement timing of thalamic activation, while suppressing cerebellar or thalamic output blocked movement initiation. Conversely, photostimulation of DN/IPN or recipient thalamic regions triggered movement initiation, but in a context-dependent manner. Our results demonstrate an important and causal contribution of a cerebellar-thalamocortical pathway to voluntary movement initiation.

RESULTS

Motor thalamic population activity increases prior to movement initiation

To investigate voluntary movement initiation, we developed a cued forelimb push task for mice. The design of the task required mice to execute horizontal push movements (4 mm) after a randomized inter-trial interval (4–6 s) and in response to a 6-kHz auditory go cue. Miss trials, partial pushes, or spontaneous lever movements resulted in no reward and a lever reset (Figure 1A; Video S1). Mice rapidly learned the task (mean = 7.5 days [6.3, 8.6] 95% confidence interval [CI], N = 16 mice; all data unless otherwise stated are presented as mean [bootstrapped 95% CI]; percentage of successful trials [last session], mean = 63.7% [56.0, 71.7]), displaying moderate reaction times (RTs; last session median = 0.32 s [0.30, 0.34]) and reproducible push trajectories (Figures 1B–1E; Video S1). Even in expert mice, we observed miss trials, likely reflecting changing levels of attention or satiation within sessions (Figure 1E; Video S1).

Since both DN and IPN are implicated in motor timing and send glutamatergic projections to ventral motor thalamus (Aumann and Horne, 1996b; Bosch-Bouju et al., 2013; Gornati et al., 2018; Kuramoto et al., 2009), we sought to define the region of thalamus that receives input from DN/IPN and projects to the caudal forelimb area (CFA) of motor cortex using a dual labeling strategy (Figure 1F). A region of dense overlap centered on VAL and AM nuclei, with sparse colocalization in the ventral posteromedial nucleus (VPM). We found no overlap in the ventromedial nucleus (VM), which primarily receives input from the fastigial nucleus (Gao et al., 2018; Gornati et al., 2018) (Figures 1G and S1A–S1E). Although injections were targeted to DN/IPN, low-level expression was detected in some adjacent vestibular nuclei, which do not send direct projections to VAL (Figures S1A and S1C). Within the dense region of overlap, ∼76% of neurons project to CFA, and all CFA-projecting neurons received glutamatergic input (vesicular glutamate transporter 2 [VGluT2] positive) from DN/IPN (Bosch-Bouju et al., 2013; Kuramoto et al., 2009; Rovó et al., 2012; Schäfer et al., 2020) (Figures 1H and S2A–S2D). This degree of connectivity is consistent with DN/IPN-recipient regions of motor thalamus ($MTh_{DN/IPN}$) being an important functional node connecting the cerebellum and CFA.

To explore whether $MTh_{DN/IPN}$ population responses were consistent with a role in movement initiation, we employed gradient-index (GRIN) lens-mediated two-photon population calcium imaging (Figures 1I and 1J). Lens implantation above $MTh_{DN/IPN}$ did not affect overall behavior when compared to control (control versus GRIN lens-implanted mice: two-sample Kolmogorov-Smirnov test, RT p = 0.56, push duration p = 0.22, number of successful pushes p = 0.35, N = 23 control versus 9 GRIN lens-implanted mice, data not shown). Most $MTh_{DN/IPN}$ neurons displayed push-related activity (210/248 neurons) either prior to movement initiation (early-onset increase in $\Delta F/F_0$, 104/210 neurons; early-onset decrease in $\Delta F/F_0$, 32/210 neurons) or during the execution/reward period (late-onset increase in $\Delta F/F_0$, 47/210 neurons; late-onset decrease in $\Delta F/F_0$, 27/210 neurons), while during miss trials, $MTh_{DN/IPN}$ population responses were absent (11 fields of view [FOVs], N = 8 mice) (Figures 1K–1L). Increased activity appeared as the dominant population response prior to movement (early-onset neurons: increased

(L) Average $\Delta F/F_0$ across trials for individual neurons. Groupings: early-onset increase (dark green, n = 104/248 neurons); early-onset decrease (light green, n = 32/248 neurons); late-onset increase (dark green hatching, n = 47/248 neurons); late-onset decrease (light green hatching, n = 27/248 neurons), and nonresponsive (gray, n = 38/248 neurons), ordered by $\Delta F/F_0$ onset, purple circles (n = 11 fields of view, N = 8 mice).

(M) Spatial distribution of early-onset increase (dark green), early-onset decrease (light green) and late-onset/nonresponsive neurons (gray) in $MTh_{DN/IPN}$. Dotted boxes, individual fields of view. ML, medial-lateral; AP anterior-posterior.

CellPress
OPEN ACCESS

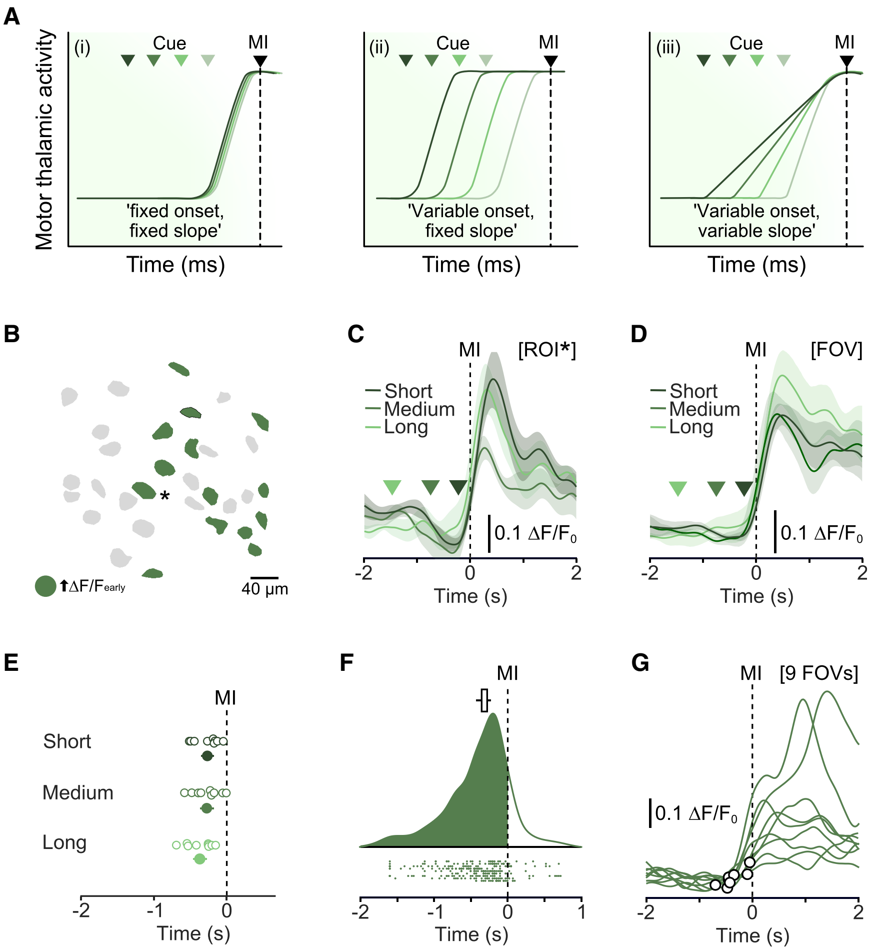

Figure 2. $MTh_{DN/IPN}$ neurons provide a reliable time-locked signal prior to movement initiation

(A) Trial-to-trial $MTh_{DN/IPN}$ population response models. Green triangles, cue onset; MI, movement initiation.

(B) Example field of view (FOV). Green, early-onset increase in activity.

(C) Average $\Delta F/F_0$ from an early-onset increase neuron (asterisk in B), aligned to movement initiation (MI) and split by short, medium, and long reaction times. Colored triangles, median cue presentation; [ROI], region of interest. Error bars represent 95% CIs.

(D) Average $\Delta F/F_0$ from all early-onset increase activity neurons in (B) FOV, aligned to movement initiation (MI) and split by reaction time. Triangles, median cue presentation. Error bars represent 95% CIs.

(E) Mean onset times of trials split by reaction time. Open circles, individual FOVs; filled circles, means ± 95% CI (n = 9 fields of view, N = 6 mice).

(F) Distribution of bootstrapped trial-to-trial response onsets for all early-onset increased activity neurons across nine FOVs. Top: median onset bootstrapped estimate. Middle: kernel density estimation of trial-to-trial motor thalamic response onsets. Bottom: raster of trial-to-trial population onsets (n = 297 trials from nine fields of view, N = 6 mice).

(G) Single-trial $\Delta F/F_0$ population responses from nine different FOVs (one response per FOV). Black circles, population response onsets.

activity, 76.4%; decreased activity, 23.5%) (Figure 1L) and was found across the extent of $MTh_{DN/IPN}$ (Figure 1M).

$MTh_{DN/IPN}$ output provides a reliable fixed-latency motor timing signal

If $MTh_{DN/IPN}$ conveys a motor timing signal, then population responses could be described by three hypothetical models. First, thalamic activity is uncoupled from the go cue rising immediately before movement onset. In this regard, rapidly increasing thalamic activity dictates the time of movement initiation (model i). Second, thalamic activity rises at the go cue and is maintained until additional inputs trigger movement. Thus, thalamic activity contributes to, but does not dictate, the time of initiation (model ii). Third, thalamic activity reflects a continuous sensorimotor transformation from cue to movement. The slope dictates the time of movement initiation (model iii) (Figure 2A). To distinguish between these models, we grouped trials by short, medium, and long RTs and aligned trial-averaged $\Delta F/F_0$ responses to movement initiation, focusing on early increased activity as this was the dominant population response (Figure 2B). Changes in $\Delta F/F_0$ occurred immediately prior to movement initiation, irrespective of RT (median onsets: short RT, −267 ms [−361, −178] 95% CI; medium RT, −276 ms [−374, −177] 95% CI; long RT, −367 ms [−464, −271] 95% CI, n = 104 neurons/9 FOVs, N = 6 mice, p = 0.46, one-way ANOVA). During medium and long RTs, baseline $\Delta F/F_0$ was maintained upon cue presentation, rising immediately before movement (Figures 2C and 2D). Response profiles were consistent trial to trial and across mice, indicative of a reliable motor timing signal that is temporally uncoupled from the auditory go cue (i.e., model i) (Figures 2E–2G).

Early-onset activity in CFA correlates with $MTh_{DN/IPN}$ response timing

In rodents, projections from VAL thalamus target deep layers of motor cortex (Hooks et al., 2013; Kuramoto et al., 2009). This feedforward glutamatergic input provides monosynaptic excitation and disynaptic inhibition to CFA principal neurons (Apicella et al., 2012; Hooks et al., 2013), shaping cortical output and behavior (Hooks et al., 2013; Kuramoto et al., 2009; Sauerbrei et al., 2020; Schiemann et al., 2015; Tanaka et al., 2018) (Figure 3A). We reasoned that if the $MTh_{DN/IPN}$ thalamocortical pathway conveys a pre-movement motor timing signal, then this should be reflected in the subthreshold membrane potential (V_m) dynamics of CFA layer 5 pyramidal neurons. We confirmed that layer 5 neurons receive direct input from $MTh_{DN/IPN}$ using monosynaptic retrograde rabies tracing in Rbp4-Cre transgenic mice (Gerfen et al., 2013; Kuramoto et al., 2009) (Figure S3A)

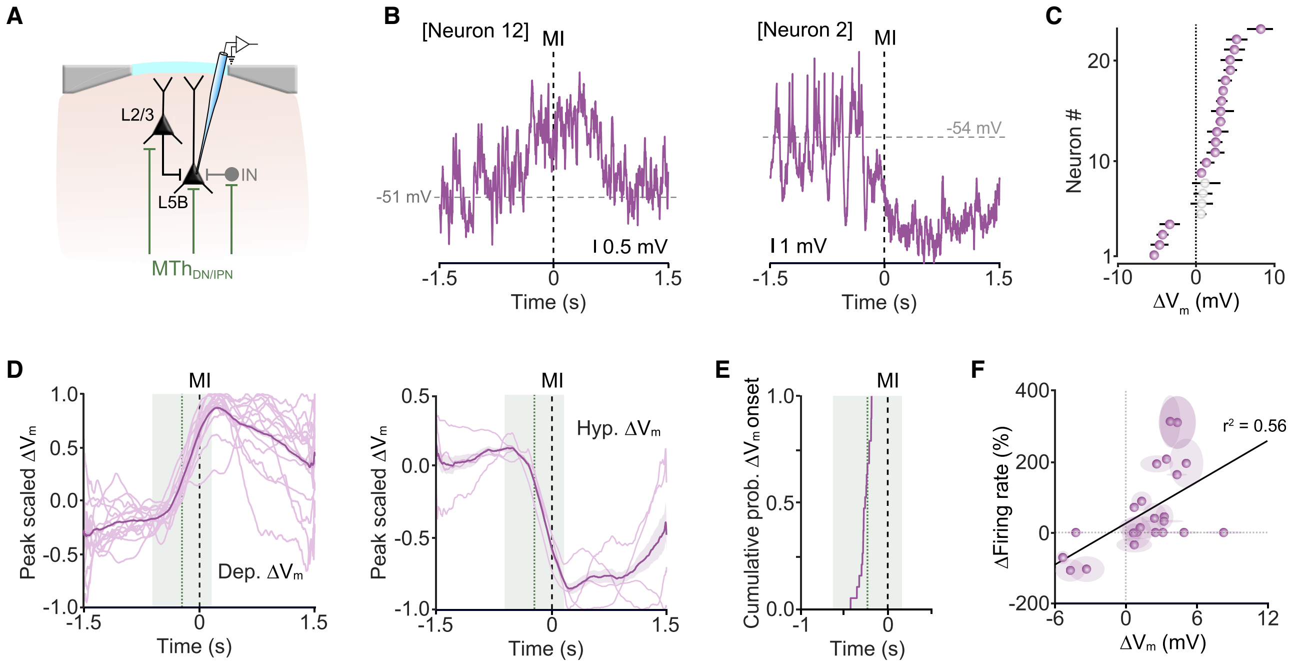

Figure 3. Early-onset changes in CFA layer 5B membrane potential dynamics

(A) Patch-clamp recording in layer5B CFA. IN, interneuron; $MTh_{DN/IPN}$, dentate/interpositus nucleus-recipient region of motor thalamus.

(B) Single-trial subthreshold membrane potential (V_m) trajectories from two layer 5B projection neurons (spikes clipped). MI, movement initiation.

(C) Peri-movement ΔV_m ± 95% CI. Purple and white indicate significant and non-significant changes in ΔV_m, respectively (n = 23 neurons from N = 23 mice).

(D) Overlaid peak scaled subthreshold V_m split by direction of change (left: depolarizing, n = 15/23 neurons; right: hyperpolarizing, n = 4/23 neurons). Thick line, population mean ± 95% CI; green dotted line, mean $MTh_{DN/IPN}$ activity onset ± 95% CI (green shading) shown in Figure 2E; black dashed line, movement initiation (MI).

(E) Cumulative probability of ΔV_m onsets (n = 19/23 neurons).

(F) Movement-related subthreshold ΔV_m and firing rate change correlation. Symbols, mean ± 95% CI from individual neurons; black line, linear fit to the data (Pearson's *r*).

before performing patch-clamp recordings (Figures 3A and S3B). When aligned to push onset, neurons displayed a rapid change in subthreshold activity, either depolarizing or hyperpolarizing, prior to movement initiation (depolarizing n = 15/23 neurons; hyperpolarizing, n = 4/23 neurons; nonresponsive, n = 4/23 neurons, N = 23 mice), with the direction of change being consistent trial to trial (Figures 3B and 3C). The timing of membrane potential changes (ΔV_m) in layer 5B neurons closely matched $MTh_{DN/IPN}$ population onsets (Figures 3D and 3E), consistent with direct feedforward modulation. Subthreshold V_m changes linearly correlated with firing rate in both intratelencephalic (IT-type) and pyramidal tract (PT-type) neurons that send projections to subcortical, brainstem, and spinal cord areas necessary for movement execution (Esposito et al., 2014; Kita and Kita, 2012; Park et al., 2021; Shepherd, 2013) (Figures 3F and S3C–S3J). During miss trials, ΔV_m was reduced, but not abolished, likely reflecting a lack of input from $MTh_{DN/IPN}$ (see Figure 1K), but maintained behavior-related inputs from other brain areas (Hooks et al., 2013) (Figure S3K).

Inactivating DN/IPN or $MTh_{DN/IPN}$ blocks movement initiation

To test whether the DN/IPN thalamocortical pathway is necessary for movement initiation, we performed loss-of-function experiments by focally injecting a small bolus of the $GABA_A$ receptor antagonist muscimol centered on DN/IPN, $MTh_{DN/IPN}$, or CFA (Figures 4A and S4A–S4C). Injecting muscimol during task execution allowed the immediate effects to be recorded 10 min after injection, restricting diffusion beyond targeted regions. Mapping the spread of fluorescent muscimol (see STAR Methods) indicated limited spread (~600 μm radius from the point of injection after 10 min) and localized inactivation of targeted nuclei (Figures 4B, 4C, and S4A–S4C). Our cortical injection strategy targeted all layers of CFA (spread diameter: anterior-posterior [AP], 1,240 ± 28.3 [SD] μm; mediolateral [ML], 1,133.2 ± 35.7 [SD] μm, N = 3 mice), without spreading to other cortical and subcortical areas (Figure S4A); similar results were found with DN/IPN injections (spread diameter: AP, 820 ± 89.4 [SD] μm; ML, 1,221.2 ± 265.4 [SD] μm, N = 4 mice) (Figure S4B). In ventral thalamus, spread was confined to $MTh_{DN/IPN}$, with minimal overlap in VM (spread diameter: AP, 960 ± 73.5 [SD] μm; ML, 957.5 ± 34.9 [SD] μm, N = 4 mice) (Figure S4C). Mapping muscimol diffusion using silicon probe recordings *in vivo* further confirmed limited spread beyond 600 μm 10 min after injection (Figure S4D), consistent with previously published estimates (Allen et al., 2008; Krupa et al., 1999; Martin, 1991). Inactivation of each node along the DN/IPN thalamocortical pathway significantly reduced the number of successful push trials (normalized number successful trials

CellPress
OPEN ACCESS

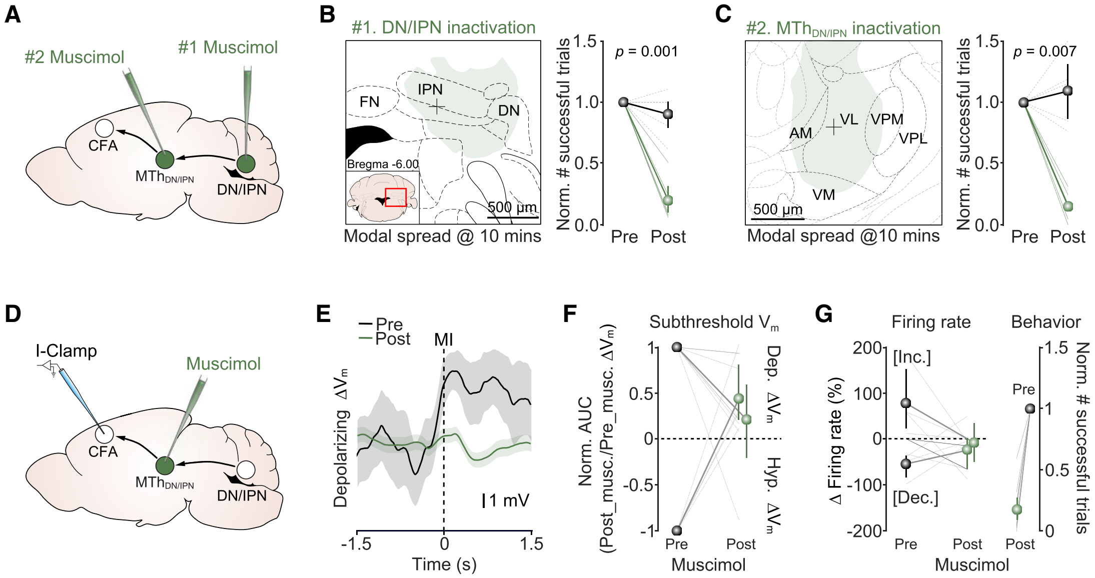

Figure 4. Activity in DN/IPN and $MTh_{DN/IPN}$ is required for cue-triggered movement initiation

(A) Muscimol inactivation of $MTh_{DN/IPN}$ or DN/IPN.

(B) Left: modal spread of fluorescent muscimol 10 min after injection into DN/IPN (inset, location of DN/IPN). Black cross, median point of IPN injection (N = 4 mice). Right: number of successful push trials 10 min after injection of saline (black, N = 6 mice) or muscimol (green, N = 6 mice), two-sample t test. Symbols, population means ± 95% CI. FN, fastigial nucleus; IPN, interpositus nucleus; DN, dentate nucleus.

(C) Left: modal spread of fluorescent muscimol 10 min after injection into $MTh_{DN/IPN}$ (inset, location of thalamic nuclei). Black cross, median point of injection (N = 4 mice). Right: number of successful push trials 10 min after injection of saline (black, N = 5 mice) or muscimol (green, N = 5 mice), two-sample t test. Symbols, population means ± 95% CI. AM, anteromedial; VL, ventrolateral; VPM, ventral posteromedial; VPL, ventral posterolateral; VM, ventromedial nuclei.

(D) Patch-clamp recording in layer 5B CFA during muscimol inactivation of $MTh_{DN/IPN}$. I-Clamp, current clamp.

(E) Subthreshold ΔV_m ± 95% CI from a layer 5B projection neuron before (Pre; black) and after muscimol injection (Post; green) targeted to $MTh_{DN/IPN}$.

(F) Ratio of normalized area under the curve for V_m trajectories before (Pre) and after (Post) muscimol injection into $MTh_{DN/IPN}$. Data grouped by V_m change prior to muscimol injection. Green symbols, population means ± 95% CI (n = 10 cells from N = 10 mice).

(G) Left: change in firing rate before (Pre) and after (Post) muscimol injection into $MTh_{DN/IPN}$. Colored symbols, population means ± 95% CI; dotted lines, individual neurons (n = 8 cells from N = 8 mice); black lines, neurons with no change in firing rate prior to muscimol injection (n = 2 cells from N = 2 mice). Right: number of successful push trials 10 mins after injection of muscimol (green, N = 10 mice).

post muscimol: DN/IPN, 0.20 [0.10, 0.34], p = 0.0013; $MTh_{DN/IPN}$, 0.15 [0.05, 0.25], p = 0.007; CFA, 0.19 [0.08, 0.30] 95% CI, p = 0.025, N = 6, 5, and 5, respectively, two-sample t test; comparison of effect size across manipulations: p = 0.85, one-way ANOVA with Tukey-Kramer post hoc test) due to an increase in miss trials rather than incomplete lever pushes (Figures 4B, 4C, and S4E–S4G; Video S2). Miss trials did not result from task disengagement, as the go cue reproducibly evoked short-latency whisking and increased arousal (see Video S2). Silencing DN/IPN and CFA reduced paw position accuracy in some trials (i.e., the forepaw was not placed on the lever), indicative of a role in controlling posture and movement initiation, while inactivating $MTh_{DN/IPN}$ selectively blocked movement initiation with no effect on paw placement accuracy (Figures S4E–S4G; Video S2).

To better understand how $MTh_{DN/IPN}$ output shapes cortical activity and behavior, we performed patch-clamp recordings of CFA layer 5B projection neurons while inactivating $MTh_{DN/IPN}$ (Figure 4D). Comparing the integral of subthreshold ΔV_m before and after silencing highlighted a reduction in ΔV_m magnitude irrespective of whether responses were depolarizing or hyperpolarizing. On average, neurons displaying depolarizing ΔV_m were reduced by ~80% (normalized area under the curve [AUC] after muscimol, 0.22 [−0.27, 0.64] 95% CI, p = 0.03, two-sample t test, N = 6 mice), while hyperpolarizing responses switched polarity to become moderately depolarizing (normalized AUC after muscimol, 0.44 [0.17, 0.85] 95% CI, p = 0.006, two-sample t test, N = 4 mice) (Figures 4E and 4F). Residual ΔV_m changes likely reflect convergence of other long-range inputs conveying task-related information (Guo et al., 2018; Hooks et al., 2013) (see Figures 1K and S3K), which combine with $MTh_{DN/IPN}$ input to trigger movement. As expected, blocking $MTh_{DN/IPN}$ activity reduced layer 5B firing rate changes and the number of successful push trials (Figure 4G).

Photoactivation of DN/IPN or $MTh_{DN/IPN}$ mimics go cue-evoked movement initiation

Although our loss-of-function experiments suggest the DN/IPN thalamocortical pathway is necessary for movement initiation, cerebellar and thalamic nuclei send projections to multiple brain regions involved in motor control (Asanuma et al., 1983; Hunnicutt et al., 2014; Kuramoto et al., 2009; Teune et al., 2000); therefore, we next tested whether stimulating $MTh_{DN/IPN}$ input to CFA or cerebellar input to $MTh_{DN/IPN}$ triggered movement. We reasoned that if the DN/IPN thalamocortical pathway conveys a movement timing signal, then photoactivation should mimic the effects of the go cue. To stimulate $MTh_{DN/IPN}$, we injected AAV-ChR2 unilaterally, centered on $MTh_{DN/IPN}$,

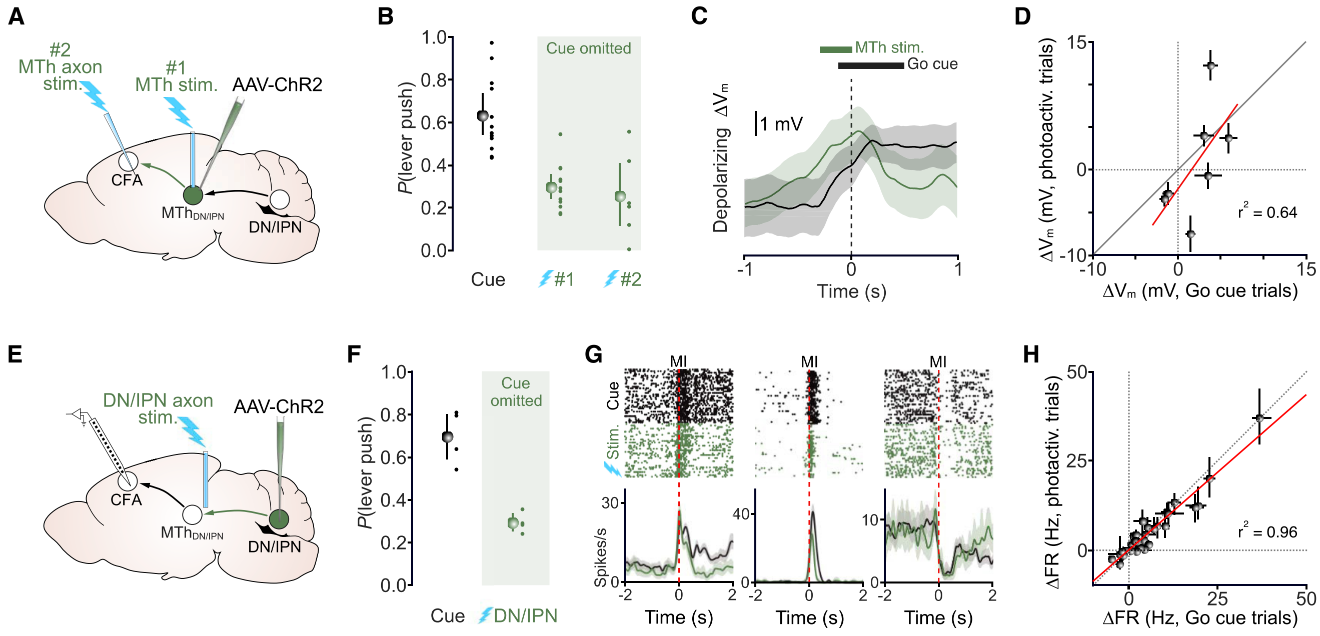

Figure 5. Stimulation of DN/IPN or $MTh_{DN/IPN}$ axon terminals triggers movement initiation

(A) Dual MTh photoactivation strategy; ChR2 expression targeted to $MTh_{DN/IPN}$, stimulation via optic fiber directly above $MTh_{DN/IPN}$ (#1) or tapered fiber in CFA (#2).

(B) Full lever push probability during an auditory go cue (black) or photoactivation of $MTh_{DN/IPN}$ (#1) or $MTh_{DN/IPN}$ axons in CFA (#2) in the absence of a go cue (green). Colored dots, individual mice; colored circles, mean ± 95% CI (cue, 1 and 2; N = 12, 12, and 6 mice, respectively).

(C) Change in subthreshold V_m ± 95% CI in a layer 5B projection neuron during the go cue (black) or photoactivation of $MTh_{DN/IPN}$ (green) in the absence of a cue. Dashed line, movement initiation.

(D) Peri-movement cue-evoked and photoactivated subthreshold ΔV_m correlation during push trials (n = 7 neurons, N = 6 mice). Filled symbols, mean ± 95% CI; red line, linear fit to the data (Pearson's *r*).

(E) Recording and photoactivation strategy: silicone probe recordings of deep-layer putative pyramidal cells in CFA during photoactivation of ChR2-expressing DN/IPN axon terminals in $MTh_{DN/IPN}$.

(F) Full lever push probability during an auditory go cue or photoactivation of DN/IPN axon terminals in $MTh_{DN/IPN}$ in the absence of a go cue. Colored dots, individual mice; colored circles, mean ± 95% CI (N = 4 and 4 mice).

(G) Spike rasters and peri-stimulus time histograms (PSTHs) from deep-layer CFA neurons aligned to movement initiation (dashed line). Black, cue trials; green, photoactivation trials.

(H) Peri-movement cue-evoked and photoactivated Δfiring rate correlation during push trials (n = 30/47 neurons, N = 4 mice). Symbols, mean ± 95% CI; red line, linear fit to the data (Pearson's *r*).

chronically implanted an optic fiber directly above thalamus, and acutely inserted a tapered optic fiber directly into CFA (Figures 5A and S5A). ChR2 expression was restricted to the center of $MTh_{DN/IPN}$ (i.e., VAL thalamic nuclei) with minimal off-target expression (Figures S5B and S5C). Direct stimulation of $MTh_{DN/IPN}$ or axon terminals in CFA in the absence of an auditory cue-triggered full lever push movements in ~30% of trials (go cue, lever push probability: *P*(lever push) 0.63 [0.53, 0.73] 95% CI; direct $MTh_{DN/IPN}$ stimulation, *P*(lever push) 0.29 [0.24, 0.35] 95% CI; axon terminal stimulation, *P*(lever push) 0.25 [0.11, 0.40] 95% CI) and a small proportion of partial lever pushes (N = 12 mice) (Figures 5B, S5D, and S5E; Video S3). RTs and duration of photoactivated push movements were comparable to cue-evoked trials (Figures S5F and S5G), while stimulating in the absence of ChR2 expression did not evoke any detectable forelimb movements (go cue, *P*(lever push) 0.95 [0.89, 1.00] 95% CI; direct $MTh_{DN/IPN}$ stimulation, *P*(lever push) 0.03 [0.00, 0.07] 95% CI, N = 2 mice) (data not shown). To compare cortical activity during go cue and photoactivation trials, we performed patch-clamp recordings from CFA layer 5B projection neurons. Go cue- and photoactivation-evoked ΔV_m were remarkably similar, both in the timing and direction of change, suggesting recruitment of the same inputs to CFA (Figures 5C, 5D, and S5H). Since stimulation of the ventral thalamus, including VM and VAL, has been shown to trigger short-latency licking (Catanese and Jaeger, 2021; Inagaki et al., 2020), we investigated whether $MTh_{DN/IPN}$ acts as a convergence hub coordinating motor timing signals necessary for triggering both tongue and forelimb movements. However, photoactivation of $MTh_{DN/IPN}$ rarely evoked short-latency licking or orofacial movements similar to those observed during a tactile delayed-response licking task (cue, *P*(lick): 0.77 [0.67, 0.87] 95% CI; photoactivation *P*(lick): 0.04 [0.00, 0.10] 95% CI, N = 12 mice, $p = 4.1 \times 10^{-11}$, two-sample t test) (Figure S5I) (Catanese and Jaeger, 2021; Guo et al., 2014; Inagaki et al., 2020). The selective triggering of forelimb push movements in our behavior suggests parallel but distinct thalamocortical pathways for tongue and limb movements.

CellPress
OPEN ACCESS

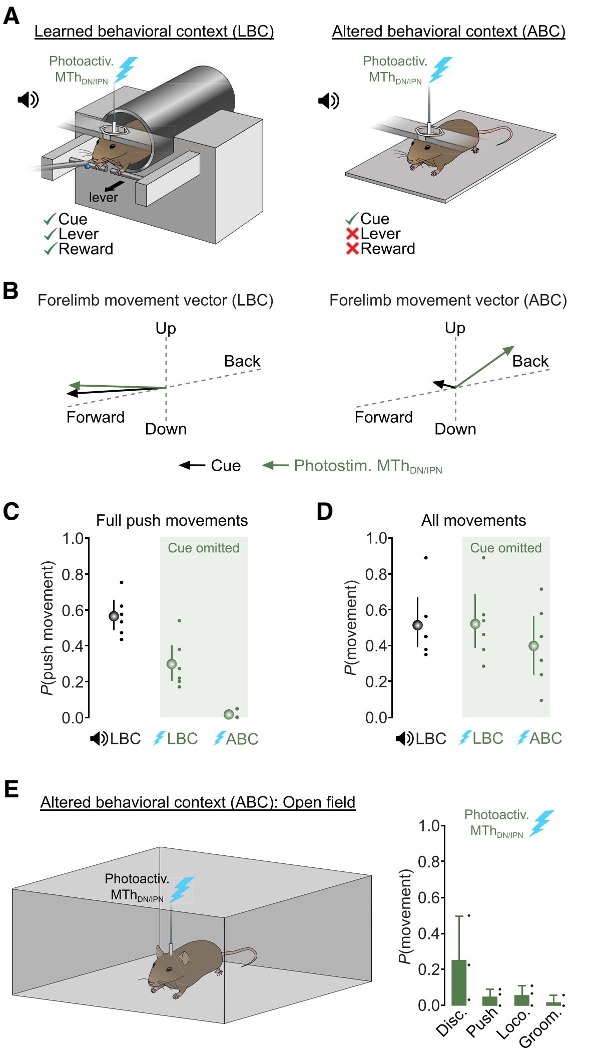

Figure 6. Photoactivation of $MTh_{DN/IPN}$ evokes behavioral context-dependent movement initiation

(A) $MTh_{DN/IPN}$ photoactivation in a learned behavioral context (LBC; left) or altered behavioral context (ABC; right).

(B) Average forelimb movement vectors from an example mouse during an auditory go cue (black arrows) or photoactivation of $MTh_{DN/IPN}$ in the absence of a cue (green arrows). Arrow length, scaled by total number of across trial movements.

(C) Push or push-like movement probability during an auditory go cue (black) or photoactivation of $MTh_{DN/IPN}$ in the absence of a go cue (green). Dots, individual mice; symbols, mean ± 95% CI (N = 6 mice).

(D) Forelimb movement probability during an auditory go cue (black) or photoactivation of $MTh_{DN/IPN}$ in the absence of a go cue (green). Mean ± 95% CI (N = 6 mice).

(E) Forelimb movement probability evoked by photoactivation of $MTh_{DN/IPN}$ in an open-field environment (N = 3 mice). Disc., discrete movements; Push, push-like movements; Loco., locomotion; Groom, grooming behavior. Bars, mean ± 95% CI; dots, individual mice.

We next investigated whether cerebellar input to $MTh_{DN/IPN}$ can initiate movement by targeting ChR2 expression to DN/IPN and stimulating axons terminals in $MTh_{DN/IPN}$ (Figures 5E and S5J–S5O). ChR2 expression was restricted to DN/IPN with minimal or no expression in surrounding nuclei (Figures S5J, S5K, and S5O). Photoactivation in the absence of the auditory go cue-triggered full lever push movements in ~30% of trials (go cue, *P*(lever push) 0.70 [0.59, 0.80] 95% CI; DN/IPN axon terminal stimulation, *P*(lever push) 0.29 [0.25, 0.33] 95% CI, N = 4 mice), similar to direct $MTh_{DN/IPN}$ stimulation (Figure 5F; Video S4). To investigate whether overlapping populations of CFA neurons were recruited during go cue and photoactivation trials, we used silicon probe recordings, focusing on a subset of deep-layer putative pyramidal neurons that were movement responsive (n = 47/216 neurons; N = 4 mice) (Figures S5P–S5U). Responses in individual neurons were highly consistent trial to trial, with movement-related activity patterns varying widely across the population (Figures 5G and S5U), consistent with our ground truth intracellular data (see Figures 3A–3C). A large proportion of cue-responsive neurons also displayed significant responses during photoactivation trials (30/47 neurons, 63.8%, from N = 4 mice), irrespective of the direction of firing rate change (Figures 5G, 5H, and S5U), suggesting photoactivation of the DN/IPN thalamocortical pathway mimics cue-evoked activity patterns in CFA.

$MTh_{DN/IPN}$ stimulation triggers behavioral context-dependent movement initiation

Presentation of a go cue or photoactivation of $MTh_{DN/IPN}$ triggers lever pushes in a learned behavioral context (LBC). But whether push movements would be generated in an altered behavioral context (ABC) is unclear. If thalamocortical stimulation alone is sufficient to trigger the learned behavior, then photoactivation in an ABC should still generate "push-like" movements. However, if $MTh_{DN/IPN}$ simply conveys a motor timing signal that combines with behavioral context-dependent inputs from other brain areas, then photoactivation of $MTh_{DN/IPN}$ in an ABC will likely trigger movement, but not learned push movements. To address this, we designed an ABC, which consisted of a flat baseplate in the absence of any support/movable levers, reward spout, or water reward and compared cue- and $MTh_{DN/IPN}$-photoactivation-evoked movements across contexts (LBC versus ABC) (Figure 6A). Mice were first trained in the LBC before being habituated to the ABC within and across training sessions to ensure cued lever push movements were not extinguished in the LBC. As expected, trained mice generated cue-evoked lever pushes in 56% of trials in the LBC but very few push movements in the ABC (LBC, *P*(push movement) = 0.56 [0.49, 0.65] 95% CI; ABC, *P*(push movement) = 0.01 [0.00, 0.03] 95% CI, $p = 3.9 \times 10^{-7}$, two-sample t test, N = 6 mice), confirming that mice acknowledged the difference between the two behavioral contexts (Figures 6B–6D and S6; Video S5). Direct photoactivation of $MTh_{DN/IPN}$ in the LBC, and in the absence of a cue, evoked forelimb movements in 52% of trials, where 30% of trials were successful lever pushes (LBC, *P*(movement) = 0.52 [0.39, 0.69] 95% CI; LBC, *P*(push movement) = 0.30 [0.21, 0.41] 95% CI, N = 6 mice). While in the ABC, direct $MTh_{DN/IPN}$ stimulation reliably evoked forelimb movements in 40% of trials but only 2% contained "push-like" movements (ABC, *P*(movement) = 0.40 [0.24, 0.56] 95% CI; ABC, *P*(push movement) = 0.02 [0.00, 0.03] 95% CI, $p = 1.9 \times 10^{-3}$, two-sample t test, N = 6 mice) (Figures 6B–6D and S6; Video S5). The absence of push-like movements could result from differences in posture; however,

photostimulated movements in mice mounted on a flat baseplate (ABC) or in a behavioral context that recapitulated the LBC mouse posture (ABC2) were not different (Figure S6). In addition, photostimulation of $MTh_{DN/IPN}$ in an open-field environment triggered discrete forelimb movements in ~25% of trials, consistent with a role in movement initiation, but very few push-like movements (N = 3 mice) (Figure 6E). Together, these data suggest that the DN/IPN thalamocortical pathway conveys motor timing signals that trigger behavioral context-dependent movement initiation.

DISCUSSION

The cerebellum and motor thalamus are brain areas thought to control movement timing, since activity in both regions precedes movement initiation (Anderson and Turner, 1991; Butler et al., 1992, 1996; Fortier et al., 1989; Harvey et al., 1979; Horne and Porter, 1980; Kurata, 2005; Macpherson et al., 1980; Mushiake and Strick, 1993; Schmied et al., 1979; Strick, 1976; van Donkelaar et al., 1999) and local inactivation disrupts motor timing (Meyer-Lohmann et al., 1977; Nashef et al., 2019; Spidalieri et al., 1983; Thach, 1975; van Donkelaar et al., 2000). Our anatomical mapping identified a high degree of connectivity between DN/IPN and CFA-projecting neurons in VAL, AM, and VPM. In rodents, VAL neurons receive strong driver-like inputs (Gornati et al., 2018) that facilitate rapid depolarization of thalamic projection neurons (Aumann and Horne, 1996a, 1996b; Aumann et al., 1994; Gao et al., 2018; Gornati et al., 2018; Sawyer et al., 1994; Schäfer et al., 2021). This driving input, when integrated with GABAergic input from the basal ganglia and thalamic reticular nucleus, shapes the magnitude and timing of thalamic excitability (Bosch-Bouju et al., 2013; Catanese and Jaeger, 2021; Kim et al., 2017; Kuramoto et al., 2011; Lam and Sherman, 2015; Sakai et al., 1998; Tanaka et al., 2018). Early-onset $MTh_{DN/IPN}$ activity was temporally uncoupled from the go cue but tightly locked to movement initiation, suggestive of a pure motor timing signal that indicates the intention to move rather than a sensorimotor transformation from cue to movement (see Figure 2). Consistent with this view, cue presentation during miss trials did not evoke a change in activity, likely reflecting a lack of intention to move and absence of direct auditory input in VAL thalamus, while suppressing $MTh_{DN/IPN}$ generated a selective block of forelimb movement initiation.

Although we focused on the DN/IPN thalamocortical pathway, projections from DN/IPN also target the ventral tegmental area, substantia nigra reticulata, brainstem reticular nucleus, and magnocellular red nucleus (Carta et al., 2019; Gornati et al., 2018; Houck and Person, 2015; Low et al., 2018; Sakayori et al., 2019; Sathyamurthy et al., 2020; Thanawalla et al., 2020). Direct projections to the brainstem provide an alternate pathway to initiate movement. We found that recruitment of the DN/IPN thalamocortical pathway is necessary for learned forelimb movement initiation given that photoactivation of DN/IPN axon terminals in $MTh_{DN/IPN}$ mimics cue-triggered CFA population dynamics and behavior, while silencing each node along the pathway blocked initiation. These observations differ from photomodulation of cerebellar output in locomoting mice, where stimulation initiates or modifies sequences of limb movements via descending projections to the brainstem (Hoogland et al., 2015; Jelitai et al., 2016; Witter et al., 2013). Direct photoactivation of $MTh_{DN/IPN}$ in an open-field environment triggered discrete, but not rhythmic, forelimb movements, suggesting selective recruitment of descending or thalamocortical pathways depending on movement type and behavioral context. In addition to a proposed role in movement initiation, DN/IPN contribute to the coordination of ongoing movements. IPN inactivation results in disrupted endpoint accuracy, hypermetria, and instability of the forelimb (Becker and Person, 2019; Bracha et al., 1999; Low et al., 2018; Martin et al., 2000; Mason et al., 1998), while DN inactivation increases path curvature and generates hypermetria and a general impairment in coordination (Ishikawa et al., 2014; Martin et al., 2000). The fact that DN/IPN inactivation reduced both paw position accuracy (i.e., the ability to maintain postural control) and movement initiation is consistent with a role in both motor timing and coordination.

Changes in $MTh_{DN/IPN}$ and CFA layer 5B neuron activity occurred prior to movement and peaked around movement completion, indicative of rapid preparatory activity that transforms into output dynamics necessary for execution (Lara et al., 2018). In rodents, rapid go cue-evoked changes in activity have been observed in a delayed directional licking task for mice (Catanese and Jaeger, 2021; Gao et al., 2018; Guo et al., 2014; Li et al., 2015), where input from the pedunculopontine nucleus, midbrain reticular nucleus, and substantia nigra reticulata, via ventral motor thalamus, triggers rapid reorganization of preparatory dynamics to initiate directional licking (Catanese and Jaeger, 2021; Inagaki et al., 2020). Thus, ventral thalamus appears ideally positioned to act as a central convergence hub, integrating input from the cerebellum, brainstem, and basal ganglia in order to initiate precisely timed movements. However, direct photoactivation of $MTh_{DN/IPN}$ did not reproducibly evoke short-latency tongue or orofacial movements, suggesting parallel, non-overlapping thalamocortical pathways for movement initiation. Directional licking requires channeling of information through VM, VAL, mediodorsal, and intralaminar nuclei for both movement planning and execution (Catanese and Jaeger, 2021; Inagaki et al., 2020), while forelimb movements require activity in VAL, AM, and VPM nuclei. Together, this suggests that parallel processing of motor timing signals through different ventral motor thalamic nuclei could provide an anatomical substrate for initiating complex, multifaceted motor behaviors.

Using monosynaptic rabies tracing, we confirmed a direct pathway from $MTh_{DN/IPN}$ to layer 5B projection neurons in CFA, consistent with the idea that VAL projects to both superficial and deep layers of motor cortex, while neurons in VM project primarily to L1 (Hooks et al., 2013; Kuramoto et al., 2009, 2015). Strong thalamic input generates monosynaptic excitation and disynaptic feedforward inhibition in principal neurons (Apicella et al., 2012; Hooks et al., 2013), shaping cortical output via top-down control or direct activation of output layers (Hooks et al., 2013; Sauerbrei et al., 2020; Weiler et al., 2008). Since photoactivation of the $MTh_{DN/IPN}$ thalamocortical pathway reproduced go-cue-evoked layer 5B neuronal dynamics, thalamic input may directly influence cortical output by bypassing top-down processing from layer 2/3 to inform PT-type and IT-type neurons of the intention to move (Hooks et al., 2013; Weiler

et al., 2008). This direct timing signal would be integrated with other long-range task-relevant inputs to generate specific output patterns necessary for forelimb motor control (Esposito et al., 2014; Kita and Kita, 2012; Park et al., 2021; Sauerbrei et al., 2020). Although we focused solely on the contribution of CFA, VAL also sends projections to the rostral forelimb area (RFA) (Hooks et al., 2013; Oh et al., 2014), which plays an integral role in movement coordination (Brown and Teskey, 2014; Morandell and Huber, 2017). Given its strong reciprocal connectivity with CFA (Hira et al., 2013; Mohammed and Jain, 2016; Rouiller et al., 1993), assessing the contribution of the VAL-RFA pathway to movement initiation will be an important next step.

Our behavioral context experiments further support $MTh_{DN/IPN}$ conveying a generic motor timing signal that converges, at the level of motor cortex, with other task-relevant inputs. In the absence of thalamic input to $MTh_{DN/IPN}$ (i.e., miss trials or MTh inactivation), layer 5B V_m and firing rate changes were significantly reduced, with residual V_m changes being insufficient to trigger movement, suggesting input convergence is a prerequisite for learned movement initiation. The origin of the additional input(s) remains unknown, but likely candidates are corticocortical interactions between frontal motor areas and CFA (Hooks et al., 2013; Reep et al., 1990), thought to accumulate task-relevant information required for motor planning and execution (Gao et al., 2018; Li et al., 2015), or basal ganglia thalamocortical interactions involved in selecting, timing, and invigorating different actions (Dudman and Krakauer, 2016; Inase et al., 1996; Klaus et al., 2019; Thura and Cisek, 2017; Williams and Herberg, 1987). Directly activating the $MTh_{DN/IPN}$ thalamocortical pathway in the LBC mimicked the go cue by triggering push movements, while in the ABC, photoactivation evoked highly variable forelimb trajectories. Why does photoactivation result in learned movement initiation in the absence of an external sensory cue? We suggest that the DN/IPN thalamocortical pathway provides one of the main driving inputs to CFA, which combines with other task-relevant inputs (e.g., behavioral context, stimulus-reward associations, and reward expectancy) to generate "learned" cortical output patterns and behavior. In the ABC, task-relevant inputs are likely absent, thus mimicking the thalamic "timing signal" is in itself sufficient to generate cortical output patterns necessary for movement (Tanaka et al., 2018), but not the learned movement.

In summary, our findings extend our understanding of how specific subdivisions of the motor thalamus contribute to motor timing, suggesting that the DN/IPN thalamocortical pathway plays a critical role in generating cortical dynamics necessary for context-dependent movement initiation.

STAR★METHODS

Detailed methods are provided in the online version of this paper and include the following:

- KEY RESOURCES TABLE
- RESOURCE AVAILABILITY
  - Lead contact
  - Materials availability
  - Data and code availability
- EXPERIMENTAL MODEL AND SUBJECT DETAILS
- METHOD DETAILS
  - General surgery
  - Behavioral training
  - *In vivo* pharmacology
  - GRIN lens imaging
  - Whole-cell patch-clamp electrophysiology
  - Immunohistochemistry
  - Motor thalamic activation
  - Cerebellar-motor thalamic pathway tracing and activation
  - Monosynaptic retrograde rabies tracing
  - Extracellular recording and spike sorting
  - Quantifying muscimol diffusion
  - Forelimb kinematic tracking
- QUANTIFICATION AND STATISTICAL ANALYSIS

SUPPLEMENTAL INFORMATION

Supplemental information can be found online at https://doi.org/10.1016/j.neuron.2021.05.016.

ACKNOWLEDGMENTS

We are grateful to T. Branco, B. Grewe, J. Gründemann, M. Nolan, G. Sürmeli, B. Mensh, and members of the Nolan, Sürmeli, and Duguid labs for experimental discussions and comments on the manuscript. We thank M. Zechner for graphic design. Rabies virus was a gift from E. Callaway (Salk Institute) to A.W.H. GCaMP6s was a gift from Douglas Kim and the GENIE Project (Addgene 100844-AAV1). ChR2-Venus-AAV was a gift from Karel Svoboda (Addgene plasmid #20071). This study was supported by the BBSRC (UK) (BB/R018537/1), DFG (Germany) (SCHI1267/2-1 to J.S. and AM 443/1-1 to J.J.A.), the Shirley Foundation (UK), HHMI (USA) (A.W.H. and C.-C.H.), and a Wellcome SRF (UK) (110131/Z/15/Z) to I.D.

AUTHOR CONTRIBUTIONS

Conceptualization, J.D., M.C., J.J.A., J.S., A.W.H., and I.D.; methodology & investigation, J.D., M.C., T.C., J.J.A., J.S., V.C.-P., F.C., J.A.H., and C.-C.H.; resources, J.P., and N.L.R.; writing – review & editing, all authors (see Table S1, contributions matrix).

DECLARATION OF INTERESTS

The authors declare no competing interests.

Received: September 23, 2019
Revised: April 7, 2021
Accepted: May 11, 2021
Published: June 18, 2021

REFERENCES

Allen, T.A., Narayanan, N.S., Kholodar-Smith, D.B., Zhao, Y., Laubach, M., and Brown, T.H. (2008). Imaging the spread of reversible brain inactivations using fluorescent muscimol. J. Neurosci. Methods *171*, 30–38.

Anderson, M.E., and Turner, R.S. (1991). Activity of neurons in cerebellar-receiving and pallidal-receiving areas of the thalamus of the behaving monkey. J. Neurophysiol. *66*, 879–893.

Angaut, P., Cicirata, F., and Serapide, F. (1985). Topographic organization of the cerebellothalamic projections in the rat. An autoradiographic study. Neuroscience *15*, 389–401.

Apicella, A.J., Wickersham, I.R., Seung, H.S., and Shepherd, G.M. (2012). Laminarly orthogonal excitation of fast-spiking and low-threshold-spiking interneurons in mouse motor cortex. J. Neurosci. *32*, 7021–7033.

Asanuma, C., Thach, W.T., and Jones, E.G. (1983). Brainstem and spinal projections of the deep cerebellar nuclei in the monkey, with observations on the brainstem projections of the dorsal column nuclei. Brain Res. *286*, 299–322.

Aumann, T.D., and Horne, M.K. (1996a). A comparison of the ultrastructure of synapses in the cerebello-rubral and cerebello-thalamic pathways in the rat. Neurosci. Lett. *211*, 175–178.

Aumann, T.D., and Horne, M.K. (1996b). Ramification and termination of single axons in the cerebellothalamic pathway of the rat. J. Comp. Neurol. *376*, 420–430.

Aumann, T.D., Rawson, J.A., Finkelstein, D.I., and Horne, M.K. (1994). Projections from the lateral and interposed cerebellar nuclei to the thalamus of the rat: a light and electron microscopic study using single and double anterograde labelling. J. Comp. Neurol. *349*, 165–181.

Balleine, B.W. (2019). The meaning of behavior: discriminating reflex and volition in the brain. Neuron *104*, 47–62.

Bastian, A.J., and Thach, W.T. (1995). Cerebellar outflow lesions: a comparison of movement deficits resulting from lesions at the levels of the cerebellum and thalamus. Ann. Neurol. *38*, 881–892.

Becker, M.I., and Person, A.L. (2019). Cerebellar Control of Reach Kinematics for Endpoint Precision. Neuron *103*, 335–348.e5.

Bosch-Bouju, C., Hyland, B.I., and Parr-Brownlie, L.C. (2013). Motor thalamus integration of cortical, cerebellar and basal ganglia information: implications for normal and parkinsonian conditions. Front. Comput. Neurosci. *7*, 163.

Bostan, A.C., Dum, R.P., and Strick, P.L. (2013). Cerebellar networks with the cerebral cortex and basal ganglia. Trends Cogn. Sci. *17*, 241–254.

Bracha, V., Kolb, F.P., Irwin, K.B., and Bloedel, J.R. (1999). Inactivation of interposed nuclei in the cat: classically conditioned withdrawal reflexes, voluntary limb movements and the action primitive hypothesis. Exp. Brain Res. *126*, 77–92.

Brown, A.R., and Teskey, G.C. (2014). Motor cortex is functionally organized as a set of spatially distinct representations for complex movements. J. Neurosci. *34*, 13574–13585.

Butler, E.G., Horne, M.K., and Hawkins, N.J. (1992). The activity of monkey thalamic and motor cortical neurones in a skilled, ballistic movement. J. Physiol. *445*, 25–48.

Butler, E.G., Finkelstein, D.I., Harvey, M.C., Churchward, P.R., Forlano, L.M., and Horne, M.K. (1996). The relationship between monkey ventrolateral thalamic nucleus activity and kinematic parameters of wrist movement. Brain Res. *736*, 146–159.

Carta, I., Chen, C.H., Schott, A.L., Dorizan, S., and Khodakhah, K. (2019). Cerebellar modulation of the reward circuitry and social behavior. Science *363*, eaav0581.

Catanese, J., and Jaeger, D. (2021). Premotor ramping of thalamic neuronal activity is modulated by nigral inputs and contributes to control the timing of action release. J. Neurosci. *41*, 1878–1891.

Chen, T.-W., Wardill, T.J., Sun, Y., Pulver, S.R., Renninger, S.L., Baohan, A., Schreiter, E.R., Kerr, R.A., Orger, M.B., Jayaraman, V., et al. (2013). Ultrasensitive fluorescent proteins for imaging neuronal activity. Nature *499*, 295–300.

Cooper, S.E., Martin, J.H., and Ghez, C. (2000). Effects of inactivation of the anterior interpositus nucleus on the kinematic and dynamic control of multijoint movement. J. Neurophysiol. *84*, 1988–2000.

Dudman, J.T., and Krakauer, J.W. (2016). The basal ganglia: from motor commands to the control of vigor. Curr. Opin. Neurobiol. *37*, 158–166.

Esposito, M.S., Capelli, P., and Arber, S. (2014). Brainstem nucleus MdV mediates skilled forelimb motor tasks. Nature *508*, 351–356.

Fortier, P.A., Kalaska, J.F., and Smith, A.M. (1989). Cerebellar neuronal activity related to whole-arm reaching movements in the monkey. J. Neurophysiol. *62*, 198–211.

Gao, Z., Davis, C., Thomas, A.M., Economo, M.N., Abrego, A.M., Svoboda, K., De Zeeuw, C.I., and Li, N. (2018). A cortico-cerebellar loop for motor planning. Nature *563*, 113–116.

Gerfen, C.R., Paletzki, R., and Heintz, N. (2013). GENSAT BAC cre-recombinase driver lines to study the functional organization of cerebral cortical and basal ganglia circuits. Neuron *80*, 1368–1383.

Gibson, A.R., Houk, J.C., and Kohlerman, N.J. (1985). Magnocellular red nucleus activity during different types of limb movement in the macaque monkey. J. Physiol. *358*, 527–549.

Gornati, S.V., Schäfer, C.B., Eelkman Rooda, O.H.J., Nigg, A.L., De Zeeuw, C.I., and Hoebeek, F.E. (2018). Differentiating cerebellar impact on thalamic nuclei. Cell Rep. *23*, 2690–2704.

Graybiel, A.M. (2008). Habits, rituals, and the evaluative brain. Annu. Rev. Neurosci. *31*, 359–387.

Guo, Z.V., Li, N., Huber, D., Ophir, E., Gutnisky, D., Ting, J.T., Feng, G., and Svoboda, K. (2014). Flow of cortical activity underlying a tactile decision in mice. Neuron *81*, 179–194.

Guo, K., Yamawaki, N., Svoboda, K., and Shepherd, G.M.G. (2018). Anterolateral motor cortex connects with a medial subdivision of ventromedial thalamus through cell type-specific circuits, forming an excitatory thalamo-cortico-thalamic loop via layer 1 apical tuft dendrites of layer 5B pyramidal tract type neurons. J. Neurosci. *38*, 8787–8797.

Haroian, A.J., Massopust, L.C., and Young, P.A. (1981). Cerebellothalamic projections in the rat: an autoradiographic and degeneration study. J. Comp. Neurol. *197*, 217–236.

Harvey, R.J., Porter, R., and Rawson, J.A. (1979). Discharges of intracerebellar nuclear cells in monkeys. J. Physiol. *297*, 559–580.

Hira, R., Ohkubo, F., Tanaka, Y.R., Masamizu, Y., Augustine, G.J., Kasai, H., and Matsuzaki, M. (2013). In vivo optogenetic tracing of functional corticocortical connections between motor forelimb areas. Front. Neural Circuits *7*, 55.

Holmes, G. (1939). The cerebellum of man. Brain *62*, 1–30.

Hoogland, T.M., De Gruijl, J.R., Witter, L., Canto, C.B., and De Zeeuw, C.I. (2015). Role of synchronous activation of cerebellar purkinje cell ensembles in multi-joint movement control. Curr. Biol. *25*, 1157–1165.

Hooks, B.M., Mao, T., Gutnisky, D.A., Yamawaki, N., Svoboda, K., and Shepherd, G.M. (2013). Organization of cortical and thalamic input to pyramidal neurons in mouse motor cortex. J. Neurosci. *33*, 748–760.

Horne, M.K., and Butler, E.G. (1995). The role of the cerebello-thalamo-cortical pathway in skilled movement. Prog. Neurobiol. *46*, 199–213.

Horne, M.K., and Porter, R. (1980). The discharges during movement of cells in the ventrolateral thalamus of the conscious monkey. J. Physiol. *304*, 349–372.

Houck, B.D., and Person, A.L. (2015). Cerebellar premotor output neurons collateralize to innervate the cerebellar cortex. J. Comp. Neurol. *523*, 2254–2271.

Huang, C.-C., Sugino, K., Shima, Y., Guo, C., Bai, S., Mensh, B.D., Nelson, S.B., and Hantman, A.W. (2013). Convergence of pontine and proprioceptive streams onto multimodal cerebellar granule cells. Elife *2*, e00400. https://doi.org/10.7554/eLife.00400.

Hunnicutt, B.J., Long, B.R., Kusefoglu, D., Gertz, K.J., Zhong, H., and Mao, T. (2014). A comprehensive thalamocortical projection map at the mesoscopic level. Nat. Neurosci. *17*, 1276–1285.

Inagaki, H.K., Chen, S., Ridder, M.C., Sah, P., Li, N., Yang, Z., Hasanbegovic, H., Gao, Z., Gerfen, C.R., and Svoboda, K. (2020). A midbrain-thalamus-cortex circuit reorganizes cortical dynamics to initiate planned movement. bioRxiv. https://doi.org/10.1101/2020.12.16.423127.

Inase, M., Buford, J.A., and Anderson, M.E. (1996). Changes in the control of arm position, movement, and thalamic discharge during local inactivation in the globus pallidus of the monkey. J. Neurophysiol. *75*, 1087–1104.

Ishikawa, T., Tomatsu, S., Tsunoda, Y., Lee, J., Hoffman, D.S., and Kakei, S. (2014). Releasing dentate nucleus cells from Purkinje cell inhibition generates output from the cerebrocerebellum. PLoS ONE *9*, e108774.

CellPress
OPEN ACCESS

Jelitai, M., Puggioni, P., Ishikawa, T., Rinaldi, A., and Duguid, I. (2016). Dendritic excitation-inhibition balance shapes cerebellar output during motor behaviour. Nat. Commun. *7*, 13722.

Jun, J.J., Steinmetz, N.A., Siegle, J.H., Denman, D.J., Bauza, M., Barbarits, B., Lee, A.K., Anastassiou, C.A., Andrei, A., Aydın, Ç., et al. (2017). Fully integrated silicon probes for high-density recording of neural activity. Nature *551*, 232–236.

Keemink, S.W., Lowe, S.C., Pakan, J.M.P., Dylda, E., van Rossum, M.C.W., and Rochefort, N.L. (2018). FISSA: A neuropil decontamination toolbox for calcium imaging signals. Sci. Rep. *8*, 3493.

Kim, J., Kim, Y., Nakajima, R., Shin, A., Jeong, M., Park, A.H., Jeong, Y., Jo, S., Yang, S., Park, H., et al. (2017). Inhibitory basal ganglia inputs induce excitatory motor signals in the thalamus. Neuron *95*, 1181–1196.e8.

Kita, T., and Kita, H. (2012). The subthalamic nucleus is one of multiple innervation sites for long-range corticofugal axons: a single-axon tracing study in the rat. J. Neurosci. *32*, 5990–5999.

Klaus, A., Alves da Silva, J., and Costa, R.M. (2019). What, if, and when to move: basal ganglia circuits and self-paced action initiation. Annu. Rev. Neurosci. *42*, 459–483.

Krupa, D.J., Ghazanfar, A.A., and Nicolelis, M.A. (1999). Immediate thalamic sensory plasticity depends on corticothalamic feedback. Proc. Natl. Acad. Sci. USA *96*, 8200–8205.

Kuramoto, E., Furuta, T., Nakamura, K.C., Unzai, T., Hioki, H., and Kaneko, T. (2009). Two types of thalamocortical projections from the motor thalamic nuclei of the rat: a single neuron-tracing study using viral vectors. Cereb. Cortex *19*, 2065–2077.

Kuramoto, E., Fujiyama, F., Nakamura, K.C., Tanaka, Y., Hioki, H., and Kaneko, T. (2011). Complementary distribution of glutamatergic cerebellar and GABAergic basal ganglia afferents to the rat motor thalamic nuclei. Eur. J. Neurosci. *33*, 95–109.

Kuramoto, E., Ohno, S., Furuta, T., Unzai, T., Tanaka, Y.R., Hioki, H., and Kaneko, T. (2015). Ventral medial nucleus neurons send thalamocortical afferents more widely and more preferentially to layer 1 than neurons of the ventral anterior-ventral lateral nuclear complex in the rat. Cereb. Cortex *25*, 221–235.

Kurata, K. (2005). Activity properties and location of neurons in the motor thalamus that project to the cortical motor areas in monkeys. J. Neurophysiol. *94*, 550–566.

Lam, Y.W., and Sherman, S.M. (2015). Functional topographic organization of the motor reticulothalamic pathway. J. Neurophysiol. *113*, 3090–3097.

Lara, A.H., Elsayed, G.F., Zimnik, A.J., Cunningham, J.P., and Churchland, M.M. (2018). Conservation of preparatory neural events in monkey motor cortex regardless of how movement is initiated. eLife *7*, e31826.

Li, N., Chen, T.W., Guo, Z.V., Gerfen, C.R., and Svoboda, K. (2015). A motor cortex circuit for motor planning and movement. Nature *519*, 51–56.

Low, A.Y.T., Thanawalla, A.R., Yip, A.K.K., Kim, J., Wong, K.L.L., Tantra, M., Augustine, G.J., and Chen, A.I. (2018). Precision of Discrete and Rhythmic Forelimb Movements Requires a Distinct Neuronal Subpopulation in the Interposed Anterior Nucleus. Cell Rep. *22*, 2322–2333.

Macpherson, J.M., Rasmusson, D.D., and Murphy, J.T. (1980). Activities of neurons in "motor" thalamus during control of limb movement in the primate. J. Neurophysiol. *44*, 11–28.

Martin, J.H. (1991). Autoradiographic estimation of the extent of reversible inactivation produced by microinjection of lidocaine and muscimol in the rat. Neurosci. Lett. *127*, 160–164.

Martin, J.H., Cooper, S.E., Hacking, A., and Ghez, C. (2000). Differential effects of deep cerebellar nuclei inactivation on reaching and adaptive control. J. Neurophysiol. *83*, 1886–1899.

Mason, C.R., Miller, L.E., Baker, J.F., and Houk, J.C. (1998). Organization of reaching and grasping movements in the primate cerebellar nuclei as revealed by focal muscimol inactivations. J. Neurophysiol. *79*, 537–554.

Mathis, A., Mamidanna, P., Cury, K.M., Abe, T., Murthy, V.N., Mathis, M.W., and Bethge, M. (2018). DeepLabCut: markerless pose estimation of user-defined body parts with deep learning. Nat. Neurosci. *21*, 1281–1289.

Meyer-Lohmann, J., Hore, J., and Brooks, V.B. (1977). Cerebellar participation in generation of prompt arm movements. J. Neurophysiol. *40*, 1038–1050.

Milak, M.S., Shimansky, Y., Bracha, V., and Bloedel, J.R. (1997). Effects of inactivating individual cerebellar nuclei on the performance and retention of an operantly conditioned forelimb movement. J. Neurophysiol. *78*, 939–959.

Mohammed, H., and Jain, N. (2016). Ipsilateral cortical inputs to the rostral and caudal motor areas in rats. J. Comp. Neurol. *524*, 3104–3123.

Morandell, K., and Huber, D. (2017). The role of forelimb motor cortex areas in goal directed action in mice. Sci. Rep. *7*, 15759.

Mushiake, H., and Strick, P.L. (1993). Preferential activity of dentate neurons during limb movements guided by vision. J. Neurophysiol. *70*, 2660–2664.

Nashef, A., Cohen, O., Israel, Z., Harel, R., and Prut, Y. (2018). Cerebellar Shaping of Motor Cortical Firing Is Correlated with Timing of Motor Actions. Cell Rep. *23*, 1275–1285.

Nashef, A., Cohen, O., Harel, R., Israel, Z., and Prut, Y. (2019). Reversible Block of Cerebellar Outflow Reveals Cortical Circuitry for Motor Coordination. Cell Rep. *27*, 2608–2619.e4.

Nenadic, Z., and Burdick, J.W. (2005). Spike detection using the continuous wavelet transform. IEEE Trans. Biomed. Eng. *52*, 74–87.

Oh, S.W., Harris, J.A., Ng, L., Winslow, B., Cain, N., Mihalas, S., Wang, Q., Lau, C., Kuan, L., Henry, A.M., et al. (2014). A mesoscale connectome of the mouse brain. Nature *508*, 207–214.

Pachitariu, M., Steinmetz, N., Kadir, S., and Carandini, M. (2016). Kilosort: real-time spike-sorting for extracellular electrophysiology with hundreds of channels. bioRxiv. https://doi.org/10.1101/061481.

Park, J., Phillips, J., Martin, K., Hantman, A., and Dudman, J. (2021). Descending neocortical output critical for skilled forelimb movements is distributed across projection cell classes. bioRxiv. https://doi.org/10.1101/772517.

Paxinos, G., and Franklin, K. (2008). The Mouse Brain in Stereotaxic Coordinates, Compact: The Coronal Plates and Diagrams, 3rd edition (Elsevier Science Publishing/Academic Press).

Petreanu, L., Mao, T., Sternson, S.M., and Svoboda, K. (2009). The subcellular organization of neocortical excitatory connections. Nature *457*, 1142–1145.

Pnevmatikakis, E.A., and Giovannucci, A. (2017). NoRMCorre: An online algorithm for piecewise rigid motion correction of calcium imaging data. J. Neurosci. Methods *291*, 83–94.

Reep, R.L., Goodwin, G.S., and Corwin, J.V. (1990). Topographic organization in the corticocortical connections of medial agranular cortex in rats. J. Comp. Neurol. *294*, 262–280.

Rouiller, E.M., Moret, V., and Liang, F. (1993). Comparison of the connectional properties of the two forelimb areas of the rat sensorimotor cortex: support for the presence of a premotor or supplementary motor cortical area. Somatosens. Mot. Res. *10*, 269–289.

Rovó, Z., Ulbert, I., and Acsády, L. (2012). Drivers of the primate thalamus. J. Neurosci. *32*, 17894–17908.

Sakai, S.T., Grofova, I., and Bruce, K. (1998). Nigrothalamic projections and nigrothalamocortical pathway to the medial agranular cortex in the rat: single- and double-labeling light and electron microscopic studies. J. Comp. Neurol. *391*, 506–525.

Sakayori, N., Kato, S., Sugawara, M., Setogawa, S., Fukushima, H., Ishikawa, R., Kida, S., and Kobayashi, K. (2019). Motor skills mediated through cerebellothalamic tracts projecting to the central lateral nucleus. Mol. Brain *12*, 13.

Sathyamurthy, A., Barik, A., Dobrott, C.I., Matson, K.J.E., Stoica, S., Pursley, R., Chesler, A.T., and Levine, A.J. (2020). Cerebellospinal Neurons Regulate Motor Performance and Motor Learning. Cell Rep. *31*, 107595.

Sauerbrei, B.A., Guo, J.Z., Cohen, J.D., Mischiati, M., Guo, W., Kabra, M., Verma, N., Mensh, B., Branson, K., and Hantman, A.W. (2020). Cortical pattern generation during dexterous movement is input-driven. Nature *577*, 386–391.

Sawyer, S.F., Young, S.J., Groves, P.M., and Tepper, J.M. (1994). Cerebellar-responsive neurons in the thalamic ventroanterior-ventrolateral complex of rats: in vivo electrophysiology. Neuroscience *63*, 711–724.

Schäfer, C.B., Gao, Z., De Zeeuw, C.I., and Hoebeek, F.E. (2020). Cerebello-Thalamic Spike Transfer via Temporal Coding and Cortical Adaptation. bioRxiv. https://doi.org/10.1101/2020.01.19.911610.

Schäfer, C.B., Gao, Z., De Zeeuw, C.I., and Hoebeek, F.E. (2021). Temporal dynamics of the cerebello-cortical convergence in ventro-lateral motor thalamus. J. Physiol. *599*, 2055–2073.

Schiemann, J., Puggioni, P., Dacre, J., Pelko, M., Domanski, A., van Rossum, M.C., and Duguid, I. (2015). Cellular mechanisms underlying behavioral state-dependent bidirectional modulation of motor cortex output. Cell Rep. *11*, 1319–1330.

Schindelin, J., Arganda-Carreras, I., Frise, E., Kaynig, V., Longair, M., Pietzsch, T., Preibisch, S., Rueden, C., Saalfeld, S., Schmid, B., et al. (2012). Fiji: an open-source platform for biological-image analysis. Nat. Methods *9*, 676–682.

Schmied, A., Benita, M., Conde, H., and Dormont, J.F. (1979). Activity of ventrolateral thalamic neurons in relation to a simple reaction time task in the cat. Exp. Brain Res. *36*, 285–300.

Shepherd, G.M. (2013). Corticostriatal connectivity and its role in disease. Nat. Rev. Neurosci. *14*, 278–291.

Spidalieri, G., Busby, L., and Lamarre, Y. (1983). Fast ballistic arm movements triggered by visual, auditory, and somesthetic stimuli in the monkey. II. Effects of unilateral dentate lesion on discharge of precentral cortical neurons and reaction time. J. Neurophysiol. *50*, 1359–1379.

Strick, P.L. (1976). Activity of ventrolateral thalamic neurons during arm movement. J. Neurophysiol. *39*, 1032–1044.

Tanaka, Y.H., Tanaka, Y.R., Kondo, M., Terada, S.I., Kawaguchi, Y., and Matsuzaki, M. (2018). Thalamocortical Axonal Activity in Motor Cortex Exhibits Layer-Specific Dynamics during Motor Learning. Neuron *100*, 244–258.e12.

Teune, T.M., van der Burg, J., van der Moer, J., Voogd, J., and Ruigrok, T.J. (2000). Topography of cerebellar nuclear projections to the brain stem in the rat. Prog. Brain Res. *124*, 141–172.

Thach, W.T. (1975). Timing of activity in cerebellar dentate nucleus and cerebral motor cortex during prompt volitional movement. Brain Res. *88*, 233–241.

Thach, W.T. (2014). Does the cerebellum initiate movement? Cerebellum *13*, 139–150.

Thach, W.T., and Bastian, A.J. (2004). Role of the cerebellum in the control and adaptation of gait in health and disease. Prog. Brain Res. *143*, 353–366.

Thanawalla, A.R., Chen, A.I., and Azim, E. (2020). The Cerebellar Nuclei and Dexterous Limb Movements. Neuroscience *450*, 168–183.

Thura, D., and Cisek, P. (2017). The Basal Ganglia Do Not Select Reach Targets but Control the Urgency of Commitment. Neuron *95*, 1160–1170.e5.

van Donkelaar, P., Stein, J.F., Passingham, R.E., and Miall, R.C. (1999). Neuronal activity in the primate motor thalamus during visually triggered and internally generated limb movements. J. Neurophysiol. *82*, 934–945.

van Donkelaar, P., Stein, J.F., Passingham, R.E., and Miall, R.C. (2000). Temporary inactivation in the primate motor thalamus during visually triggered and internally generated limb movements. J. Neurophysiol. *83*, 2780–2790.

Weiler, N., Wood, L., Yu, J., Solla, S.A., and Shepherd, G.M. (2008). Top-down laminar organization of the excitatory network in motor cortex. Nat. Neurosci. *11*, 360–366.

Wickersham, I.R., Finke, S., Conzelmann, K.K., and Callaway, E.M. (2007). Retrograde neuronal tracing with a deletion-mutant rabies virus. Nat. Methods *4*, 47–49.

Wickersham, I.R., Sullivan, H.A., and Seung, H.S. (2010). Production of glycoprotein-deleted rabies viruses for monosynaptic tracing and high-level gene expression in neurons. Nat. Protoc. *5*, 595–606.

Williams, S.F., and Herberg, L.J. (1987). Motivational vs. motor effects of striatal and pallidal gabergic projections to subthalamic and entopeduncular nuclei, ventromedial thalamus, and ventral globus pallidus. Pharmacol. Biochem. Behav. *26*, 49–55.

Witter, L., Canto, C.B., Hoogland, T.M., de Gruijl, J.R., and De Zeeuw, C.I. (2013). Strength and timing of motor responses mediated by rebound firing in the cerebellar nuclei after Purkinje cell activation. Front. Neural Circuits *7*, 133.

Xu, C., Krabbe, S., Gründemann, J., Botta, P., Fadok, J.P., Osakada, F., Saur, D., Grewe, B.F., Schnitzer, M.J., Callaway, E.M., and Lüthi, A. (2016). Distinct Hippocampal Pathways Mediate Dissociable Roles of Context in Memory Retrieval. Cell *167*, 961–972.e16.

Zong, W., Heldt, T., Moody, G.B., and Mark, R.G. (2003). An Open-Source Algorithm to Detect Onset of Arterial Blood Pressure Pulses. Comput. Cardiol. *30*, 4.

CellPress
OPEN ACCESS

STAR★METHODS

KEY RESOURCES TABLE

REAGENT or RESOURCE	SOURCE	IDENTIFIER
**Antibodies**		
Mouse monoclonal anti-Satb2	Thermo Fisher Scientific	Abcam Cat# ab51502; RRID:AB_882455
Rat monoclonal anti-Ctip2	Thermo Fisher Scientific	Abcam Cat# ab18465; RRID: AB_2064130
Anti-VGluT2 Antibody	Sigma-Aldrich	Cat# AB2251-I; RRID:AB_2665454
Cy5 AffiniPure donkey anti-guinea pig	Jackson ImmunoResearch Europe Ltd.	Cat# AB2251-I; RRID:AB_2340462
AlexaFluor-568 goat anti-mouse	Molecular Probes	Cat#: A- 21124; RRID: AB_2535766
AlexaFluor-647 goat anti-rat	Molecular Probes	Cat#: A-21247; RRID: AB_141778
**Bacterial and virus strains**		
AAV2/1-CAG-FLEX-mTagBFP2-2A-TVA	Kimberly Ritola at Janelia Farm-Molecular Biology Shared Resource; Huang et al., 2013	N/A
Pseudotyped SADΔG-mCherry(EnvA) rabies	Salk Institute Vector Core; Wickersham et al., 2007	N/A
AAV2/1-CAG-EGFP	Penn Vector Core	Addgene 28014
AAV1-Syn-GCaMP6s	Penn Vector Core; Chen et al., 2013	Addgene 100844
AAV1-CAG-ChR2-Venus	UNC Vector Core; Addgene; Petreanu et al., 2009	Addgene 20071
AAV2-CAG-mCherry	Homemade: McClure at Edinburgh	Addgene 108685
**Chemicals, peptides, and recombinant proteins**		
Muscimol hydrobromide	Sigma-Aldrich, Missouri, USA	Cat#: G019-5MG
Muscimol, BODIPY TMR-X Conjugate	Thermo Fisher Scientific	Cat#: M23400
Fast Blue	Polysciences	Cat#: 17740
Red Retrobeads™	Lumaflor	N/A
Green Retrobeads™	Lumaflor	N/A
Vybrant DiI Cell-Labeling Solution	Thermo Fisher Scientific	Cat#: V22885
NeuroTrace Blue Fluorescent Nissl Stain	Thermo Fisher Scientific	Cat#: N21479
Cholera Toxin Subunit B (Recombinant), Alexa Fluor 647	Thermo Fisher Scientific	Cat#: C34778
**Deposited data**		
Raw and analyzed data	This paper	N/A
**Experimental models: organisms/strains**		
Mouse: C57BL/6J	The Jackson Laboratory	RRID: IMSR_JAX:000664
Mouse: Rbp4-Cre: Tg(Rbp4-cre) KL100Gsat	The Jackson Laboratory	RRID:MMRRC_031125-UCD
**Software and algorithms**		
MATLAB	MathWorks (https://www.mathworks.com/)	RRID: SCR_001622
Python 3	Python (https://www.python.org/)	RRID: SCR_008394
AxonpCLAMP 10	Molecular Devices (https://www.moleculardevices.com/)	RRID:SCR_011323
Streampix 7.0	Norpix (https://www.norpix.com/products/streampix/streampix.php)	RRID:SCR_015773
NIS-Elements	Nikon (https://www.microscope.healthcare.nikon.com/products/software)	RRID:SCR_014329
Arduino IDE 1.6.5	Arduino (https://www.arduino.cc/en/software)	N/A

(*Continued on next page*)

Continued

REAGENT or RESOURCE	SOURCE	IDENTIFIER
SpikeGLX	Jun et al., 2017 (http://billkarsh.github.io/SpikeGLX/)	N/A
Mantis64	https://www.mantis64.com/	N/A
Kilosort3	https://github.com/MouseLand/Kilosort	N/A
Phy	Jun et al., 2017 (https://github.com/cortex-lab/phy)	N/A
FIJI	Schindelin et al., 2012 (https://github.com/fiji)	N/A
Deeplabcut	Adaptive Motor Control Lab (https://github.com/DeepLabCut/DeepLabCut)	N/A
NoRMCorre	Pnevmatikakis and Giovannucci, 2017 (https://github.com/flatironinstitute/NoRMCorre)	N/A
nmf_sklearn	Keemink et al., 2018 (https://github.com/rochefort-lab/fissa)	N/A
Onset detection algorithm	Zong et al., 2003	N/A
Data acquisition		
Data acquisition system	Molecular Devices	Digidata 1440A
Amplifier	Molecular Devices	Multiclamp 700B
Neuropixel probes	Janelia/IMEC	Phase 3B
Gradient-index (GRIN) lens	Grintech	Cat#: NEM-060-15-15-520-S-1.0p
Laser, Ti:Sapphire pulsed	Coherent	Chameleon Vision-S
Other		
Fiber optic cannula	ThorLabs	Cat#: CFMLC12L05
Optogenix tapered optic fiber cannula	Plexon	LambdaB (0,39, 200, 1.5, 225, 1.25)
Optic fiber patch cable		N/A
Laser, 473 nm	Civillaser	473nm 100 mW Blue DPSS Laser with Power Supply
Shutter	Uniblitz	Cat#: LS3S2T1
Arduino UNO	Arduino (https://www.arduino.cc/en/Guide/ArduinoUno/)	RRID:SCR_017284

RESOURCE AVAILABILITY

Lead contact

Further information and requests for resources and reagents should be directed to and will be fulfilled by the lead contact, Ian Duguid (ian.duguid@ed.ac.uk).

Materials availability

This study did not generate new unique reagents.

Data and code availability

Data analyzed and code generated in this study are available upon written request to corresponding author.

EXPERIMENTAL MODEL AND SUBJECT DETAILS

All experiments and procedures were approved by the University of Edinburgh local ethical review committee and performed under license from the UK Home Office in accordance with the Animal (Scientific Procedures) Act 1986. Male adult C57BL/6J wild-type (RRID: IMSR_JAX:000664) and Rbp4-Cre (RRID:MMRRC_031125-UCD) mice (5-14 weeks old, 20-30 g, one to six animals per cage) were maintained on a reversed 12:12 hour light:dark cycle (lights off at 7:00 am) and provided *ad libitum* access to food and water except during behavioral training and experimentation (see below).

METHOD DETAILS

General surgery

Mice undergoing surgery were induced under 4% and maintained under ~1.5% isoflurane anesthesia, with each animal receiving fluid replacement therapy (0.5 ml sterile Ringer's solution; to maintain fluid balance) and buprenorphine (0.5 mg/kg; for pain relief) at the beginning of each surgery. Additionally, buprenorphine (0.5 mg/kg) was administered in the form of an edible jelly cube ~24 hours after recovery from surgery. For surgeries involving removal of the periosteum, each animal received an injection of carprofen (5 mg/kg). A small lightweight headplate (0.75 g) was implanted on the surface of the skull using cyanoacrylate super glue and dental cement (Lang Dental, USA) and mice were left for 24-48 hours to recover. Craniotomies were performed in a stereotactic frame (Kopf, USA) using a hand-held dentist drill with 0.5 mm burr (craniotomy diameter: whole-cell patch-clamp recording ~⌀300 μm; viral / tracer / pharmacological compound injection ⌀500-1000 μm). Viral vectors and tracing compounds were delivered via pulled glass pipettes (5 μl, Drummond) using an automated injection system (Model Picospritzer iii, Intracell). At the end of each experiment, mice were anesthetized with euthatal (0.10–0.15 ml) and transcardially perfused with 30 mL of ice-cold 0.1 M phosphate-buffered saline (PBS) followed by 30 mL of 4% paraformaldehyde (PFA) in 0.1 M PBS solution. Brains were post-fixed in PFA overnight at 4°C then transferred to 10% sucrose solution for longer-term storage.

Behavioral training

Mice were handled extensively before being head restrained and habituated to the behavioral setup. To increase task engagement, mice were placed on a water control paradigm (1 ml/day) and weighed daily to ensure body weight remained above 85% of baseline. Mice were trained for one 30-minute session per day, during which they had to hold a moveable lever still during a random inter-trial-interval (ITI) of 4-6 s, before pushing the lever 4 mm during presentation of a 6 KHz auditory 'go cue' to receive a 5 μl water reward. The duration of the auditory cue (and thus response period) was reduced across training sessions in three stages: stage 1) 10 s, stage 2) 5 s, stage 3) 2 s, with mice advancing to the next stage after achieving > 80 rewards during a single session or > 50 rewards during two consecutive sessions. Mice were deemed "expert" after achieving > 80 rewards on two consecutive days of stage 3 training. Lever movements during the ITI would result in a lever reset and commencement of a subsequent ITI. After each 30-minute session, mice were removed from head restraint and given the remainder of their daily water allowance before being returned to their home cage.

In vivo pharmacology

To assess the behavioral effects of CFA, $MTh_{DN/IPN}$ or DN/IPN inactivation, a craniotomy was performed above the target area under general anesthesia. After 5/10 minutes of baseline task execution, the lever was locked and a small volume of the $GABA_A$ receptor agonist muscimol (dissolved in external solution containing 150 mM NaCl, 2.5 mM KCl, 10 mM HEPES, 1.5 mM $CaCl_2$ and 1 mM $MgCl_2$) or saline was injected into the target area (CFA: 200 nl of 2 mM muscimol at each of 5 sites centered on AP: 0.6, ML: 1.6, DV: −0.7 mm; $MTh_{DN/IPN}$: 200 nl of 1 mM muscimol, AP: −1.1, ML: 1.0, DV: −3.4 mm; ipsilateral DN: 100 nl of 1 mM muscimol, AP: −6.0, ML: −2.25, DV: −2.4 mm; ipsilateral IPN: 50nl of 1 mM muscimol, AP: −6.0, ML: −1.75, DV: −2.4 mm) at a rate of 5-20 nl/s. To confirm the anatomical location of drug injection, 1% w/v of retrobeads (Lumaflor Inc.) was included in the muscimol/saline solution. Mice were randomly assigned to drug or control groups, and experiments performed blinded. After each experiment, mice were transcardially perfused and coronal sections (60 μm) of CFA, $MTh_{DN/IPN}$ or DN/IPN were cut with a vibratome (Leica VT1000S), mounted with Vectashield mounting medium (H-1000, Vector Laboratories), imaged using a fluorescence microscope (Leica DMR, 5x objective) and manually referenced to the Paxinos and Franklin Mouse Brain Atlas (Paxinos and Franklin, 2008). Behavioral metrics were analyzed in 5-minute epochs using custom-written MATLAB (MathWorks) scripts, a two-sample t test was used to compare experimental cohorts during the first post injection epoch, and a one-way ANOVA was used to compare data between manipulation experiments. Behavioral video data for all pharmacology experiments was captured using a high speed camera (Genie HM640, Dalsa), and paw position accuracy was calculated as the proportion of trials in which mice were holding the moveable lever with their contralateral forepaw at the onset of the auditory cue.

GRIN lens imaging

To perform population calcium imaging in motor thalamus, 200 nl of AAV1-Syn-GCaMP6s was injected into contralateral $MTh_{DN/IPN}$ (AP: − 1.1, ML: 1.0, DV: − 3.4 mm) and mice were implanted with a lightweight headplate. 7-10 days after virus injection, a gradient-index (GRIN) lens (Grintech NEM-060-15-15-520-S-1.0p; 600 μm diameter, 4.83 mm length, 0.5 numerical aperture) was implanted as described previously (Xu et al., 2016). In brief, a sterile needle (1.1 mm OD) surrounding the GRIN lens was lowered to a depth of 3.2 mm and subsequently retracted leaving the lens at the desired depth. The lens was then secured in place with UV curing glue (Norland Products, USA) and dental cement. Fields of view were checked for clarity and GCaMP6s expression after implantation. After 4-8 weeks mice began water restriction and behavioral training. Two-photon calcium imaging was performed in expert mice during lever task execution with a 318 × 318 μm field of view (600 × 600 pixels) at 40 Hz frame rate, using a Ti:Sapphire pulsed laser (Chameleon Vision-S, Coherent, CA, USA; < 70 fs pulse width, 80 MHz repetition rate) tuned to 920 nm wavelength with a 40x objec-

tive lens. For confirmation of GRIN lens location and viral expression, mice were perfused, sagittal sections (100 μm) of $MTh_{DN/IPN}$ were cut with a vibratome, counterstained with Nissl blue, and imaged using a slide scanner (Axioscan, Zeiss). GRIN lens location was referenced to the Paxinos and Franklin Mouse Brain atlas.

Raw imaging videos were motion corrected using NoRMCorre (Pnevmatikakis and Giovannucci, 2017). In brief, NoRMCorre performs non-rigid motion correction by splitting each FOV into overlapping patches, estimating the xy translation for each patch, and upsampling the patches to create a smooth motion field, correcting for non-uniform motion artifacts caused by raster scanning or brain movement. Regions of interest (ROIs, polygonal areas) were drawn manually using Fiji (Schindelin et al., 2012). Signals were extracted and neuropil decontaminated using nmf_sklearn (Keemink et al., 2018). Normalized signal was calculated as $\Delta F/F_0$, where F_0 was calculated as the bottom 5th percentile of the 1Hz low pass filtered raw signal, and $\Delta F = (F-F_0)$. Normalized signals were then aligned to the behavioral data and analyzed using custom-written MATLAB scripts.

To detect activity changes of cells, a Friedman test was used to compare 250 ms time binned Ca^{2+} signals from 500 ms before movement to 1500 ms after movement with a significance threshold of $p < 0.01$. A Tukey-Kramer post hoc test was used to identify significantly different bins, and the direction of each response was defined based on the difference between baseline and the mean value of the Ca^{2+} signal in the earliest two significantly different bins. The median onset time of each cell was calculated by employing a previously published onset detection algorithm using a slope sum function (SSF; Zong et al., 2003) with the decision rule and window of the SSF adapted to the calcium imaging data (threshold 10% of peak, SSF window 375 ms, smoothed with a Savitzky Golay filter across 27 frames with order 2) and reported as the median of 10,000 bootstrapped samples to reduce the influence of noisy individual trials. Neurons whose bootstrapped samples had inter-quartile ranges exceeding 3 standard deviations of the median inter-quartile range were considered to have undetectable onset times and categorized as non-responsive. Prior to extracting $\Delta F/F_0$ onsets, we verified this algorithm with simulated data thereby accounting for any bias in the onset detection potentially introduced by filtering and/or the decision rule. To simulate the rising phase of the movement related calcium events in our data we used linear ramps with defined onset times and a rise time of 0.5 s mimicking GCaMP6s kinetics. We then calibrated the onset detection algorithm on the simulated data (100 simulated cells with 30 simulated trials per cell and artificially added noise in each trial matching the noise level in the imaging data) and updated it by a small FOV-specific correction factor.

Onset times were used to classify $MTh_{DN/IPN}$ neurons as preceding movement initiation (early) from those occurring after movement initiation (late). To investigate the relationship between $\Delta F/F_0$ trajectories and reaction time, reaction times were split into thirds (short [0 – 350 ms], medium [350 - 900 ms] and long [> 900 ms]) and only FOVs with a sufficient number of trials per reaction time category were included in further analysis. To compare the onset times across short, medium and long reaction time trials, the onset time of each neuron was calculated using only these subsets of trials. Movement-aligned time binned Ca^{2+} signals were presented smoothed with the loess method using a 40-frame sliding window and baseline corrected to the mean of the 500 ms pre-cue period. A kernel density estimate was calculated for each onset across all trials to calculate a mean. The area under the mean population kernel density estimate was calculated using numerical trapezoidal integration.

To investigate whether GRIN lens implantation surgery affected lever task execution, a two-sample Kolmogorov-Smirnov goodness-of-fit test was used to compare reaction time, push duration and task success (the ratio of the number of rewarded trials to total number of cued trials) of the GRIN lens-implanted cohort and a control group.

Whole-cell patch-clamp electrophysiology

Whole-cell patch-clamp recordings targeted to layer 5B, 550–1000 μm from the pial surface, were obtained from awake head restrained mice after performing a craniotomy and durotomy centered above CFA. Signals were acquired at 20 kHz using a Multiclamp 700B amplifier (Molecular Devices) and filtered at 10 kHz using PClamp 10 software in conjunction with a DigiData 1440 DAC interface (Molecular Devices). No bias current was injected during recordings and the membrane potential was not corrected for junction potential. Resting membrane potentials were recorded immediately after attaining the whole-cell configuration (break-in). Series resistances (Rs) ranged from 23.6 to 45.5 MΩ. Patch pipettes (5.5–7.5 MΩ) were filled with internal solution (285–295 mOsm) containing: 135 mM K-gluconate, 4 mM KCl, 10 mM HEPES, 10 mM sodium phosphocreatine, 2 mM MgATP, 2 mM Na_2ATP, 0.5 mM Na_2GTP, and 2 mg/ml biocytin (pH adjusted to 7.2 with KOH). External bath solution contained: 150 mM NaCl, 2.5 mM KCl, 10 mM HEPES, 1 mM $CaCl_2$, and 1 mM $MgCl_2$ (adjusted to pH 7.3 with NaOH). All electrophysiology recordings were analyzed using custom-written scripts in MATLAB. Individual action potentials (APs) were detected with a wavelet-based algorithm (Nenadic and Burdick, 2005) and AP threshold was defined as the membrane potential (V_m) at maximal d^2V/dt^2 up to 3 ms before AP peak and manually verified. For subthreshold V_m analysis APs were clipped by removing data points between −1 and +9 ms peri-AP threshold. Average AP firing frequencies were calculated by convolving spike times with a 50 ms Gaussian kernel. Significant changes in subthreshold V_m and AP firing frequency were defined by comparing bootstrapped 95% confidence intervals of mean movement-aligned V_m and AP frequency trajectories to zero (baseline epoch = 200 ms pre-cue; movement epoch = −100 to +100 ms peri-movement). Mean ΔV_m trajectories were calculated by subtracting the mean V_m during baseline (1 s epoch prior to cue) from the mean V_m during the peri-movement epoch (−250 to +250 ms when aligned to movement onset). All mean ΔV_m trajectories were decimated and median filtered with a 50 ms sliding window. Population mean ΔV_m trajectories were normalized to the largest absolute mean ΔV_m value in a 1.5 s peri-movement window. Peri-movement ΔV_m onsets were detected as the 10% rise-time of V_m trajectories when aligned to movement.

To inactivate motor thalamus during patch clamp recordings we performed a second craniotomy above $MTh_{DN/IPN}$ and a pipette containing 1 mM muscimol (dissolved in external solution) and 1% w/v red retrobeads was targeted to $MTh_{DN/IPN}$ (AP: −1.1, ML: 1.0, DV: −3.4 mm). Once the whole-cell recording configuration had been obtained, 5-10 minutes of baseline behavior and electrophysiological data were acquired before 200 nL of muscimol was injected at a rate of 5-10 nl/s. Mice were perfused, and data analyzed from animals in which retrobeads were found within $MTh_{DN/IPN}$. To compare subthreshold V_m dynamics during pre- and post- injection epochs, cue-aligned periods of V_m were baseline subtracted and the area under the $|\Delta V_m|$ trajectory from cue onset to median reward delivery was calculated via trapezoidal numerical integration with a 50 ms sample rate. To compare firing rate dynamics, the proportional difference between peri-median reaction time versus baseline Gaussian kernel smoothed firing rate was calculated in both epochs using bin sizes described previously.

Immunohistochemistry

To morphologically identify neurons after whole-cell patch-clamp recording, mice were transcardially perfused and coronal sections (60 μm) of CFA were cut with a vibratome. To recover neurons, sections were incubated in streptavidin AlexaFluor-488 (1:1000, Molecular Probes) in 0.1 M PBS containing 0.5%Triton X-100, mounted, imaged using a confocal microscope (Zeiss LSM800, 20x objective) and referenced to the Paxinos and Franklin Mouse Brain Atlas. To identify projection targets of individually recorded neurons (Schiemann et al., 2015), sections were further processed by heat-mediated antigen retrieval in 10 mM sodium citrate buffer (pH 6.0) for 3 hours at 80°C. Sections were incubated in blocking solution (0.01 M PBS, 10% normal goat serum (NGS), 0.5% Triton X-100) at 22°C for 2 hours and incubated overnight at 22°C in a primary antibody mixture containing mouse monoclonal anti-Satb2 (1:200, Cat. No. ab51502, Abcam) and rat monoclonal anti-Ctip2 (1:1000, Cat. No. ab18465, Abcam) dissolved in carrier solution (0.01 M PBS, 1% NGS, 0.5% Triton X-100). Slices were then incubated overnight at 22°C in a secondary antibody mixture containing AlexaFluor-568 goat anti-mouse (1:750, Molecular Probes) and AlexaFluor-647 goat anti-rat (1:750, Molecular Probes) dissolved in carrier solution (0.01 M PBS, 1% NGS, 0.5% Triton X-100), mounted and imaged using a confocal microscope (Zeiss LSM800, 20x objective).

To assess the proportion of CFA-projecting $MTh_{DN/IPN}$ neurons that receive glutamatergic synaptic input from dentate/interpositus nuclei, selected motor thalamic coronal sections (60 μm) were rinsed thee times with 0.1 M PBS for 10 minutes, incubated for 2 hours at room temperature in blocking solution (containing 10% normal horse serum (NHS) and 0.5% triton diluted in 0.1 M PBS), rinsed briefly in 0.1 M PBS and incubated overnight with a primary antibody for vesicular glutamate transporter type 2 (VGluT2) (anti-guinea pig, Millipore Bioscience; diluted 1:2000 in 0.1 M PBS containing 1% NHS and 0.5% Triton-X). Slices were then rinsed four times in 0.1 M PBS for 10 minutes before being incubated for at least 2 hours with secondary antibody anti-Guinea Pig Cy5 (diluted 1:200 in in 0.1 M PBS containing 1% NHS and 0.5% Triton-X). Sections were rinsed four times in 0.1 M PBS for 10 minutes, mounted with Vectashield mounting medium and imaged using a confocal microscope (Leica LS8; 63x objective). Fast Blue labeled cells with overlapping Venus-labeled + VGluT2 +ve axons (with 1 μm) were manually counted.

Motor thalamic activation

For optogenetic activation of $MTh_{DN/IPN}$ neurons or their axon terminals in CFA, 250 nl of AAV1-CAG-ChR2-Venus ($2.3x10^{12}$ GC/ml, Addgene 20071; control virus: AAV2-CAG-mCherry ($5.2x10^{11}$ GC/ml)) was injected into contralateral $MTh_{DN/IPN}$ (AP: −1.1, ML: 1.0, DV: −3.4 mm). For direct $MTh_{DN/IPN}$ stimulation, an optic fiber (200 μm diameter, 0.39 NA; Thorlabs) was implanted ~300 μm dorsal to the viral injection site (sealed with RelyX Unicem2 Automix cement, 3M) and trains of pulsed 473 nm light (5-8 mW, 16.6 Hz pulse frequency, 33.3% duty cycle) were delivered using a solid-state laser (DPSS, Civillaser, China) and shutter (LS3S2T1, Uniblitz) controlled by an Arduino control system, coupled to the implanted optic fiber by means of an optic patch cable (Thorlabs, FT200UMT). For direct simulation of $MTh_{DN/IPN}$ axon terminals, a tapered optic fiber (Optogenix, Italy) was implanted to a depth of 1 mm at the center of CFA (AP: 0.6, ML: 1.6, DV: −1.0 mm) and 12 mW, 473 nm light was delivered as above. Prior to optogenetic stimulation experiments, mice were trained to expert level performance and habituated to light emanating from an uncoupled optic patch cable and the sound of shutter activation. During habituation and experimental sessions, mice were exposed to 3 different trial types: (1) cue and shutter; (2) laser and shutter; and (3) shutter only. Trials were presented with the following pattern: 1, 1, 3, 1, 1, 2,... repeating for 30 minutes. $MTh_{DN/IPN}$ axon terminal stimulation in CFA was performed on the following day, while whole-cell patch-clamp recordings from CFA were performed in combination with direct $MTh_{DN/IPN}$ stimulation in a separate cohort of mice. To investigate the effects of behavioral context, mice which had previously undergone $MTh_{DN/IPN}$ stimulation were head restrained above a 3D printed baseplate (Wanhao i3 Duplicator) without support/movable levers or reward spout (ABC), or within the same lever pressing apparatus with the reward and moveable levers replaced by a 3D printed platform (ABC2), and habituated for 2 sessions, interleaved with normal training to ensure that the cued motor behavior was not extinguished. To compare effects of $MTh_{DN/IPN}$ stimulation in the learned and altered behavioral contexts, mice first underwent a 15-minute optogenetic stimulation protocol in the learned context, before being exposed to an identical 15-minute optogenetic stimulation protocol in one of the two altered behavioral contexts (ABC or ABC2). To investigate the effects of $MTh_{DN/IPN}$ stimulation during freely moving behavior, mice were placed in an open field arena (dimensions 30 × 20 cm) with a camera phone (Samsung Galaxy S5) recording the full arena from beneath. A patch cable was coupled to the $MTh_{DN/IPN}$ optic fiber, and mice underwent a 15-minute optogenetic stimulation protocol. Peri-trial movements

were classified from the videos as push-like (a single movement of the left forepaw in a defined forward direction), discrete (a single movement of the left forepaw in any other direction), locomotion (> 2 consecutive steps/strides made by the left forelimb) and grooming, by two researchers and cross validated.

For histological confirmation of the injection site and optic fiber placement, mice were transcardially perfused, decapitated and the whole head (including headplate and optic fiber) was post-fixed in 4% PFA for 2 days to improve preservation of the optic fiber tract. Coronal sections (60 μm) of CFA and $MTh_{DN/IPN}$ were cut with a vibratome, mounted with Vectashield, and imaged using a slidescanner (Axioscan, Zeiss). The center of the optic fiber (COF) was defined as the most ventral extent of the optic fiber tract across all slices from each brain as measured from the pial surface. Where tracts of equal depth were present, the coronal section containing the largest diameter tract tip was identified as the COF. The expression of ChR2-Venus in $MTh_{DN/IPN}$ was coarsely defined by first referencing three coronal slices (120 μm spacing) centered on the COF to the Paxinos and Franklin Mouse Brain Atlas before manually evaluating the proportion of each of the principle motor thalamic nuclei (AM, anteromedial; VL, ventrolateral; VPM, ventral posteromedial nucleus; VPL, ventral posterolateral; VM, ventromedial) containing fluorescence, and categorizing three levels based on expression covering 0%–5%, 5%–50% and 50%–100% of each nucleus. Data were not included from mice in which the COF was misaligned to virus expression. To investigate whether photostimulation of $MTh_{DN/IPN}$ evokes tongue movements, a ROI was drawn in front of the mouth and tongue movements were detected using a motion index threshold (see below).

The proportion of full and partial push-like movements in cue- and laser- trials were calculated by correcting for the behavioral "error" rate, i.e., subtracting the proportion of pushes observed in shutter only trials (trial type 3) to obtain a lower bound. ΔV_m trajectories for both cue-evoked and photoactivation-evoked movement trials were calculated as described previously, and trial-by-trial ΔV_m changes were based on comparing the 200ms pre-cue or pre-photoactivation epoch with the 200 ms peri-movement epoch within each trial.

Cerebellar-motor thalamic pathway tracing and activation

For tracing and activation of the dentate/interpositus-motor thalamus pathway, AAV1-CAG-ChR2-Venus (2.3×10^{12} GC/ml, Addgene 20071) was injected unilaterally into ipsilateral dentate (AP: −6.0, ML: −2.25, DV: −2.6 & −2.2 mm) and interpositus (AP: −6.0, ML: −1.75, DV: −2.4 mm) cerebellar nuclei, with 75 nl injected at each depth within each nucleus. For activation of dentate/interpositus axons in motor thalamus, an optic fiber (200 μm diameter, 0.39 NA; Thorlabs) was implanted into contralateral $MTh_{DN/IPN}$ (AP: −1.1, ML: 1.0, DV: −3.2 mm) and trains of pulsed 473 nm light (8 mW, 16.6 Hz pulse frequency, 33.3% duty cycle) were delivered as previously described. Mice were trained to expert level performance, habituated to light emanating from an uncoupled optic fiber and the sound of shutter activation and exposed to the same alternating trial structure as for $MTh_{DN/IPN}$ activation experiments described previously. For histological confirmation of the injection site, mice were transcardially perfused and coronal sections (60 μm) of $MTh_{DN/IPN}$ and DN/IPN were cut with a vibratome, mounted, and imaged using a slidescanner (Axioscan, Zeiss). Optic fiber location within $MTh_{DN/IPN}$ was ascertained as described previously. The expression of ChR2-Venus in DN/IPN was coarsely defined by first referencing the three coronal slices centered on the DN and IPN to the Paxinos and Franklin Mouse Brain Atlas before manually evaluating the proportion of each of the cerebellar (DN, dentate; IPN, interpositus; FN, fastigial) and vestibular nuclei containing fluorescence, and categorizing three levels based on expression covering 0%–5%, 5%–50% and 50%–100% of each nucleus. Data were not included from mice that had insufficient ChR2-Venus expression in DN and IPN, or in which the COF was not aligned to $MTh_{DN/IPN}$.

To map DN/IPN projections to CFA-projecting neurons in $MTh_{DN/IPN}$, some mice underwent surgery to perform an additional craniotomy above contralateral CFA (AP: 0.6, ML: 1.6 mm), where Fast Blue retrograde tracer (Polysciences; 0.2% Fast Blue in 1 M PB with 0.2% DMSO) was injected at four points equidistant from the center of the CFA craniotomy, with 100 nl injected at two depths, −800 μm and −400 μm below the pial surface. After recovery, mice were returned to the home cage for a further 5 days, before being transcardially perfused. Coronal sections (60 μm) of CFA, $MTh_{DN/IPN}$ and DN/IPN were cut with a vibratome, mounted using Vectashield, and imaged using a confocal microscope (Leica SP8; 20x objective). Raw data images of coronal sections of the motor thalamus were manually referenced to the Paxinos and Franklin Mouse Brain Atlas, aligned and cropped to the same exact motor thalamic subregion. These cropped images were combined into a stack using Fiji and an average intensity projection of each channel (Venus & Fast Blue) was calculated. The resultant average image for each channel were considered as a matrix of gray-scale pixel values in MATLAB, and to calculate a matrix of proportional overlap of the two channels, the two matrices were square-rooted and then multiplied together. A 2-D Gaussian smoothing kernel with SD = 5 pixels was then used to smooth the resultant image which was then remapped with the Jet colormap. For the density plots of individual channels, the average projection matrices were similarly smoothed and remapped.

To assess the density of CFA-projecting neurons in ventrolateral motor thalamus, 200 nl of CTB-Alexa647 (ThermoFisher) was injected into contralateral CFA (AP: 0.6, ML: 1.6, DV: −0.7 mm). After recovery, mice were returned to the home cage for ~7 days before being perfused. Coronal sections (100 μm) of $MTh_{DN/IPN}$ were collected, counterstained with NeuroTrace Nissl blue (ThermoFisher), mounted using Vectashield mounting medium and imaged with a confocal microscope (Leica LSM800). Cells were counted in a representative 300 × 300 μm region and counts were independently verified.

To quantify vestibular nuclei projections to motor thalamus, we performed a craniotomy above contralateral $MTh_{DN/IPN}$ (AP: −1.1, ML: 1.0 mm) and injected 100 nl of Fast Blue at a depth of 3.4 mm below the pial surface. After recovery, mice were returned to the home cage for 5 days, before being perfused, and brains processed and imaged as described above.

CellPress
OPEN ACCESS

Monosynaptic retrograde rabies tracing

For monosynaptic retrograde rabies tracing, conditional expression of TVA receptor was achieved by injecting 60 nl of AAV2/1-CAG-FLEX-mTagBFP2-2A-TVA (9.0x10^{12} GC/ml) into contralateral $M1_{FL}$ (AP: 0.6, ML: 1.6, DV: −0.7 mm) of three Rbp4-Cre mice. Pseudotyped SADΔG-mCherry(EnvA) rabies virus (produced as previously described (Wickersham et al., 2007, 2010) was injected into CFA three weeks after the initial injections. Mice were perfused seven days post-rabies virus injection. Coronal sections (60 μm) were cut, mounted and imaged using a Nanozoomer Slide Scanner (Hamamatsu, 20x objective). Raw data images were manually referenced to the Paxinos and Franklin Mouse Brain Atlas and the distribution of fluorescence was manually outlined and independently verified.

Extracellular recording and spike sorting

To compare neural activity during cue-evoked and photoactivated movements, extracellular unit recordings in CFA were performed using acutely implanted silicone probes (Neuropixels Phase 3B probes, IMEC). Data were acquired from the 384 channels closest to the probe tip. Data were acquired with SpikeGLX software at 30 KHz with an amplifier gain of 500 for each channel and high-pass filtered with a cutoff frequency of 300 Hz. Spike sorting was performed using Kilosort3 to automatically cluster units from raw data (Pachitariu et al., 2016). The resulting spike clusters were manually curated using Phy (https://github.com/cortex-lab/phy), and any unit with sufficient refractory period violations, inconsistent waveform amplitude across the duration of the recording, or clipped amplitude distribution was excluded from analyses. Probe location was confirmed via DiI (Thermofisher) reconstruction of the recording track, and units from 500-1200 μm below the pial surface were included in our analyses. To detect changes in activity, firing rates were calculated by convolving spike times with a 200 ms Gaussian kernel and mean changes in firing rate were calculated by subtracting the firing rate during a baseline period (200 ms period before cue or laser presentation) from a response period (−100 to +100 ms peri-movement onset). Significant changes were identified by comparing bootstrapped 95% confidence intervals of these mean changes in firing rates to 0. Firing rate trajectories are presented as spike times convolved with a 50 ms Gaussian kernel. Spike time rasters are presented showing a random sample of cue trials matching the number of photostimulation trials. Spike widths were calculated as the duration from trough to peak of the spike waveform.

Quantifying muscimol diffusion

To measure muscimol diffusion, a small volume of muscimol-BODIPY TMR-X Conjugate (ThermoFisher Scientific; dissolved in 0.1 PBS w/1% dimethyl sulfoxide) was injected into $MTh_{DN/IPN}$ (200 nl of 1 mM), CFA (100 nl of 2 mM at −700 μm and −400 μm below the pial surface at 5 sites centered on CFA) or DN/IPN (100 nl and 50 nl of 1mM, respectively). To mark the center of injection, pipettes were backfilled with a small volume (~20 nl) of green (505 nm) retrobeads (Lumafluor Inc.) prior to filling with muscimol-BODIPY. Following injection, animals were transcardially perfused and brains snap-frozen on dry ice within 10 minutes of completion of muscimol injection. Brains were stored on dry ice, coronal sections (60 μm) collected with a cryostat (Leica) at −20°C and imaged with a light microscope (Leica DMR, 5x objective). We assumed maximum fluorescence ≈ maximum injected concentration and that grayscale pixel intensity was proportional to muscimol-BODIPY concentration. Therefore, pixel values were thresholded at the equivalent pixel value of an EC_{20} concentration of muscmiol and fluorescence outlines were drawn to generate a 'spread profile'. Green retrobeads were used to mark the center of each injection, and images were aligned to the injection center of gravity. From the aligned profiles, a modal spread profile (i.e., pixels with positive grayscale values across all mice) was generated and aligned to the Paxinos and Franklin Mouse Brain Atlas.

To assess the functional time course of muscimol inactivation, a silicone probe was vertically implanted into CFA (AP: 0.6, ML: 1.2-1.6, DV: −2.0 mm) of naive head restrained mice at rest. 15 minutes of baseline activity was recorded, after which 200 nl of 1 mM muscimol (containg 1% w/v red retrobeads) was injected at a point 500-700 μm horizontal from the shank of the probe (AP: 0.6, ML: 1.8-2.4, DV: −1.0 mm). The recording was continued for a further 30 minutes before perfusing and collecting tissue as described previously. Probe location was confirmed by DiI labeling, neural activity was spike sorted and analyzed as previously described, individual units were localized and the change in spike rate over time was correlated with the distance of each individual unit from the center of muscimol injection.

Forelimb kinematic tracking

Behavior was recorded using a high-speed camera (Pharmacological experiments: Genie HM640, Dalsa; optogenetic experiments: Mako U U-029, Allied Vision) and acquired with Streampix 7 (Norpix), synced using a TTL output from the DigiData 1440 DAC interface. Forepaw and wrist positions during pharmacological inactivation experiments were calculated by tracking forepaw markers using a custom written tracking script in Blender (2.79b, Blender Foundation). Directional tracking of forelimb movement in the learned/altered behavioral contexts was performed using Deep Lab Cut, a markerless video tracking toolbox (Mathis et al., 2018). Initial paw vector trajectories were plotted for the 50 ms post movement onset epoch in the learned behavioral context (LBC), and for the altered behavioral contexts (ABC & ABC2) we plotted trajectories in the epoch 50 ms after the LBC median reaction time. Push-like movements were defined as trials with an initial paw trajectory vector between 170° and 210°, and manually verified. To measure gross forelimb movement, we defined a region-of-interest (ROI) covering the contralateral (left) forelimb and calculated a motion index (MI) for each successive frame f as $MI_f = \sum_{i=1}^{N} (c_{f+1,i} - c_{f,i})^2$, where $c_{f,i}$ is the grayscale level of the pixel of the ROI, pixels per ROI (Schiemann et al., 2015). Movement trials were defined by calculating the MI > θ within 500 ms of cue/photostimulation

onset, with the threshold θ defined as three standard deviations above the mean MI for detecting forelimb movements, and 10 standard deviations above the mean MI for detecting licking.

QUANTIFICATION AND STATISTICAL ANALYSIS

Data analysis was performed using custom-written scripts in MATLAB or Python3 and code will be made available on request. All statistical details of experiments can be found in the figure legends, including description of the specific test used and sample sizes. Data are reported as mean ± 95% bootstrapped confidence interval, 10,000 bootstrap samples, unless otherwise indicated. Where multiple measurements were made from a single animal, suitable weights (proportional to the contribution from each animal) were used to evaluate summary population statistics and to obtain unbiased bootstrap samples. Statistical comparisons using the significance tests stated in the main text were made in MATLAB, and statistical significance was considered when $p < 0.05$ unless otherwise stated. Data were tested for normality with the Shapiro–Wilk test, and parametric/non-parametric tests were used as appropriate and as detailed in the text. Data inclusion/exclusion criteria have been listed throughout the method details section where relevant.

Neuron, Volume *109*

Supplemental information

A cerebellar-thalamocortical pathway drives behavioral context-dependent movement initiation

Joshua Dacre, Matt Colligan, Thomas Clarke, Julian J. Ammer, Julia Schiemann, Victor Chamosa-Pino, Federico Claudi, J. Alex Harston, Constantinos Eleftheriou, Janelle M.P. Pakan, Cheng-Chiu Huang, Adam W. Hantman, Nathalie L. Rochefort, and Ian Duguid

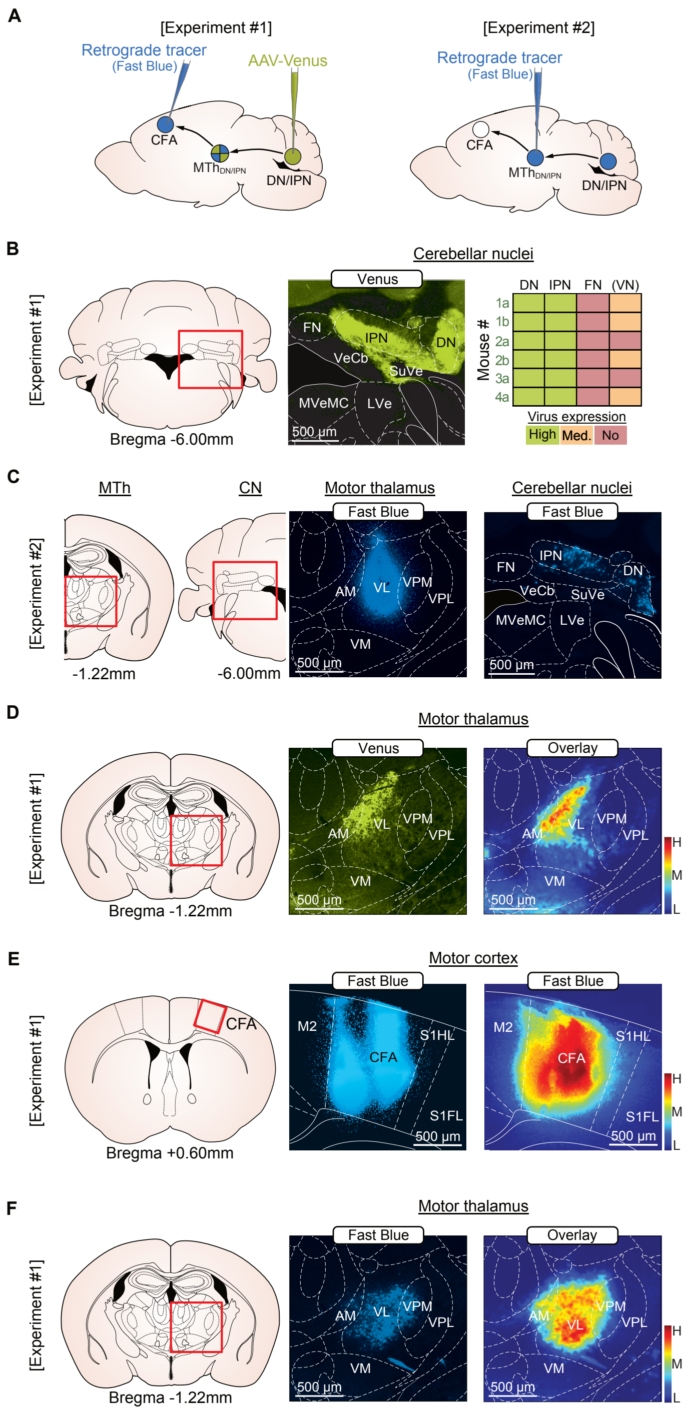

Figure S1. Mapping the DN/IPN thalamocortical pathway, related to Figure 1.
(A) *Experiment #1*, retrograde tracing of CFA-projecting neurons (Fast Blue) and anterograde tracing of DN/IPN axons (AAV-Venus) in motor thalamus. *Experiment #2*, retrograde tracing of VAL-projecting neurons in cerebellar nuclei (Fast Blue). (B) *Left & middle*, Virus labelling of cerebellar nuclei. *Right*, quantification of Venus expression in cerebellar and vestibular nuclei (red/no = <5 %, orange/medium = 5-50 % & green/high = 50-100 % expression within each nuclei) (bilateral injection, n = 6 slices from N = 4 mice). DN, dentate nucleus; IPN, interpositus nucleus; FN, fastigial nucleus, VN, vestibular nuclei including: VeCb, vestibulocerebellar nuclei; SuVe, superior vestibular nucleus; MVeMC, medial vestibular nucleus magnocellar part; LVe, lateral vestibular nucleus. (C) *Left & middle*, Fast Blue injection site in motor thalamus centered on VL. *Right*, retrograde labelling of cerebellar and vestibular nuclei neurons. AM, anteromedial; VL, ventrolateral; VPM, ventral posteromedial; VPL, ventral posterolateral; VM, ventromedial thalamic nuclei. (D) *Left & Middle*, Anterograde labelling of DN/IPN axons in motor thalamus. *Right*, average density of DN/IPN axons across motor thalamic nuclei (N = 6 slices from 4 mice). Scale bar, H - high, M - medium, L - low-level expression. (E) *Left & centre*, Fast Blue injection site in CFA. Right, average density of Fast Blue in CFA (N = 6 slices from 4 mice). Scale bar, H - high, M - medium, L - low-level fluorescence. (F) *Left & Middle*, Retrograde labelling of CFA-projecting thalamic neurons. *Right*, average density of CFA-projecting neurons across thalamic nuclei (N = 6 slices from 4 mice). Scale bar, H - high, M - medium, L - low-level expression.

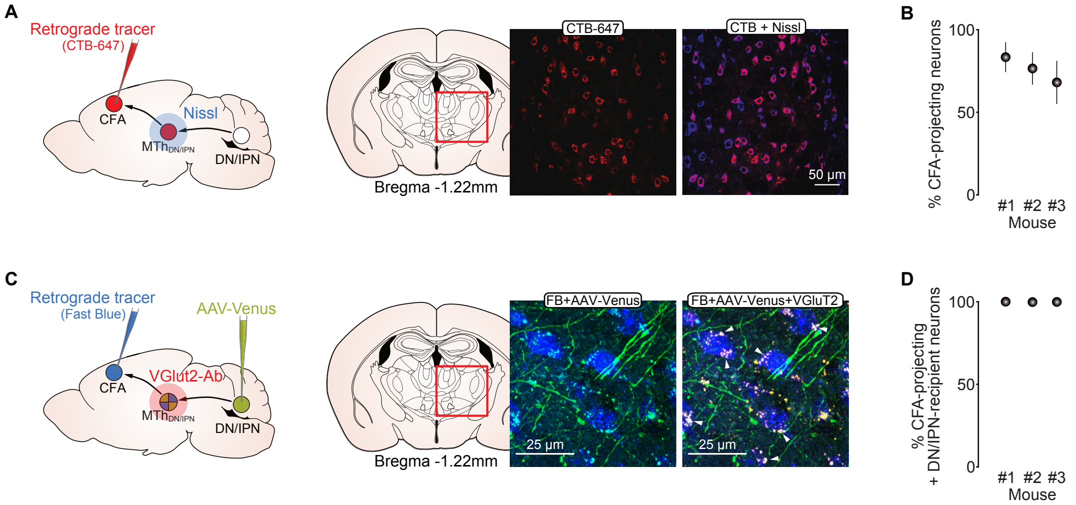

Figure S2. Quantification of CFA-projecting & DN/IPN-recipient neurons in ventral motor thalamus, related to Figure 1.
(A) *Left*, Nissl stain and retrograde labelling of CFA-projecting neurons in ventrolateral thalamus (CTB-647). *Right*, retrograde labelling of CFA-projecting neurons in ventrolateral thalamus (CTB-647) with all neurons labelled with Nissl. (B) Proportion of CFA-projecting motor thalamic neurons in VL thalamus (N = 3 mice, 4-6 slices per mouse, mean ± bootstrapped 95% CI). Filled circles represent population means ± 95% CI. (C) *Left & right,* retrograde tracing of CFA-projecting neurons (Fast Blue) and anterograde tracing of DN/IPN axon terminals (AAV-Venus + VGluT2) in ventrolateral motor thalamus. (D) Proportion of CFA-projecting motor thalamic neurons in VL thalamus that receive glutamatergic synaptic input from dentate/interpositus nuclei (N = 3 mice, 2-4 slices per mouse, mean ± bootstrapped 95% CI). Filled circles represent population means ± 95% CI.

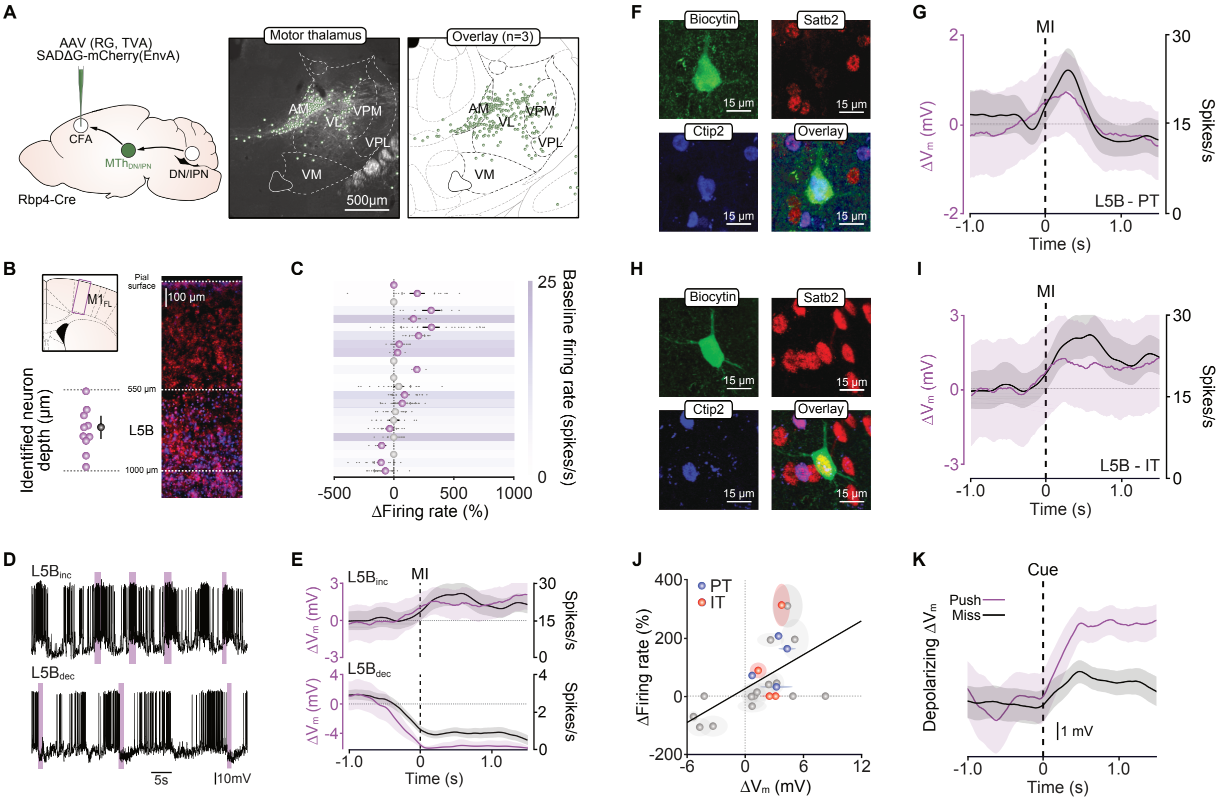

Figure S3. Membrane potential dynamics and projection class identity of CFA L5B neurons, related to Figure 3.
(A) *Left*, Monosynaptic rabies tracing strategy: injection of AAV2/1-CAG-FLEX-mTagBFP2-2A-TVA & SADΔG-mCherry(EnvA) into caudal forelimb motor area (CFA) of an Rbp4-Cre mouse. *Centre & Right*, CFA-projecting neurons in ventral motor thalamus. AM, anteromedial; VL, ventrolateral; VPM, ventral posteromedial; VPL, ventral posterolateral; VM, ventromedial thalamic nuclei. (B) *Top left*: Schematic coronal brain slice showing location of CFA. Purple rectangle depicts the expanded view shown on the right. *Right:* Distribution of PT-type (blue, Ctip2 staining) and IT-type (red, Satb2 staining) projection neurons in layer 5B of CFA. *Bottom left:* depth of recovered layer 5B neurons as measured perpendicularly from the pial surface (n = 11/23 neurons identified, black symbol represents mean ± 95% CI). (C) Average firing rate change ± 95% CI as a function of baseline firing rate. Gray dots represent individual trials, purple symbols represent significant changes in firing rate, gray symbols represent non-significant changes, defined by comparing 95% bootstrapped confidence intervals. (D) Voltage traces from a (*top*) depolarizing and (*bottom*) hyperpolarizing layer 5B neuron across multiple trials. Filled purple bars depict push trials. (E) Average subthreshold ΔV_m (purple) and firing rate (FR, black) trajectories for the layer 5B neurons shown in *(B)* aligned to movement initiation (MI). Thick lines represent the mean ± 95% CI. (F) Biocytin staining (green) and post-hoc immunohistochemical staining for Satb2 (red) and Ctip2 (blue) confirmed the PT-type projection class identity of an individually recorded layer 5B pyramidal neuron. (G) Mean subthreshold V_m and firing rate of the layer 5B PT-type projection neuron depicted in *(F)*. (H) Biocytin staining (green) and post-hoc immunohistochemical staining for Satb2 (red) and Ctip2 (blue) confirmed the IT-type projection class identity of an individually recorded L5B pyramidal neuron. (I) Mean subthreshold V_m and firing rate of the L5B IT-type projection neuron depicted in *(H)*. (J) Correlation between movement-related subthreshold ΔV_m and firing rate changes. Blue/red symbols represent means ± 95% CI from individual PT-/IT-type neurons respectively, black line is a linear fit to the data. Gray symbols represent means ± 95% CI from neurons where projection class identity was not determined (N = 23 neurons from 23 mice). (K) Example cue aligned push-trial mean ΔVm (purple) vs miss-trial mean ΔVm (black) trajectories from a representative layer 5B neuron in CFA.

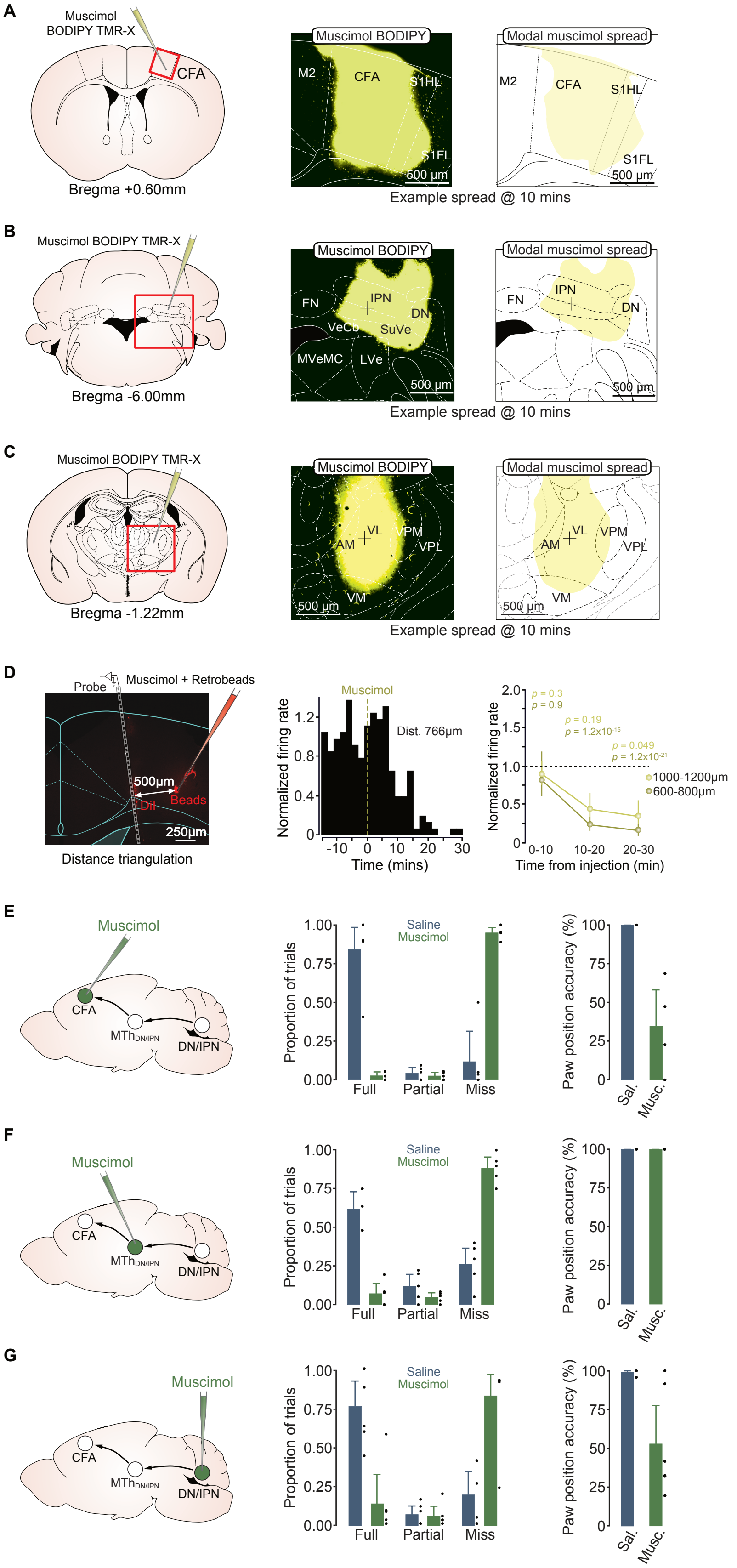

Figure S4. Diffusional spread and behavioral effects of muscimol in CFA, cerebellar nuclei and ventral thalamus, related to Figure 4.
(A) *Left*, injection of muscimol BODIPY TMR-X into CFA. *Middle*, example image of fluorescent muscimol spread in CFA at 10 mins post injection. *Right*, Modal spread of fluorescent muscimol in CFA (i.e., area in which fluorescence is present across all mice) (N = 3 mice). M2, secondary motor cortex; CFA, caudal forelimb area; S1HL, primary hindlimb somatosensory cortex. (B) *Left*, injection of muscimol BODIPY TMR-X targeted to dentate and interpositus cerebellar nuclei. *Middle*, example image of fluorescent muscimol spread across cerebellar nuclei at 10 mins post injection. *Right*, Example of diffusional spread outline used to calculate the modal spread shown in in Figure 4B. DN, dentate nucleus; IPN, interpositus nucleus; FN, fastigial nucleus, VN, vestibular nuclei including: VeCb, vestibulocerebellar nuclei; SuVe, superior vestibular nucleus; MVeMC, medial vestibular nucleus magnocellar part; LVe, lateral vestibular nucleus. The cross represents the median point of injection located using fluorescent retrobeads (N = 4 mice). (C) *Left*, injection of muscimol BODIPY TMR-X targeted to $MTh_{DN/IPN}$. *Middle*, example image of fluorescent muscimol spread in ventral thalamus at 10 mins post injection. *Right*, Example of diffusional spread outline used to calculate the modal spread shown in in Figure 4C. AM, anteromedial; VL, ventrolateral; VPM, ventral posteromedial; VPL, ventral posterolateral; VM, ventromedial thalamic nuclei. The cross represents the median point of injection located using fluorescent retrobeads (N = 4 mice). (D) *Left*, example silicon probe tract through cortex visualized using DiI and muscimol injection site visualized using red fluorescent retrobeads. The distance between the centre of the bead injection and the electrode position of each recorded unit was used to group neurons (600-800 µm or 1000-1200µm). *Middle*, normalized firing rate as a function of time from muscimol injection in an example CFA neuron. *Right*, normalized suppression of firing rate in CFA neurons as a function of time and distance. Group: 600-800 µm, n = 43 neurons; 1000-1200 µm, n = 25 neurons, one-way t-test for significant differences from baseline at each time point. (E) *Left*, Muscimol injection into CFA. *Middle*, Proportion of full / partial lever pushes and miss trials 10 minutes after saline (blue) or muscimol (green) injection into CFA. Black dots represent data from individual mice, bars represent population means ± 95% CI. *Right*, Paw position accuracy at the point of cue presentation and 10 mins after saline (Sal., blue) or muscimol (Musc., green) injection into CFA (N = 5 and 5 mice). (F) Same as *(E)* but with injections targeted to $MTh_{DN/IPN}$. (N = 5 and 5). (G) Same as *(E)* but with injections targeted to DN/IPN (N = 5 and 5 mice).

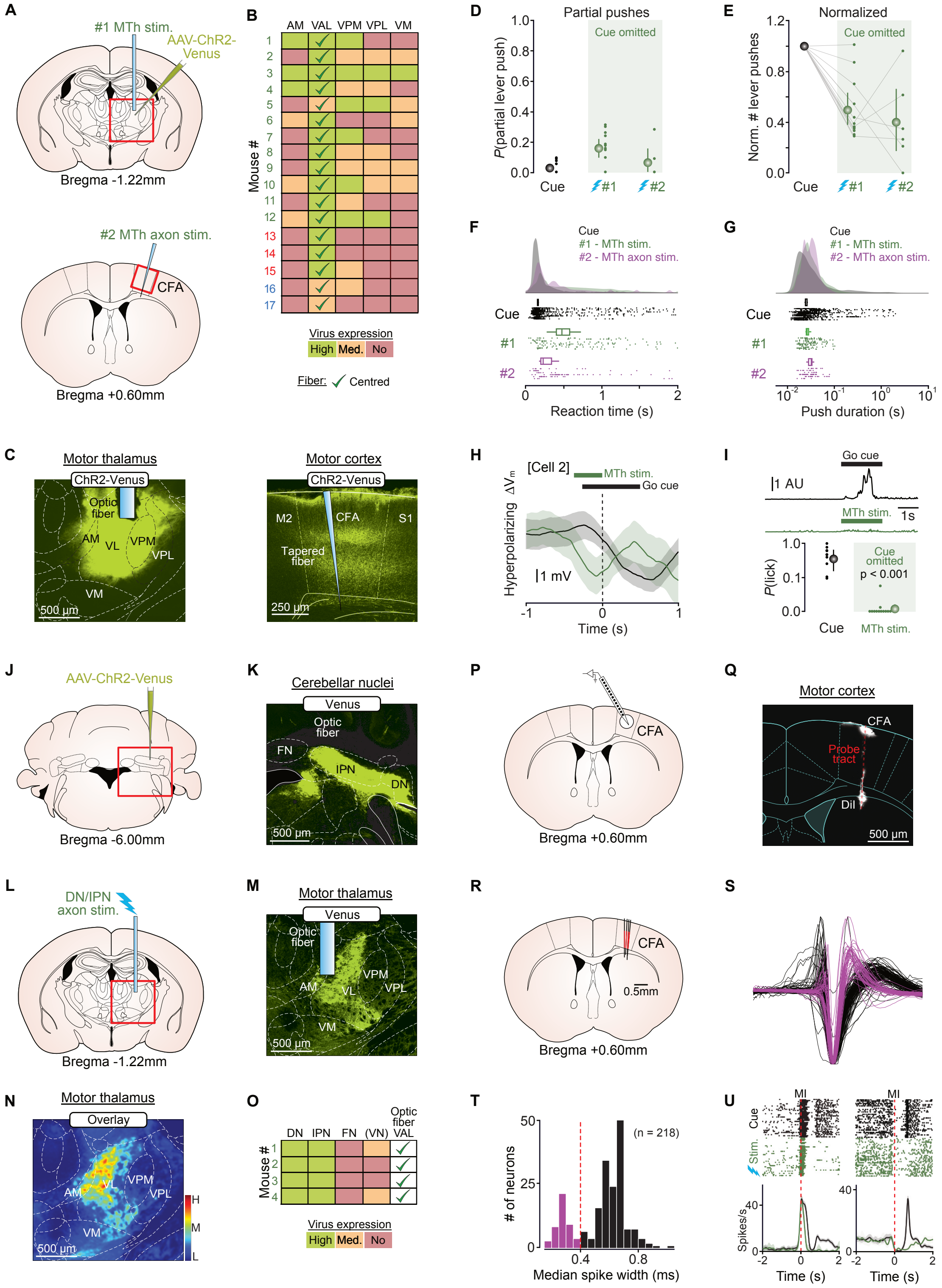

Figure S5. Photoactivation of the cerebello thalamocortical pathway, related to Figure 5.
(A) Injection of AAV-Venus-ChR2 targeted to $MTh_{DN/IPN}$ with optic fiber chronically implanted directly above thalamus (*top, #1*) or a tapered optic fiber acutely implanted into CFA (*bottom, #2*). (B) Quantification of viral expression in ventral motor thalamus (red/no = <5 %, orange/medium = 5-50 % & green/high = 50-100 % expression within each nuclei, green ticks represent correct fiber placement above ventral anterolateral thalamus). Data from mice 1-12 are included in Figure 5B, mice 13-15 displayed no behavioral effects upon photoactivation, mice 16-17 were transduced with AAV-mCherry as controls. (C) Expression of ChR2-Venus and fiber placement in (*left*) ventrolateral thalamus and (*right*) CFA. AM, anteromedial; VL, ventrolateral; VPM, ventral posteromedial; VPL, ventral posterolateral; VM, ventromedial thalamic nuclei. M2, secondary motor cortex; CFA caudal forelimb area; S1, primary sensory cortex. (D) Probability of partial lever push movements evoked by an auditory go cue (black) or photoactivation of $MTh_{DN/IPN}$ (#1) or $MTh_{DN/IPN}$ axons in CFA (#2) in the absence of a go cue (green). Colored dots represent data from individual mice, colored circles represent mean ± 95% CI. For Cue, #1 and #2, N = 12, 12 and 6 mice, respectively. (E) Normalized number of lever pushes evoked by an auditory go cue (black) or photoactivation of $MTh_{DN/IPN}$ (#1) or $MTh_{DN/IPN}$ axons in CFA (#2) in the absence of a go cue (green). Colored dots represent data from individual mice, colored circles represent mean ± 95% CI. For Cue, #1 and #2, N = 12, 12 and 6 mice, respectively. (F-G) Raincloud plots showing the distributions of *(F)* reaction times and *(G)* push durations of cue-evoked (black) and photoactivation (#1 & #2) push trials. Box-and-whisker plots represent bootstrapped estimates of median statistics. (H) Example hyperpolarizing subthreshold V_m change ± 95% CI in a layer 5B projection neuron in response to the cue (black) or photoactivation of $MTh_{DN/IPN}$ (green) in the absence of a cue. Dashed line indicates movement initiation. (I) *Top traces*, average across-trial motion index from an ROI covering the tongue (i.e. licking). Black, cue-evoked licking; green, $MTh_{DN/IPN}$ photoactivation-evoked licking. *Bottom*, probability of licking evoked by an auditory go cue (black) or photoactivation of $MTh_{DN/IPN}$ in the absence of a go cue (green). Colored dots represent data from individual mice, colored circles represent mean ± 95% CI (N = 12 mice). (J) Injection of AAV-Venus-ChR2 targeted to DN/IPN. (K) Expression of ChR2-Venus in DN/IPN. FN, fastigial nucleus; IPN, interpositus nucleus ; DN, dentate nucleus. (L) Photoactivation of DN/IPN axon terminals in $MTh_{DN/IPN}$. (M) Anterograde labelling of DN/IPN axons in motor thalamus and optic fiber placement. (N) Average density of DN/IPN axons across motor thalamic nuclei (N = 4 slices from 4 mice). Scale bar, H - high, M - medium, L - low-level expression. AM, anteromedial; VL, ventrolateral; VPM, ventral posteromedial; VPL, ventral posterolateral; VM, ventromedial thalamic nuclei. (O) Quantification of viral expression (red/no = <5 %, orange/medium = 5-50 % & green/high = 50-100 % expression within each nuclei, green ticks represent correct fiber placement above ventral anterolateral thalamus) (N = 4 mice). FN, fastigial nucleus; IPN, interpositus nucleus ; DN, dentate nucleus. (P) Silicone probe recordings in deep layers of CFA. (Q) Example probe tract in CFA visualized using DiI. (R) Overlay of probe tracts in CFA (N = 4 mice). (S) Overlaid mean spike waveforms for putative interneurons (purple) and pyramidal cells (black). (T) Histogram of spike durations (see methods). (U) Rasters and peri-stimulus time histograms of activity in 2 example deep layer CFA neurons aligned to movement initiation (red dashed line). Black represents cue trials, green represents photoactivation trials.

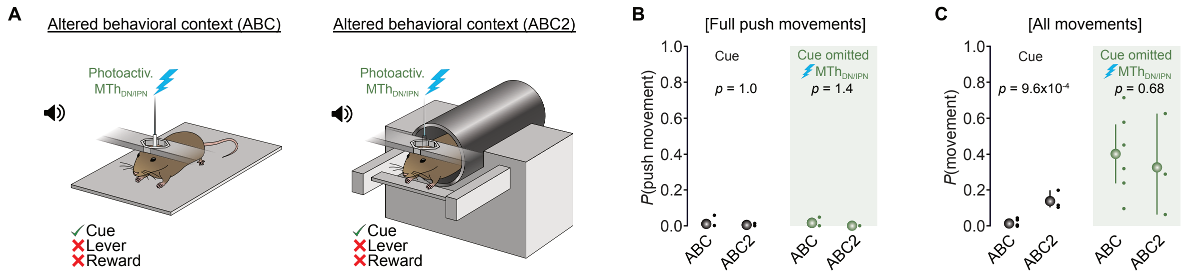

Figure S6. Comparison of photoactivated forelimb movements in two altered behavioral contexts, related to Figure 6.
(A) Photoactivation of $MTh_{DN/IPN}$ in (*left*) an altered behavioral context with flat baseplate (ABC) and (*right*) altered behavioral context that recapitulates LBC mouse posture (ABC2, i.e. horizontal bar positioned at the height of the LBC movable lever - see Figure 6A). (B) Probability of push-like movements evoked by an auditory go cue (black) or photoactivation of $MTh_{DN/IPN}$ in the absence of a go cue (green). Colored dots represent data from individual mice, colored circles represent mean ± 95% CI. ABC, N = 6 mice, ABC2 N = 3 mice. (C) Probability of any forelimb movement evoked by an auditory go cue (black) or photoactivation of $MTh_{DN/IPN}$ in the absence of a go cue (green) in ABC (N = 6 mice) or ABC2 (N = 3 mice).

Table S1. Contributions Matrix

	JD	MC	TC	JA	JS	VCP	FC	JAH	CE	JP	CCH	AH	NR	ID
Conceived and initiated the project	Major	Minor		Minor	Minor							Minor		Major
Anatomical tracing	Major	Major	Major								Major	Major		
In vivo behavior	Major	Major	Major	Major	Major	Major	Major							
In vivo pharmacology	Major						Major							
In vivo imaging		Major			Minor		Minor							
In vivo electrophysiology	Major													
In vivo optogenetics	Major		Major			Major								
Analysed data and produced figures	Major	Major	Major	Minor										Major
Imaging analysis pipelines		Major		Major						Major			Major	
Kinematic tracking								Major	Major					
Supervised the work	Major			Minor	Minor							Minor		Major
Managed the project	Minor													Major
Manuscript writing	Minor													Major
Discussion and interpretation	Major	Major	Major	Major	Major	Major	Major							Major

Major contribution

Minor contribution

No contribution
